# Development of an Inflammation-Related lncRNA-miRNA-mRNA Network Based on Competing Endogenous RNA in Breast Cancer at Single-Cell Resolution

**DOI:** 10.3389/fcell.2022.839876

**Published:** 2022-01-25

**Authors:** Jingxing Liu, Shuyuan Xiao, Jing Chen

**Affiliations:** ^1^ Department of Intensive Care Unit, Changxing People’s Hospital of Zhejiang, Huzhou, China; ^2^ Department of Anesthesiology, The First Affiliated Hospital, College of Medicine, Zhejiang University, Hangzhou, China; ^3^ Department of Oncology, The First Affiliated Hospital of Jiaxing University, Jiaxing, China

**Keywords:** inflammation, breast cancer, LRRC75A-AS1, microRNA (miRNA), prognosis, single cell RNA sequencing (scRNA-seq)

## Abstract

The role and mechanism of inflammation in breast cancer is unclear. This study aims to probe the relationship between inflammation and long non-coding RNAs (lncRNAs) and to stablish an inflammation-related competing endogenous RNA (ceRNA) network in breast cancer. Inflammation-related lncRNAs and target genes were screened based on the data from four single-cell RNA sequencing (scRNA-seq) studies and miRNAs were bioinformatically predicted according to ceRNA hypothesis. A series of *in silico* analyses were performed to construct an inflammation-related ceRNA network in breast cancer. Consequently, a total of seven inflammation-related lncRNAs were selected, after which LRRC75A-AS1 was identified as the most potential lncRNA in view of its expression and prognostic predictive value in breast cancer. Finally, an inflammation-related ceRNA network in breast cancer at the single cell level was established based on lncRNA LRRC75A-AS1, miR-3127-5p, miR-2114-3p, RPL36 and RPL27A mRNAs. Collectively, the lncRNA LRRC75A-AS1 and the LRRC75A-AS1-based on ceRNA network may exert crucial roles in modulating inflammation response during the initiation and progression of breast cancer.

## Introduction

Breast cancer ranks the most common type of human cancer and is also the leading cause of cancer-related deaths in women all over the world (Sung et al., 2021). The prognosis of patients with breast cancer, especially triple-negative breast cancer and HER2-positive breast cancer, is still unsatisfactory, in spite of huge advancements in the aspect of its diagnosis and therapy have been achieved during the past decades (de la Mare et al., 2014). More work and efforts need to be put in the extensive and in-depth research regarding the molecular mechanism of breast carcinogenesis and progression.

When Rudolph Virchow noted leucocytes in tumor tissues in 1863 and proposed the theory of “lymphoreticular infiltrate”, the origin of cancer at sites of inflammation came into researchers’ sight (Balkwill and Mantovani, 2001). After that inflammation has been recognized to contribute to malignant transformation of several types of human malignancies, including hepatocellular carcinoma, colorectal cancer and gastric cancer (Multhoff et al., 2011). Inflammation is also a key component of breast carcinogenesis (Danforth, 2021). However, its roles and molecular mechanism in the initiation and progression of breast cancer is still unclear and need to be further elucidated.

Single-cell RNA sequencing (scRNA-seq) technology, a powerful tool to investigate cell heterogeneity, bringing new strategies to deeply understand the characteristics and behaviors of human cancer (including breast cancer) at the single-cell level (Ren et al., 2021). In this study, we first screened out several inflammation-related lncRNAs in breast cancer using four breast cancer-related scRNA-seq studies, then predicted and analyzed corresponding downstream microRNAs (miRNAs) based on the competing endogenous RNA (ceRNA) hypothesis (Salmena et al., 2011), and finally also identified inflammation-related target gens in breast cancer at the single-cell level. At the end, an inflammation-related lncRNA-miRNA-mRNA triple RNA regulatory network in breast cancer has been established.

## Results

### Identification of LRRC75A-AS1 as a Potential Inflammation-Related Long Non-Coding RNA in Breast Cancer

**FIGURE 1:**
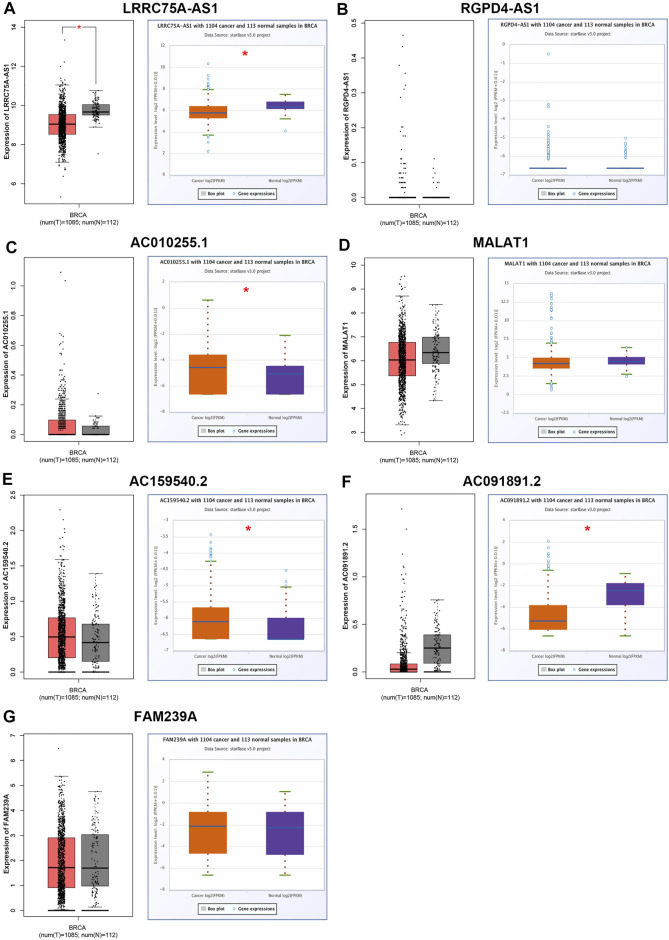
Identification and validation of potential inflammation-related lncRNAs in breast cancer. The expression of LRRC75A-AS1 **(A)**, RGPD4-AS1 **(B)**, AC010255.1 **(C)**, MALAT1 **(D)**, AC159540.2 **(E)**, AC091891.2 **(F)** and FAM239A **(G)** in breast cancer compared with normal controls determined by GEPIA and starBase databases. *p < 0.05.

ScRNA-seq provides an unprecedented opportunity to probe the functional heterogeneity of cancer cells. It has been widely acknowledged that inflammation is closely linked to occurrence and progression of various types of human cancers, including breast cancer. To elucidate the underlying molecular mechanism of the inflammation in breast cancer, four breast cancer-associated scRNA-seq studies were collected (Balavenkatraman et al., 2011; Braune et al., 2016; Jordan et al., 2016; Chung et al., 2017). Consequently, a total of 656 lncRNAs that were significantly associated with inflammation in breast cancer were screened out (Data not shown). Among these lncRNAs, only seven members, consisting of LRRC75A-AS1, RGPD4-AS1, AC010255.1, MALAT1, AC159540.2, AC091891.2 and FAM239A, appear in more than one scRNA-seq studies. As listed in Table 1, all the seven lncRNAs were negatively correlated with inflammation in breast cancer. Next, we successively determined the expression levels of the seven lncRNAs in breast cancer by GEPIA database and starBase database. As presented in Figure 1, only LRRC75A-AS1 expression was markedly decreased in breast cancer when compared with normal controls in both two databases. These findings suggested that LRRC75A-AS1 might be the most potential inflammation-related lncRNA in breast cancer.

**TABLE 1:** The potential lncRNAs significantly associated with inflammation in breast cancer.

LncRNA	Correlation efficient		
	EXP0052	EXP0053	EXP0054
LRRC75A-AS1	−0.311	−0.340	—
RGPD4-AS1	−0.313	−0.321	—
AC010255.1	−0.464	—	−0.548
MALAT1	−0.469	−0.321	—
AC159540.2	−0.369	—	−0.349
AC091891.2	−0.539	—	−0.422
FAM239A	—	−0.318	−0.356

### Survival Analysis for LRRC75A-AS1 in Various Immunocyte-Enriched/Decreased Breast Cancer

**FIGURE 2:**
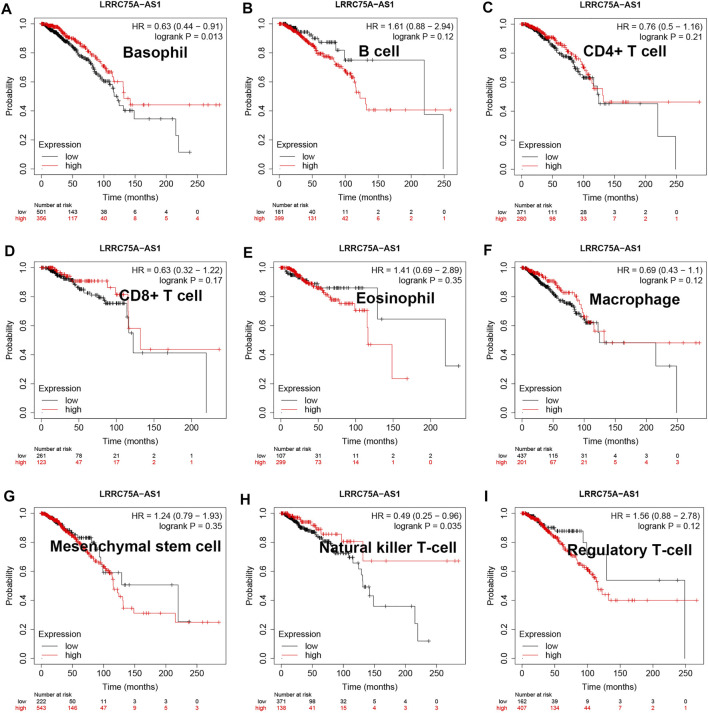
Survival analysis for LRRC75A-AS1 in breast cancer enriched with various immunocyte. **(A)** The prognostic value of LRRC75A-AS1 in basophil-enriched breast cancer. **(B)** The prognostic value of LRRC75A-AS1 in B cell-enriched breast cancer. **(C)** The prognostic value of LRRC75A-AS1 in CD4^+^ cell-enriched breast cancer. **(D)** The prognostic value of LRRC75A-AS1 in CD8^+^ cell-enriched breast cancer. **(E)** The prognostic value of LRRC75A-AS1 in eosinophil-enriched breast cancer. **(F)** The prognostic value of LRRC75A-AS1 in macrophage-enriched breast cancer. **(G)** The prognostic value of LRRC75A-AS1 in mesenchymal stem cell-enriched breast cancer. **(H)** The prognostic value of LRRC75A-AS1 in natural killer T cell-enriched breast cancer. **(I)** The prognostic value of LRRC75A-AS1 in regulatory T cell-enriched breast cancer.

**FIGURE 3:**
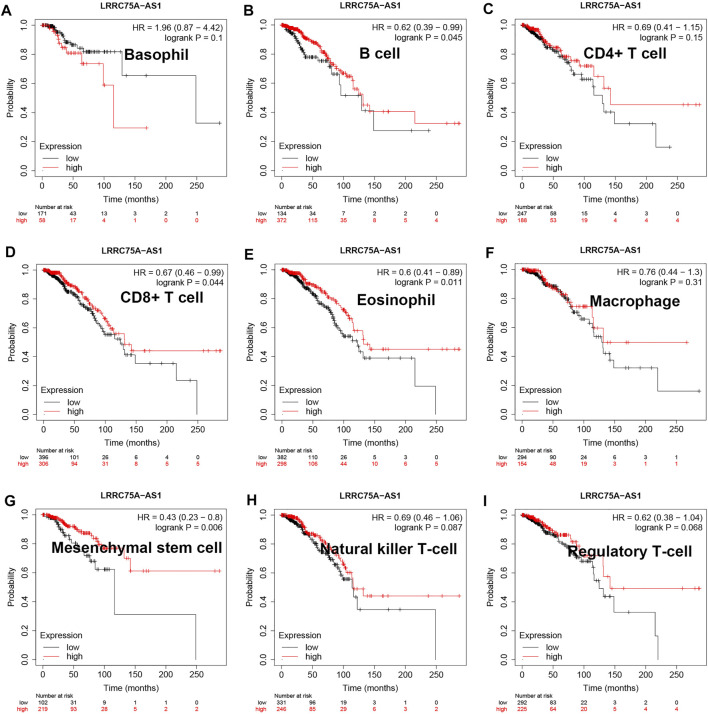
Survival analysis for LRRC75A-AS1 in breast cancer decreased with various immunocyte. **(A)** The prognostic value of LRRC75A-AS1 in basophil-decreased breast cancer. **(B)** The prognostic value of LRRC75A-AS1 in B cell-decreased breast cancer. **(C)** The prognostic value of LRRC75A-AS1 in CD4^+^ cell-decreased breast cancer. **(D)** The prognostic value of LRRC75A-AS1 in CD8^+^ cell-decreased breast cancer. **(E)** The prognostic value of LRRC75A-AS1 in eosinophil-decreased breast cancer. **(F)** The prognostic value of LRRC75A-AS1 in macrophage-decreased breast cancer. **(G)** The prognostic value of LRRC75A-AS1 in mesenchymal stem cell-decreased breast cancer. **(H)** The prognostic value of LRRC75A-AS1 in natural killer T cell-decreased breast cancer. **(I)** The prognostic value of LRRC75A-AS1 in regulatory T cell-decreased breast cancer.

As mentioned above, LRRC75A-AS1 was identified as a potential inflammation-related lncRNA in breast cancer. As is known to all, inflammation and immunity are two ends of the balance in human body. Thus, we performed survival analysis for inflammation-related LRRC75A-AS1 in breast cancer enriched or decreased with various immunocytes. Nine different immunocytes, involving basophil, B cell, CD4^+^ T cell, CD8^+^ T cell, eosinophil, macrophage, mesenchymal stem cell, natural killer T cell and regulatory T cell, were included for this survival analysis. As shown in Figure 2, high expression of LRRC75A-AS1 indicated favorable overall survival in breast cancer enriched with basophil or natural killer T cell. Moreover, upregulation of LRRC75A-AS1 was positively correlated with good overall survival in breast cancer decreased with B cell, CD8^+^ T cell, eosinophil or mesenchymal stem cell (Figure 3). No significant prognostic values of LRRC75A-AS1 in breast cancer enriched or decreased with other immunocytes were observed. Taken together, some immunocytes, including basophil, natural killer T cell, B cell, CD8^+^ T cell, eosinophil or mesenchymal stem cell, might be involved in interaction with LRRC75A-AS1-mediated inflammation in breast cancer.

### Prediction and Analysis of Downstream Potential miRNAs of LRRC75A-AS1 in Breast Cancer

**FIGURE 4:**
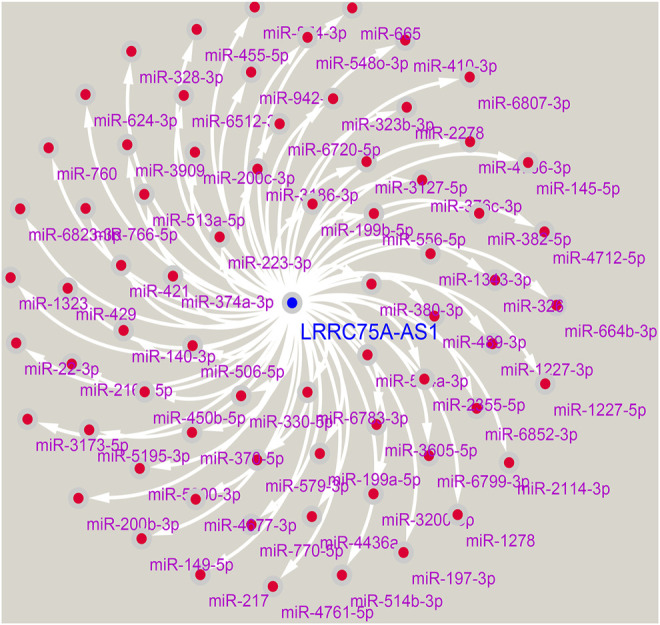
Construction of a LRRC75A-AS1-miRNA regulatory.

**FIGURE 5:**
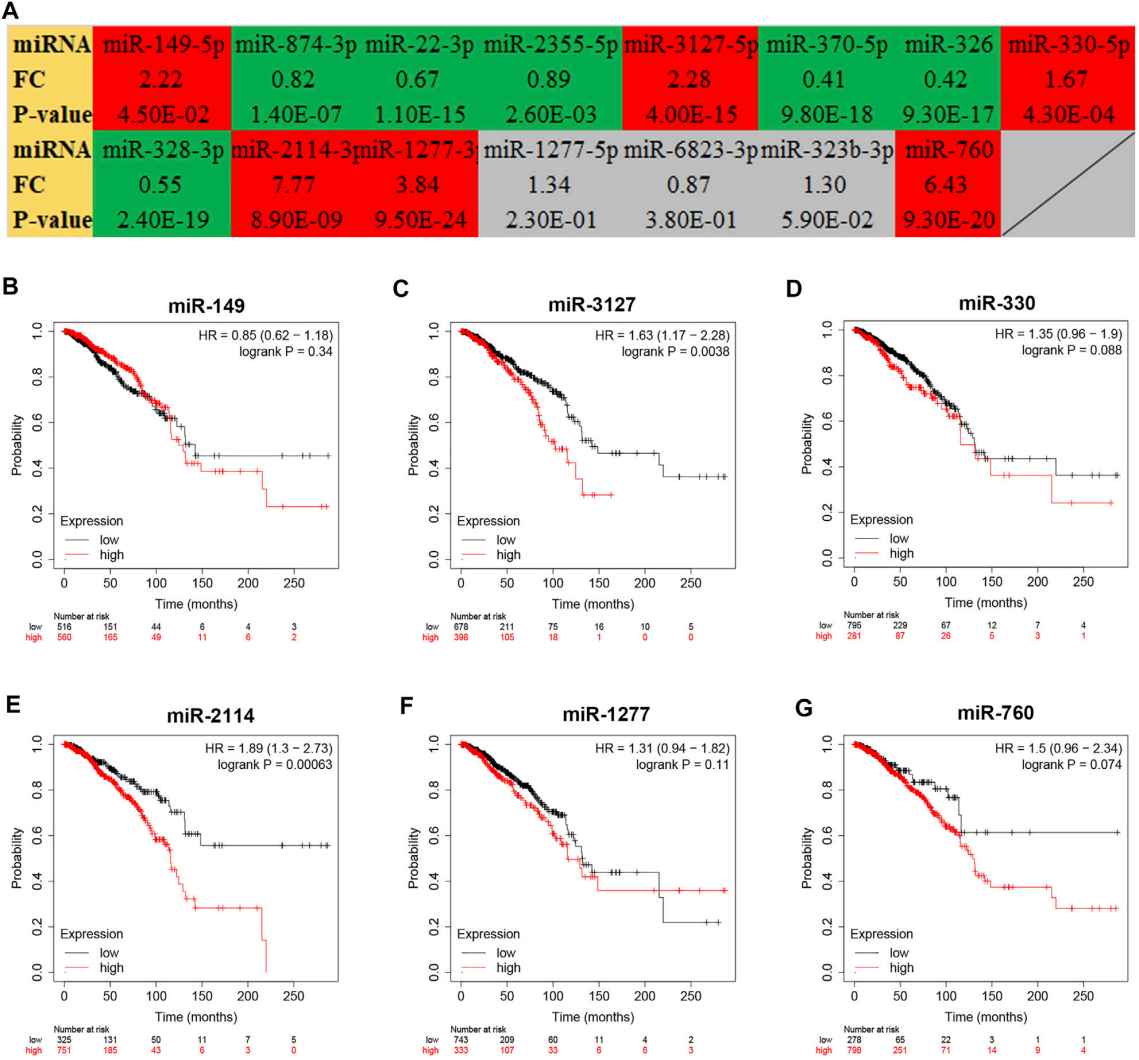
Expression analysis and survival analysis for the miRNAs negatively correlated with LRRC75A-AS1 in breast cancer. **(A)** The expression levels of 15 LRRC75A-AS1-related miRNAs in breast cancer detected by starBase database. Red: overexpressed in breast cancer; Green: downregulated in breast cancer; gray: no significant differences between breast cancer and normal controls. **(B–G)** The prognostic value of miR-149-5p **(B)**, miR-3127-5p **(C)**, miR-330-5p **(D)**, miR-2114-3p **(E)** and miR-1277-3p **(F)** and miR-760 **(G)** in breast cancer.

To further explore the downstream action mechanism of inflammation-related LRRC75A-AS1 in breast cancer, miRNet was employed to predict the miRNAs that potentially bind to LRRC75A-AS1. A total of 70 miRNAs were identified. For better visualization, a LRRC75A-AS1-miRNA network was established using Cytoscape software (Figure 4). According to competing endogenous RNA (ceRNA) hypothesis, these should be negative expression correlation between lncRNA and miRNA. Therefore, expression correlation of LRRC75A-AS1 with its predicted miRNAs in breast cancer was calculated using TCGA breast cancer data. As listed in Table 2, 15 of 70 miRNAs were significantly inversely associated with LRRC75A-AS1 in breast cancer. Then, we further analyzed the expression levels of the 15 negatively correlated miRNAs in breast cancer using starBase database. As presented in Figure 5A, 6 miRNAs were significantly downregulated in breast cancer tissues compared with normal breast tissues, including miR-149-5p, miR-3127-5p, miR-330-5p, miR-2114-3p, miR-1277-3p and miR-760. The prognostic values of the overexpressed six miRNAs in breast cancer were also assessed by Kaplan-Meier plotter database. Only breast cancer patients with high expression of miR-3127-5p or miR-2114-3p had poor prognosis. By combination of correlation analysis, expression analysis and survival analysis, miR-3127-5p and miR-2114-3p might be two most potential downstream binding miRNAs of LRRC75A-AS1 in breast cancer.

**TABLE 2:** The expression correlation of LRRC75A-AS1 with predicted miRNAs in breast cancer determined by starBase database.

lncRNA	miRNA	R-value	*p*-value
**LRRC75A-AS1**	**miR-149-5p**	**−0.131**	**1.45E-05**
**LRRC75A-AS1**	**miR-874-3p**	**−0.114**	**1.63E-04**
**LRRC75A-AS1**	**miR-22-3p**	**−0.104**	**5.71E-04**
**LRRC75A-AS1**	**miR-2355-5p**	**−0.101**	**8.70E-04**
**LRRC75A-AS1**	**miR-3127-5p**	**−0.101**	**9.01E-04**
**LRRC75A-AS1**	**miR-370-5p**	**−0.086**	**4.61E-03**
**LRRC75A-AS1**	**miR-326**	**−0.084**	**5.48E-03**
**LRRC75A-AS1**	**miR-330-5p**	**−0.080**	**8.20E-03**
**LRRC75A-AS1**	**miR-328-3p**	**−0.077**	**1.14E-02**
**LRRC75A-AS1**	**miR-2114-3p**	**−0.071**	**1.93E-02**
**LRRC75A-AS1**	**miR-1277-3p**	**−0.065**	**3.15E-02**
**LRRC75A-AS1**	**miR-1277-5p**	**−0.065**	**3.28E-02**
**LRRC75A-AS1**	**miR-6823-3p**	**−0.065**	**3.27E-02**
**LRRC75A-AS1**	**miR-323b-3p**	**−0.063**	**3.69E-02**
**LRRC75A-AS1**	**miR-760**	**−0.060**	**4.99E-02**
LRRC75A-AS1	miR-145-5p	**−**0.051	9.63E-02
LRRC75A-AS1	miR-382-5p	**−**0.051	9.16E-02
LRRC75A-AS1	miR-489-3p	**−**0.049	1.06E-01
LRRC75A-AS1	miR-6799-3p	**−**0.049	1.05E-01
LRRC75A-AS1	miR-4766-3p	**−**0.047	1.20E-01
LRRC75A-AS1	miR-2278	**−**0.040	1.85E-01
LRRC75A-AS1	miR-3909	**−**0.040	1.88E-01
LRRC75A-AS1	miR-665	**−**0.039	2.02E-01
LRRC75A-AS1	miR-4712-5p	**−**0.037	2.19E-01
LRRC75A-AS1	miR-942-5p	**−**0.037	2.26E-01
LRRC75A-AS1	miR-514b-3p	**−**0.033	2.75E-01
LRRC75A-AS1	miR-624-3p	**−**0.032	2.95E-01
LRRC75A-AS1	miR-3200-5p	**−**0.029	3.40E-01
LRRC75A-AS1	miR-197-3p	**−**0.026	3.94E-01
LRRC75A-AS1	miR-4436a	**−**0.023	4.55E-01
LRRC75A-AS1	miR-421	**−**0.020	5.02E-01
LRRC75A-AS1	miR-3173-5p	**−**0.017	5.78E-01
LRRC75A-AS1	miR-766-5p	**−**0.016	5.89E-01
LRRC75A-AS1	miR-548o-3p	**−**0.014	6.55E-01
LRRC75A-AS1	miR-199a-5p	**−**0.012	6.87E-01
LRRC75A-AS1	miR-6852-3p	**−**0.012	6.88E-01
LRRC75A-AS1	miR-374a-3p	**−**0.011	7.11E-01
LRRC75A-AS1	miR-579-3p	**−**0.011	7.08E-01
LRRC75A-AS1	miR-1343-3p	**−**0.010	7.51E-01
LRRC75A-AS1	miR-199b-5p	**−**0.010	7.37E-01
LRRC75A-AS1	miR-3605-5p	**−**0.009	7.57E-01
LRRC75A-AS1	miR-429	**−**0.007	8.24E-01
LRRC75A-AS1	miR-4761-5p	**−**0.007	8.16E-01
LRRC75A-AS1	miR-376c-3p	**−**0.004	8.87E-01
LRRC75A-AS1	miR-4677-3p	0.002	9.59E-01
LRRC75A-AS1	miR-664b-3p	0.002	9.51E-01
LRRC75A-AS1	miR-6783-3p	0.003	9.26E-01
LRRC75A-AS1	miR-450b-5p	0.005	8.63E-01
LRRC75A-AS1	miR-514a-3p	0.006	8.46E-01
LRRC75A-AS1	miR-5195-3p	0.006	8.37E-01
LRRC75A-AS1	miR-6807-3p	0.012	6.94E-01
LRRC75A-AS1	miR-5000-3p	0.013	6.68E-01
LRRC75A-AS1	miR-506-5p	0.015	6.20E-01
LRRC75A-AS1	miR-6720-5p	0.019	5.35E-01
LRRC75A-AS1	miR-513a-5p	0.021	4.88E-01
LRRC75A-AS1	miR-140-3p	0.032	2.94E-01
LRRC75A-AS1	miR-200b-3p	0.033	2.70E-01
LRRC75A-AS1	miR-556-5p	0.033	2.75E-01
LRRC75A-AS1	miR-380-3p	0.034	2.69E-01
LRRC75A-AS1	miR-216a-5p	0.037	2.23E-01
LRRC75A-AS1	miR-1278	0.038	2.05E-01
LRRC75A-AS1	miR-223-3p	0.041	1.74E-01
LRRC75A-AS1	miR-410-3p	0.045	1.40E-01
LRRC75A-AS1	miR-6512-3p	0.047	1.22E-01
LRRC75A-AS1	miR-3186-3p	0.049	1.07E-01
LRRC75A-AS1	miR-770-5p	0.051	9.32E-02
LRRC75A-AS1	miR-1323	0.068	2.43E-02
LRRC75A-AS1	miR-200c-3p	0.073	1.59E-02
LRRC75A-AS1	miR-217	0.074	1.43E-02
LRRC75A-AS1	miR-455-5p	0.127	2.59E-05

### Identification of Downstream Inflammation-Related Targets of LRRC75A-AS1-miRNA Pathways in Breast Cancer

**FIGURE 6:**
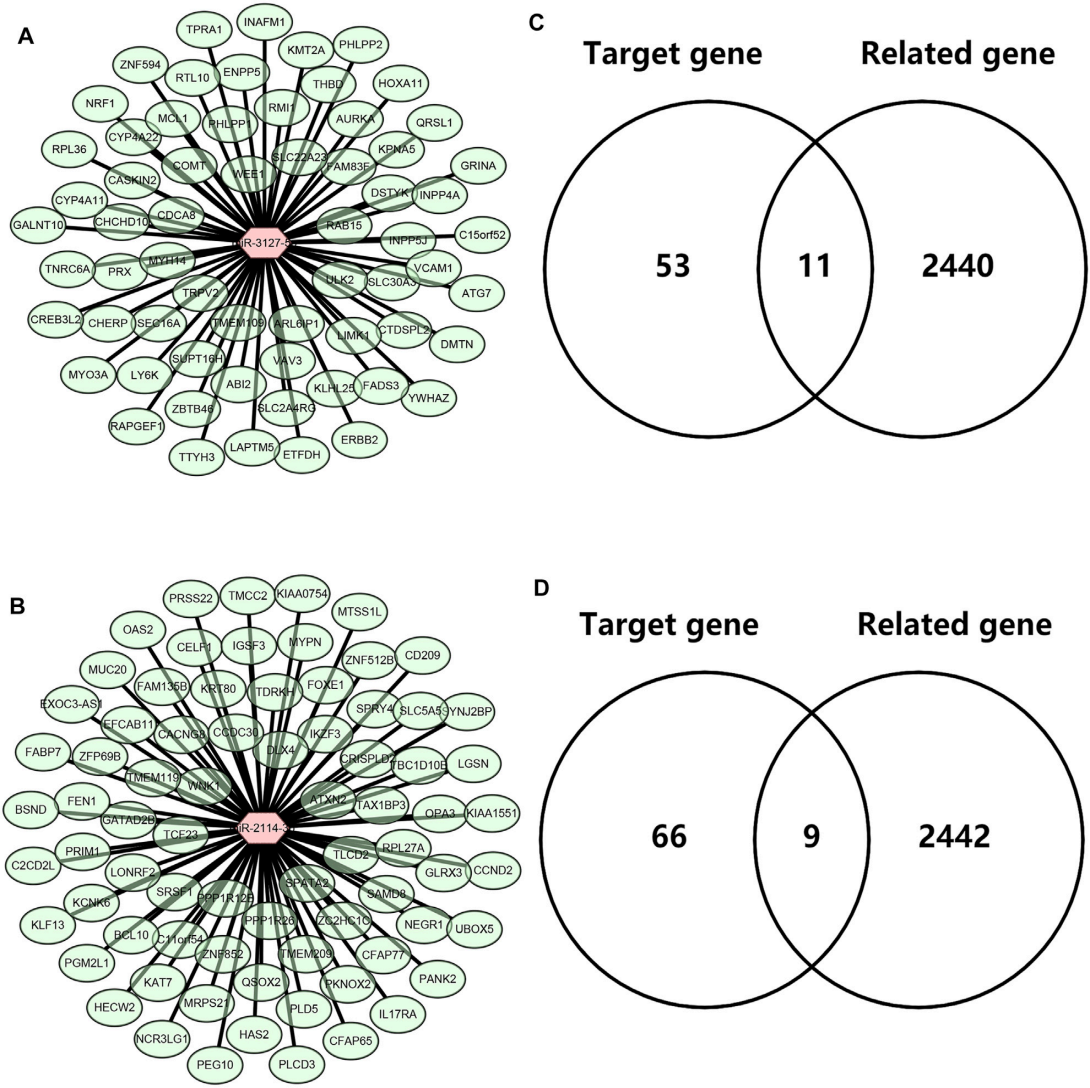
Identification of downstream potential targets of miR-3127-5p and miR-2114-3p in breast cancer. **(A)** Establishment of a miR-3127-5p-gene network. **(B)** Establishment of a miR-2114-3p-gene network. **(C,D)** Intersection of predicted target genes of miR-3127-5p **(C)** or miR-2114-3p **(D)** and inflammation-related genes in breast cancer.

Next, we intended to explore the downstream molecular mechanism of inflammation-related LRRC75A-AS1-miR-3127-5p/miR-2114-3p pathways in breast cancer. First of all, these genes that were significantly associated with inflammation in breast cancer were obtained by analyzing the four scRNA-seq studies as mentioned above. 2,452 inflammation-related genes were identified (Data not shown). Moreover, as is known to all, miRNAs usually exert their roles by suppressing target genes. Thus, the potential target genes of miR-3127-5p or miR-2114-3p were predicted by miRNet database. 64 and 75 genes were forecasted to be downstream targets of miR-3127-5p and miR-2114-3p, respectively. For better visualization, miR-3127-5p-gene and miR-2114-3p-gene networks were established as shown in Figure 6A and Figure 6B. By intersection of target genes and inflammation-related genes, 11 and 9 potential inflammation-related target genes of miR-3127-5p and miR-2114-3p were respectively identified as presented in Figures 6C,D.

### Construction of an Inflammation-Related LRRC75A-AS1-miRNA-mRNA Network in Breast Cancer

**FIGURE 7:**
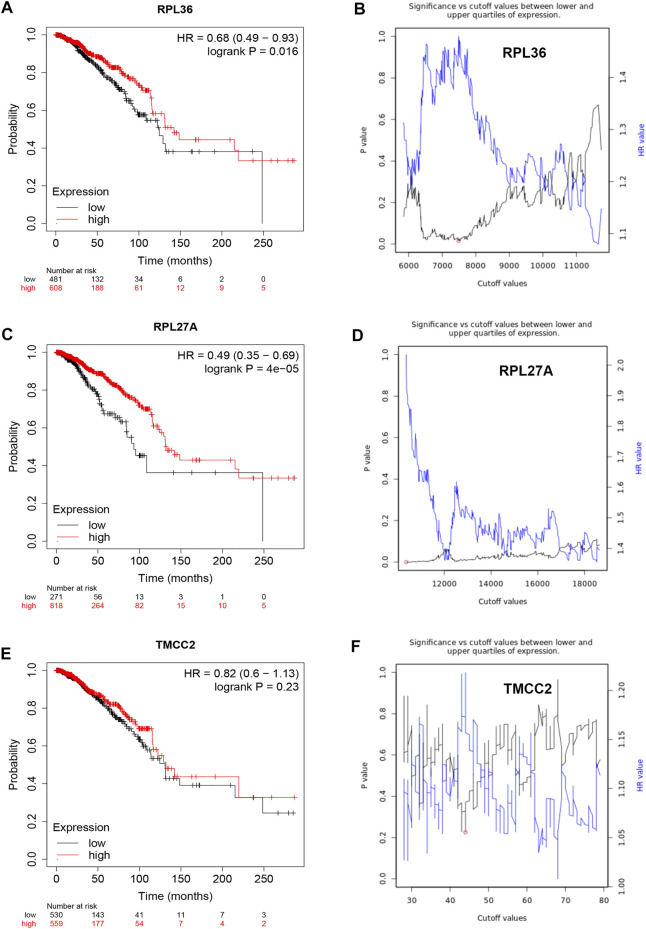
Survival analysis for RPL36, RPL27A and TMCC2 in breast cancer. **(A)** The prognostic value of RPL36 in breast cancer. **(B)** The plot of significance versus cutoff values between lower and upper quartiles of RPL36 expression. **(C)** The prognostic value of RPL27A in breast cancer. **(D)** The plot of significance versus cutoff values between lower and upper quartiles of RPL27A expression. **(E)** The prognostic value of TMCC2 in breast cancer. **(F)** The plot of significance versus cutoff values between lower and upper quartiles of TMCC2 expression.

**FIGURE 8:**
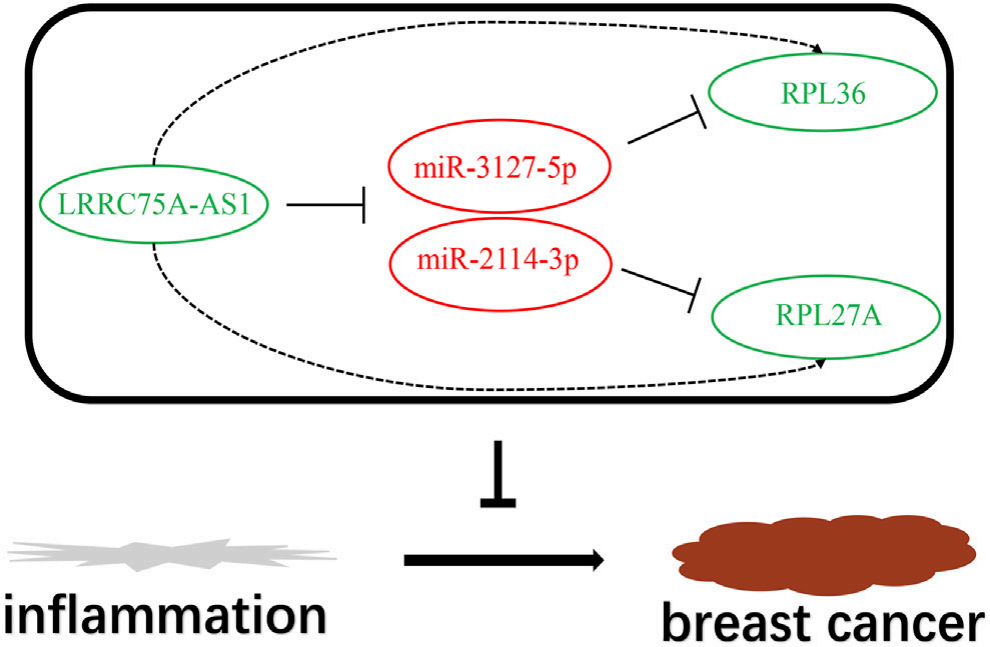
The model of LRRC75A-AS1-mediated inflammation-related miRNA-mRNA pathways in breast cancer.

Based on the negative regulation mechanism of miRNA, expression correlation analysis was conducted. As listed in Table 3, 3 of 11 target genes (DMTN, MCL1 and RPL36) were significantly inversely correlated with miR-3127-5p expression in breast cancer. And two target genes (ULK2 and RPL36) were significantly positively correlated with LRRC75A-AS1 expression in breast cancer. Thus, RPL36 might be the most potential inflammation-related target gene of LRRC75A-AS1/miR-3127-5p pathway in breast cancer. In the same light, three target genes (FABP7, RPL27A and TMCC2) were markedly negatively associated with miR-2114-3p expression in breast cancer and two target genes (RPL27A and TMCC2) were markedly positively linked to LRRC75A-AS1 expression in breast cancer as shown in Table 4. Therefore, RPL27A and TMCC2 might be two potential inflammation-related target genes of LRRC75A-AS1/miR-2114-3p pathway in breast cancer. Finally, the prognostic values of RPL36, RPL27A and TMCC2 in breast cancer were evaluated using Kaplan-Meier plotter database. As presented in Figure 7, high expression of RPL36 and RPL27A indicated favorable prognosis in breast cancer. No statistical role of TMCC2 in predicting prognosis of breast cancer was observed. Taken together, an inflammation-related LRRC75A-AS1-mediated miRNA-mRNA pathways in breast cancer were identified as vividly depicted in Figure 8.

**TABLE 3:** The expression correlation of miR-3127-5p with target genes or target genes with LRRC75A-AS1 in breast cancer.

Target gene	miR-3127-5p		LRRC75A-AS1	
	R-value	*p*-value	R-value	*p*-value
DMTN	**−**0.148	1.02E-06	0.062	3.81E-02
ERBB2	0.045	1.41E-01	**−**0.190	2.08E-10
ETFDH	**−**0.040	1.85E-01	0.004	9.00E-01
GRINA	0.230	1.74E-14	**−**0.074	1.36E-02
MCL1	**−**0.182	1.65E-09	0.034	2.59E-01
LAPTM5	**−**0.070	2.10E-02	**−**0.113	1.68E-04
ULK2	0.044	1.50E-01	0.180	1.83E-09
VAV3	0.008	7.82E-01	**−**0.087	3.93E-03
SUPT16H	0.273	6.13E-20	**−**0.011	7.08E-01
***RPL36***	**−*0.151***	***6.34E-07***	***0.435***	***2.78E-52***
QRSL1	0.079	9.04E-03	0.020	5.13E-01

**TABLE 4:** The expression correlation of miR-2114-3p with target genes or target genes with LRRC75A-AS1 in breast cancer.

Target gene	miR-2114-3p		LRRC75A-AS1	
	R-value	*p*-value	R-value	*p*-value
FABP7	**−**0.125	3.82E-05	0.056	6.31E-02
***RPL27A***	**−*0.084***	***5.77E-03***	***0.472***	***2.61E-62***
***TMCC2***	**−*0.098***	***1.25E-03***	***0.108***	***3.45E-04***
KAT7	0.109	3.41E-04	**−**0.104	5.23E-04
SYNJ2BP	0.104	5.90E-04	**−**0.165	3.85E-08
TMEM209	0.030	3.31E-01	**−**0.043	1.52E-01
EFCAB11	0.167	3.15E-08	**−**0.058	5.39E-02
NEGR1	0.061	4.59E-02	**−**0.004	8.90E-01
TMEM119	0.039	2.03E-01	**−**0.049	1.02E-01

## Discussion

Despite inflammation is present in breast cancer and may affect its outcome, the molecular mechanism of inflammation in breast carcinogenesis is still unknown. In this study, we aimed to identify a lncRNA-miRNA-mRNA regulatory network based on ceRNA hypothesis at single-cell level.

CancerSEA was employed to identify inflammation-related lncRNAs in breast cancer at single-cell resolution. By integrated analysis of lncRNAs’ expression, LRRC75A-AS1 was identified as the most potential inflammation-associated lncRNA in breast cancer. Several studies have confirmed its key roles in human cancer. For example, Pang et al. suggested that LRRC75A-AS1 suppressed multiple myeloma by targeting miR-199b-5p/PDCD4 axis (Pang et al., 2020); Chen et al. demonstrated that LRRC75A-AS1 repressed cell proliferation and migration of colorectal carcinoma (Chen et al., 2019); Li et al. confirmed that LRRC75A-AS1 facilitated cell proliferation and invasion of triple negative breast cancer by acting as a ceRNA to regulate BAALC (Li et al., 2020). Tumor infiltrating lymphocytes (TILs) are closely linked to prognosis of cancer patients (Yu S et al., 2020; Lou et al., 2021). Moreover, survival analysis also revealed that LRRC75A-AS1 possessed significant prognostic value in breast cancer enriched with basophil, natural killer T cell, or decreased with B cell, CD8^+^ T cell, eosinophil or mesenchymal stem cell.

ceRNA mechanism can partially account for lncRNA’s action mechanism (Lou et al., 2020). Using miRNet database, a series of miRNAs were predicted. By combination of correlation analysis, expression analysis and survival analysis, miR-3127-5p and miR-2114-3p were selected as the two most potential downstream binding miRNAs of LRRC75A-AS1 in breast cancer. Both of the two miRNAs have been reported to be involved in initiation and progression of human malignancies, including osteosarcoma (Wang et al., 2021), papillary thyroid cancer (Zhou et al., 2021), colorectal cancer (Ma et al., 2021) and ovarian cancer (Yu Z et al., 2020).

Subsequently, the downstream target genes of miR-3127-5p or miR-2114-3p were predicted by online target gene prediction tool. Moreover, the inflammation-related genes in breast cancer were also identified using four breast cancer scRNA-seq studies as mentioned above. By intersection of target gene set and inflammation-related gene sets, 11 and 9 candidate genes were screened out for miR-3127-5p and miR-2114-3p, respectively. After performing correlation analysis and survival analysis, RPL36 was identified as the most potential target for miR-3127-5p and RPL27A was for miR-2114-3p.

RPL36 was reported as a tumor suppressor to restrain KRAS-induced pancreatic cancer (Provost et al., 2014), and was a promising prognostic marker in hepatocellular carcinoma (Song et al., 2011). RPL27A was also found to be involved in development and metastasis of triple-negative breast cancer in both mouse and human (Zhao et al., 2021). Our analytic results together with these reports indicated that RPL36 and RPL27A might be downstream key targets of LRRC75A-AS1/miR-3127-5p/miR-2114-3p pathways in breast cancer.

At the end, a novel inflammation-related lncRNA-miRNA-mRNA regulatory network in breast cancer was successively established based on ceRNA hypothesis and scRNA-seq data. There were several limitations in this study. For example, the miRNAs in this network were not identified using scRNA-seq data; the results from this study were only based on *in silico* analysis. However, this is the first study to comprehensively explore inflammation-related lncRNA-miRNA-mRNA network in breast cancer. All these findings need to be further validated using basic experiments and clinical trials in the future.

## Materials and Methods

### Screening of Inflammation-Related Long Non-Coding RNAs and Genes

CancerSEA (http://biocc.hrbmu.edu.cn/CancerSEA/home.jsp) is the first dedicated database that aims to comprehensively decode distinct functional states of cancer cells at single-cell resolution (Yuan et al., 2019). In this study, CancerSEA database was employed to identify inflammation-related lncRNAs and genes in breast cancer. Only those lncRNAs and genes that were significantly associated with inflammation in breast cancer were included. *p*-value < 0.05 was considered as statistically significant.

### Expression Analysis for Long Non-Coding RNA

Two databases, including GEPIA (http://gepia.cancer-pku.cn/) and starBase (http://starbase.sysu.edu.cn/), were used to analyze the expression of lncRNAs in breast cancer (Li et al., 2014; Tang et al., 2017). Only lncRNAs that were significantly upregulated or downregulated in both two databases were selected for subsequent analysis. *p*-value < 0.05 was considered as statistically significant.

### Survival Analysis for Long Non-Coding RNA

The prognostic values of LRRC75A-AS1 in breast cancer were determined using Kaplan-Meier plotter (http://kmplot.com/analysis/), which is an online database capable of accessing the effects of genes or miRNAs on survival in more than 20 cancer types including breast cancer (Lou et al., 2021). Logrank *p*-value < 0.05 was considered as statistically significant.

### MicroRNA Prediction

miRNet (http://www.mirnet.ca), a database for miRNA functional analysis and systems biology, was used to predict the binding miRNAs of LRRC75A-AS1 (Chang et al., 2020). Then, a LRRC75A-AS1-miRNA network was established using Cytoscape software.

### Expression and Survival Analysis for MicroRNA

The expression levels of miRNAs of LRRC75A-AS1 in breast cancer were determined by usage of starBase (http://starbase.sysu.edu.cn/). *p*-value < 0.05 was considered as statistically significant. Kaplan-Meier plotter (http://kmplot.com/analysis/) was employed to assess the prognostic values of miRNAs of LRRC75A-AS1. The survival plots were downloaded from the website. Logrank *p*-value < 0.05 was considered as statistically significant.

### Target Gene Prediction

miRNet (http://www.mirnet.ca) was introduced to predict the downstream target genes of miR-3127-5p and miR-2114-3p. Then, a miR-3127-5p-target gene network or miR-2114-3p-target gene network was constructed using Cytoscape software.

### Intersection Analysis

VENNY 2.1 (https://bioinfogp.cnb.csic.es/tools/venny/index.html) was employed to perform intersection analysis for target genes of miRNAs and inflammation-related genes in breast cancer. Only the genes that commonly appeared in both two gene sets were included for subsequent analysis.

### Correlation Analysis

starBase (http://starbase.sysu.edu.cn/) was used to conduct expression correlation analysis for LRRC75A-AS1-miRNA, miRNA-target gene or LRRC75A-AS1-target gene pairs in breast cancer. Only those RNA-RNA pairs with *p*-value less than 0.05 were considered as statistically significant.

### Statistical Analysis

The statistical analyses were directly performed using online databases or tools as mentioned above. *p*-value < 0.05 or logrank *p*-value < 0.05 was considered as statistically significant.

## Data Availability

The original contributions presented in the study are included in the article/Supplementary Material, further inquiries can be directed to the corresponding author.
