# Supplementary material for: Development of an Inflammation-Related lncRNA-miRNA-mRNA Network Based on Competing Endogenous RNA in Breast Cancer at Single-Cell Resolution
Source: Front Cell Dev Biol. 2022 Jan 25;10:839876. doi: 10.3389/fcell.2022.839876 (PMC8821924; doi:10.3389/fcell.2022.839876)
Supplement: Supplementary file 1 [file Table1.DOCX]

**Table S1**. The lncRNAs associated with inflammation in breast cancer obtained from cancerSEA database.

| Ensemble ID | Symbol | No. dataset | Direction |
| --- | --- | --- | --- |
| ENSG00000175061 | LRRC75A-AS1 | 2 | negative |
| ENSG00000230651 | RGPD4-AS1 | 2 | negative |
| ENSG00000247311 | AC010255.1 | 2 | negative |
| ENSG00000251562 | MALAT1 | 2 | negative |
| ENSG00000277701 | AC159540.2 | 2 | negative |
| ENSG00000281560 | AC091891.2 | 2 | negative |
| ENSG00000234449 | FAM239A | 2 | negative |
| ENSG00000181908 | AP003774.1 | 1 | positive |
| ENSG00000224184 | MIR3681HG | 1 | positive |
| ENSG00000241570 | PAQR9-AS1 | 1 | positive |
| ENSG00000255237 | AC138230.1 | 1 | positive |
| ENSG00000255252 | AL078612.1 | 1 | positive |
| ENSG00000255670 | AC007619.1 | 1 | positive |
| ENSG00000258092 | AC005841.1 | 1 | positive |
| ENSG00000259354 | AC025580.2 | 1 | positive |
| ENSG00000269888 | AC112491.1 | 1 | positive |
| ENSG00000269968 | AC006064.4 | 1 | positive |
| ENSG00000271551 | AL355297.4 | 1 | positive |
| ENSG00000279296 | PRAL | 1 | positive |
| ENSG00000283029 | AL139099.4 | 1 | positive |
| ENSG00000228830 | AL160408.2 | 1 | positive |
| ENSG00000231298 | LINC00704 | 1 | positive |
| ENSG00000231817 | LINC01198 | 1 | positive |
| ENSG00000236883 | AP001615.1 | 1 | positive |
| ENSG00000245768 | AC092378.1 | 1 | positive |
| ENSG00000253123 | AC091182.1 | 1 | positive |
| ENSG00000253398 | AC021733.1 | 1 | positive |
| ENSG00000257495 | KRT73-AS1 | 1 | positive |
| ENSG00000259884 | AC025259.3 | 1 | positive |
| ENSG00000261122 | LINC02167 | 1 | positive |
| ENSG00000267165 | CHMP1B-AS1 | 1 | positive |
| ENSG00000268568 | AC007228.2 | 1 | positive |
| ENSG00000276255 | AL136379.1 | 1 | positive |
| ENSG00000277539 | AC138058.1 | 1 | positive |
| ENSG00000278929 | AP001784.1 | 1 | positive |
| ENSG00000279753 | AC011558.1 | 1 | positive |
| ENSG00000280800 | FP671120.3 | 1 | positive |
| ENSG00000172965 | MIR4435-2HG | 1 | positive |
| ENSG00000175746 | C15orf54 | 1 | positive |
| ENSG00000215067 | ALOX12-AS1 | 1 | positive |
| ENSG00000223855 | AC147651.1 | 1 | positive |
| ENSG00000225761 | AL596247.1 | 1 | positive |
| ENSG00000225936 | AL731557.1 | 1 | positive |
| ENSG00000226777 | FAM30A | 1 | positive |
| ENSG00000227165 | WDR11-AS1 | 1 | positive |
| ENSG00000229047 | AF127577.1 | 1 | positive |
| ENSG00000230630 | DNM3OS | 1 | positive |
| ENSG00000230724 | LINC01001 | 1 | positive |
| ENSG00000233452 | STXBP5-AS1 | 1 | positive |
| ENSG00000233621 | LINC01137 | 1 | positive |
| ENSG00000235288 | AC099329.1 | 1 | positive |
| ENSG00000235513 | AL035681.1 | 1 | positive |
| ENSG00000237803 | LINC00211 | 1 | positive |
| ENSG00000238201 | AC114752.2 | 1 | positive |
| ENSG00000242861 | AL591895.1 | 1 | positive |
| ENSG00000244041 | LINC01011 | 1 | positive |
| ENSG00000245552 | AP000787.1 | 1 | positive |
| ENSG00000250334 | LINC00989 | 1 | positive |
| ENSG00000251301 | LINC02384 | 1 | positive |
| ENSG00000251660 | AC007036.3 | 1 | positive |
| ENSG00000253394 | LINC00534 | 1 | positive |
| ENSG00000253982 | AC100810.1 | 1 | positive |
| ENSG00000254614 | AP003068.2 | 1 | positive |
| ENSG00000255240 | AP001636.3 | 1 | positive |
| ENSG00000259172 | AC023024.1 | 1 | positive |
| ENSG00000261253 | AC137932.2 | 1 | positive |
| ENSG00000261455 | LINC01003 | 1 | positive |
| ENSG00000264964 | AP001033.1 | 1 | positive |
| ENSG00000267175 | AC105094.2 | 1 | positive |
| ENSG00000267475 | AC008736.1 | 1 | positive |
| ENSG00000269243 | AC008894.2 | 1 | positive |
| ENSG00000270028 | AC079315.1 | 1 | positive |
| ENSG00000270055 | AC127502.2 | 1 | positive |
| ENSG00000272742 | AC135457.1 | 1 | positive |
| ENSG00000275294 | LINC02340 | 1 | positive |
| ENSG00000279841 | AC092135.3 | 1 | positive |
| ENSG00000280106 | AC008555.8 | 1 | positive |
| ENSG00000280279 | AC240565.2 | 1 | positive |
| ENSG00000280739 | EIF1B-AS1 | 1 | positive |
| ENSG00000281332 | LINC00997 | 1 | positive |
| ENSG00000282828 | AC009971.1 | 1 | positive |
| ENSG00000283633 | AP000547.3 | 1 | positive |
| ENSG00000174365 | SNHG11 | 1 | negative |
| ENSG00000177410 | ZFAS1 | 1 | negative |
| ENSG00000187185 | AC092118.1 | 1 | negative |
| ENSG00000203706 | SERTAD4-AS1 | 1 | negative |
| ENSG00000214049 | UCA1 | 1 | negative |
| ENSG00000222022 | AC112721.1 | 1 | negative |
| ENSG00000222032 | AC112721.2 | 1 | negative |
| ENSG00000224490 | TTC21B-AS1 | 1 | negative |
| ENSG00000225269 | LINC00705 | 1 | negative |
| ENSG00000226856 | AC093901.1 | 1 | negative |
| ENSG00000230513 | THAP7-AS1 | 1 | negative |
| ENSG00000230663 | FAM224B | 1 | negative |
| ENSG00000231290 | APCDD1L-AS1 | 1 | negative |
| ENSG00000232490 | OSBPL10-AS1 | 1 | negative |
| ENSG00000233478 | AL031280.1 | 1 | negative |
| ENSG00000235831 | BHLHE40-AS1 | 1 | negative |
| ENSG00000235979 | AC004448.2 | 1 | negative |
| ENSG00000238160 | AC116366.2 | 1 | negative |
| ENSG00000240373 | SEC62-AS1 | 1 | negative |
| ENSG00000245975 | AC090515.2 | 1 | negative |
| ENSG00000248371 | LINC02056 | 1 | negative |
| ENSG00000251003 | ZFPM2-AS1 | 1 | negative |
| ENSG00000254531 | AP001816.1 | 1 | negative |
| ENSG00000254584 | AL035078.1 | 1 | negative |
| ENSG00000255198 | SNHG9 | 1 | negative |
| ENSG00000255327 | AP003062.1 | 1 | negative |
| ENSG00000255462 | AC104031.1 | 1 | negative |
| ENSG00000256073 | URB1-AS1 | 1 | negative |
| ENSG00000257225 | AC079601.1 | 1 | negative |
| ENSG00000258140 | AC127894.1 | 1 | negative |
| ENSG00000258512 | LINC00239 | 1 | negative |
| ENSG00000259697 | AC126323.2 | 1 | negative |
| ENSG00000260000 | AL133338.1 | 1 | negative |
| ENSG00000260179 | AL162741.1 | 1 | negative |
| ENSG00000261188 | Z95115.1 | 1 | negative |
| ENSG00000261250 | AL160286.3 | 1 | negative |
| ENSG00000265678 | AC129510.1 | 1 | negative |
| ENSG00000267493 | CIRBP-AS1 | 1 | negative |
| ENSG00000267519 | AC020916.1 | 1 | negative |
| ENSG00000268049 | AC012313.2 | 1 | negative |
| ENSG00000268621 | IGFL2-AS1 | 1 | negative |
| ENSG00000268941 | LINC01711 | 1 | negative |
| ENSG00000269053 | AC010319.3 | 1 | negative |
| ENSG00000269906 | AL606834.1 | 1 | negative |
| ENSG00000271020 | AC112220.2 | 1 | negative |
| ENSG00000271643 | AC112220.4 | 1 | negative |
| ENSG00000272144 | AC025171.4 | 1 | negative |
| ENSG00000272256 | AC044849.1 | 1 | negative |
| ENSG00000272994 | AC012360.3 | 1 | negative |
| ENSG00000273338 | AC103591.3 | 1 | negative |
| ENSG00000275765 | AC091982.3 | 1 | negative |
| ENSG00000277969 | AC006449.6 | 1 | negative |
| ENSG00000279364 | AC114546.3 | 1 | negative |
| ENSG00000279780 | AC138907.9 | 1 | negative |
| ENSG00000280120 | AC073857.1 | 1 | negative |
| ENSG00000281371 | INE2 | 1 | negative |
| ENSG00000282885 | AL627171.2 | 1 | negative |
| ENSG00000284116 | AL772307.1 | 1 | negative |
| ENSG00000284618 | AL391294.1 | 1 | negative |
| ENSG00000196951 | SCOC-AS1 | 1 | negative |
| ENSG00000228649 | SNHG26 | 1 | negative |
| ENSG00000231628 | AL133406.2 | 1 | negative |
| ENSG00000234509 | AP000253.1 | 1 | negative |
| ENSG00000236882 | LINC01554 | 1 | negative |
| ENSG00000239453 | SIDT1-AS1 | 1 | negative |
| ENSG00000240137 | ERICH6-AS1 | 1 | negative |
| ENSG00000249228 | AC068944.1 | 1 | negative |
| ENSG00000250781 | AC024022.1 | 1 | negative |
| ENSG00000251186 | AC105345.2 | 1 | negative |
| ENSG00000253138 | LINC00967 | 1 | negative |
| ENSG00000254972 | AP003498.1 | 1 | negative |
| ENSG00000257467 | PPFIA2-AS1 | 1 | negative |
| ENSG00000259221 | AC023905.1 | 1 | negative |
| ENSG00000260912 | AL158206.1 | 1 | negative |
| ENSG00000263300 | AC015853.3 | 1 | negative |
| ENSG00000267576 | AC011472.3 | 1 | negative |
| ENSG00000275367 | AC092111.1 | 1 | negative |
| ENSG00000278768 | BACE1-AS | 1 | negative |
| ENSG00000279535 | AP003476.1 | 1 | negative |
| ENSG00000157306 | ZFHX2-AS1 | 1 | negative |
| ENSG00000161149 | TUBA3FP | 1 | negative |
| ENSG00000176320 | AL138899.1 | 1 | negative |
| ENSG00000178457 | LINC00314 | 1 | negative |
| ENSG00000179253 | AL162457.1 | 1 | negative |
| ENSG00000179818 | PCBP1-AS1 | 1 | negative |
| ENSG00000181798 | LINC00471 | 1 | negative |
| ENSG00000187621 | TCL6 | 1 | negative |
| ENSG00000188825 | LINC00910 | 1 | negative |
| ENSG00000189229 | AC069277.1 | 1 | negative |
| ENSG00000196295 | AC005154.1 | 1 | negative |
| ENSG00000196810 | CTBP1-AS2 | 1 | negative |
| ENSG00000197099 | AC068631.1 | 1 | negative |
| ENSG00000197332 | AC008543.1 | 1 | negative |
| ENSG00000197880 | MDS2 | 1 | negative |
| ENSG00000198468 | FLVCR1-AS1 | 1 | negative |
| ENSG00000203280 | AL022323.1 | 1 | negative |
| ENSG00000203327 | AC012358.1 | 1 | negative |
| ENSG00000203356 | LINC01562 | 1 | negative |
| ENSG00000203386 | LINC01317 | 1 | negative |
| ENSG00000203469 | AL354956.1 | 1 | negative |
| ENSG00000203497 | PDCD4-AS1 | 1 | negative |
| ENSG00000203601 | LINC00970 | 1 | negative |
| ENSG00000203635 | AC144450.1 | 1 | negative |
| ENSG00000204054 | LINC00963 | 1 | negative |
| ENSG00000204685 | STARD7-AS1 | 1 | negative |
| ENSG00000205056 | LINC02397 | 1 | negative |
| ENSG00000205662 | FAM239C | 1 | negative |
| ENSG00000205890 | AC108134.1 | 1 | negative |
| ENSG00000205959 | AC105345.1 | 1 | negative |
| ENSG00000206567 | AC022007.1 | 1 | negative |
| ENSG00000214145 | LINC00887 | 1 | negative |
| ENSG00000214559 | AC019077.1 | 1 | negative |
| ENSG00000214719 | AC005562.1 | 1 | negative |
| ENSG00000214770 | AL161756.1 | 1 | negative |
| ENSG00000215014 | AL645728.1 | 1 | negative |
| ENSG00000215241 | LINC02449 | 1 | negative |
| ENSG00000215244 | AL137145.2 | 1 | negative |
| ENSG00000215256 | DHRS4-AS1 | 1 | negative |
| ENSG00000215866 | LINC01356 | 1 | negative |
| ENSG00000216895 | AC009403.1 | 1 | negative |
| ENSG00000223720 | AL109659.1 | 1 | negative |
| ENSG00000223770 | AC006145.1 | 1 | negative |
| ENSG00000223797 | ENTPD3-AS1 | 1 | negative |
| ENSG00000223806 | LINC00114 | 1 | negative |
| ENSG00000223949 | ROR1-AS1 | 1 | negative |
| ENSG00000223960 | AC009948.1 | 1 | negative |
| ENSG00000224078 | SNHG14 | 1 | negative |
| ENSG00000224099 | AC104823.1 | 1 | negative |
| ENSG00000224165 | DNAJC27-AS1 | 1 | negative |
| ENSG00000224238 | WARS2-IT1 | 1 | negative |
| ENSG00000224358 | AL451074.2 | 1 | negative |
| ENSG00000224424 | PRKAR2A-AS1 | 1 | negative |
| ENSG00000224592 | AL139158.2 | 1 | negative |
| ENSG00000224799 | AL139397.1 | 1 | negative |
| ENSG00000224810 | AL355482.1 | 1 | negative |
| ENSG00000224822 | THRB-IT1 | 1 | negative |
| ENSG00000225194 | LINC00092 | 1 | negative |
| ENSG00000225313 | AL513327.1 | 1 | negative |
| ENSG00000225321 | LINC01427 | 1 | negative |
| ENSG00000225470 | JPX | 1 | negative |
| ENSG00000225546 | LINC02476 | 1 | negative |
| ENSG00000225963 | AC009950.1 | 1 | negative |
| ENSG00000226017 | PRICKLE2-AS3 | 1 | negative |
| ENSG00000226043 | AP000561.1 | 1 | negative |
| ENSG00000226053 | LINC01776 | 1 | negative |
| ENSG00000226091 | LINC00937 | 1 | negative |
| ENSG00000226125 | LINC01907 | 1 | negative |
| ENSG00000226235 | LEMD1-AS1 | 1 | negative |
| ENSG00000226328 | NUP50-AS1 | 1 | negative |
| ENSG00000226419 | SLC16A1-AS1 | 1 | negative |
| ENSG00000226674 | TEX41 | 1 | negative |
| ENSG00000226688 | ENTPD1-AS1 | 1 | negative |
| ENSG00000226891 | LINC01359 | 1 | negative |
| ENSG00000227028 | SLC8A1-AS1 | 1 | negative |
| ENSG00000227373 | AL121983.2 | 1 | negative |
| ENSG00000227695 | DNMBP-AS1 | 1 | negative |
| ENSG00000227733 | AC239809.3 | 1 | negative |
| ENSG00000227740 | AL513329.1 | 1 | negative |
| ENSG00000227963 | RBM15-AS1 | 1 | negative |
| ENSG00000228005 | AC020743.1 | 1 | negative |
| ENSG00000228113 | AC003991.1 | 1 | negative |
| ENSG00000228274 | AL021707.2 | 1 | negative |
| ENSG00000228434 | AC004951.1 | 1 | negative |
| ENSG00000228543 | AC003684.1 | 1 | negative |
| ENSG00000228689 | AL355997.1 | 1 | negative |
| ENSG00000228697 | AL023755.1 | 1 | negative |
| ENSG00000228824 | MIR4500HG | 1 | negative |
| ENSG00000228862 | AC068389.1 | 1 | negative |
| ENSG00000228918 | LINC01344 | 1 | negative |
| ENSG00000228980 | LINC01205 | 1 | negative |
| ENSG00000229140 | CCDC26 | 1 | negative |
| ENSG00000229267 | AC016708.1 | 1 | negative |
| ENSG00000229491 | AC136489.1 | 1 | negative |
| ENSG00000229498 | AC105053.1 | 1 | negative |
| ENSG00000229557 | LINC00379 | 1 | negative |
| ENSG00000229582 | AL358074.1 | 1 | negative |
| ENSG00000229587 | AL158825.2 | 1 | negative |
| ENSG00000229589 | ACVR2B-AS1 | 1 | negative |
| ENSG00000229999 | AL022238.2 | 1 | negative |
| ENSG00000230115 | TPRG1-AS2 | 1 | negative |
| ENSG00000230415 | LINC01786 | 1 | negative |
| ENSG00000230492 | AL049651.1 | 1 | negative |
| ENSG00000230606 | AC092683.1 | 1 | negative |
| ENSG00000230612 | AC004039.1 | 1 | negative |
| ENSG00000230866 | LINC02558 | 1 | negative |
| ENSG00000231189 | AC013448.1 | 1 | negative |
| ENSG00000231252 | AC099792.1 | 1 | negative |
| ENSG00000231422 | LINC01516 | 1 | negative |
| ENSG00000231426 | FILNC1 | 1 | negative |
| ENSG00000231605 | LINC01363 | 1 | negative |
| ENSG00000231690 | LINC00574 | 1 | negative |
| ENSG00000231742 | LINC01273 | 1 | negative |
| ENSG00000231764 | DLX6-AS1 | 1 | negative |
| ENSG00000231868 | AL031848.1 | 1 | negative |
| ENSG00000231890 | DARS-AS1 | 1 | negative |
| ENSG00000231953 | AL031432.1 | 1 | negative |
| ENSG00000232063 | AL691447.2 | 1 | negative |
| ENSG00000232079 | LINC01697 | 1 | negative |
| ENSG00000232190 | LINC02181 | 1 | negative |
| ENSG00000232225 | LINC01047 | 1 | negative |
| ENSG00000232353 | AC026320.1 | 1 | negative |
| ENSG00000232611 | AL683813.1 | 1 | negative |
| ENSG00000232677 | LINC00665 | 1 | negative |
| ENSG00000232748 | AC135050.1 | 1 | negative |
| ENSG00000232774 | AL355916.1 | 1 | negative |
| ENSG00000233048 | LINC01722 | 1 | negative |
| ENSG00000233061 | TTLL7-IT1 | 1 | negative |
| ENSG00000233251 | AC007743.1 | 1 | negative |
| ENSG00000233379 | AL139002.1 | 1 | negative |
| ENSG00000233521 | LINC01638 | 1 | negative |
| ENSG00000233672 | RNASEH2B-AS1 | 1 | negative |
| ENSG00000233871 | DLG5-AS1 | 1 | negative |
| ENSG00000234222 | LIX1L-AS1 | 1 | negative |
| ENSG00000234350 | AC007405.1 | 1 | negative |
| ENSG00000234432 | AC092171.3 | 1 | negative |
| ENSG00000234553 | AC022431.1 | 1 | negative |
| ENSG00000234640 | AL390763.1 | 1 | negative |
| ENSG00000234899 | SOX9-AS1 | 1 | negative |
| ENSG00000234913 | AC016027.2 | 1 | negative |
| ENSG00000234945 | GTF3C2-AS1 | 1 | negative |
| ENSG00000235397 | EPN2-AS1 | 1 | negative |
| ENSG00000235493 | LINC01967 | 1 | negative |
| ENSG00000235523 | AL135924.2 | 1 | negative |
| ENSG00000235652 | AL356599.1 | 1 | negative |
| ENSG00000235706 | DICER1-AS1 | 1 | negative |
| ENSG00000235823 | OLMALINC | 1 | negative |
| ENSG00000236008 | LINC01814 | 1 | negative |
| ENSG00000236532 | LINC01695 | 1 | negative |
| ENSG00000236830 | CBR3-AS1 | 1 | negative |
| ENSG00000237125 | HAND2-AS1 | 1 | negative |
| ENSG00000237352 | LINC01358 | 1 | negative |
| ENSG00000237491 | AL669831.5 | 1 | negative |
| ENSG00000237505 | PKN2-AS1 | 1 | negative |
| ENSG00000238122 | AL359258.1 | 1 | negative |
| ENSG00000238198 | AL357055.3 | 1 | negative |
| ENSG00000238755 | LINC02006 | 1 | negative |
| ENSG00000240401 | AC012358.3 | 1 | negative |
| ENSG00000240405 | SAMMSON | 1 | negative |
| ENSG00000240499 | AC004594.1 | 1 | negative |
| ENSG00000241168 | AC128685.1 | 1 | negative |
| ENSG00000241288 | AC092902.2 | 1 | negative |
| ENSG00000241472 | PTPRG-AS1 | 1 | negative |
| ENSG00000241956 | AC109466.1 | 1 | negative |
| ENSG00000242086 | MUC20-OT1 | 1 | negative |
| ENSG00000243349 | AL358394.2 | 1 | negative |
| ENSG00000244040 | IL12A-AS1 | 1 | negative |
| ENSG00000244479 | OR2A1-AS1 | 1 | negative |
| ENSG00000244675 | AC108676.1 | 1 | negative |
| ENSG00000245017 | LINC02453 | 1 | negative |
| ENSG00000245025 | AC107959.1 | 1 | negative |
| ENSG00000245156 | AP001107.1 | 1 | negative |
| ENSG00000245750 | DRAIC | 1 | negative |
| ENSG00000245937 | LINC01184 | 1 | negative |
| ENSG00000245970 | AP003352.1 | 1 | negative |
| ENSG00000246174 | KCTD21-AS1 | 1 | negative |
| ENSG00000246223 | LINC01550 | 1 | negative |
| ENSG00000246273 | SBF2-AS1 | 1 | negative |
| ENSG00000246339 | EXTL3-AS1 | 1 | negative |
| ENSG00000246366 | LACTB2-AS1 | 1 | negative |
| ENSG00000246465 | AC138904.1 | 1 | negative |
| ENSG00000246740 | PLA2G4E-AS1 | 1 | negative |
| ENSG00000247130 | AC138781.1 | 1 | negative |
| ENSG00000247317 | LY6E-DT | 1 | negative |
| ENSG00000247556 | OIP5-AS1 | 1 | negative |
| ENSG00000247572 | CKMT2-AS1 | 1 | negative |
| ENSG00000247809 | NR2F2-AS1 | 1 | negative |
| ENSG00000247934 | AC022364.1 | 1 | negative |
| ENSG00000248049 | UBA6-AS1 | 1 | negative |
| ENSG00000248360 | LINC00504 | 1 | negative |
| ENSG00000248458 | AL139147.1 | 1 | negative |
| ENSG00000248874 | C5orf17 | 1 | negative |
| ENSG00000249125 | AC093821.1 | 1 | negative |
| ENSG00000249348 | UGDH-AS1 | 1 | negative |
| ENSG00000249604 | AC096564.2 | 1 | negative |
| ENSG00000249614 | LINC02503 | 1 | negative |
| ENSG00000249667 | LINC01259 | 1 | negative |
| ENSG00000249699 | LINC02261 | 1 | negative |
| ENSG00000249700 | SRD5A3-AS1 | 1 | negative |
| ENSG00000249731 | AC126768.3 | 1 | negative |
| ENSG00000249859 | PVT1 | 1 | negative |
| ENSG00000250303 | AP002884.1 | 1 | negative |
| ENSG00000250387 | LINC02197 | 1 | negative |
| ENSG00000250472 | TRIM36-IT1 | 1 | negative |
| ENSG00000250501 | AC093766.1 | 1 | negative |
| ENSG00000250685 | AC009123.1 | 1 | negative |
| ENSG00000250790 | AC127070.2 | 1 | negative |
| ENSG00000250802 | ZBED3-AS1 | 1 | negative |
| ENSG00000250831 | AC074131.1 | 1 | negative |
| ENSG00000250865 | AC105362.1 | 1 | negative |
| ENSG00000250903 | GMDS-AS1 | 1 | negative |
| ENSG00000251152 | AC025539.1 | 1 | negative |
| ENSG00000251187 | AC010261.2 | 1 | negative |
| ENSG00000251298 | AC093835.1 | 1 | negative |
| ENSG00000251330 | AC114939.1 | 1 | negative |
| ENSG00000251381 | LINC00958 | 1 | negative |
| ENSG00000251396 | LINC01301 | 1 | negative |
| ENSG00000251504 | LINC01099 | 1 | negative |
| ENSG00000251527 | AC097110.1 | 1 | negative |
| ENSG00000251602 | AL928654.1 | 1 | negative |
| ENSG00000251637 | AP003716.1 | 1 | negative |
| ENSG00000253390 | AC104561.1 | 1 | negative |
| ENSG00000253438 | PCAT1 | 1 | negative |
| ENSG00000253642 | AF279873.3 | 1 | negative |
| ENSG00000253686 | LINC01484 | 1 | negative |
| ENSG00000253712 | AC022274.1 | 1 | negative |
| ENSG00000253799 | LINC01030 | 1 | negative |
| ENSG00000254153 | AC103957.2 | 1 | negative |
| ENSG00000254166 | CASC19 | 1 | negative |
| ENSG00000254187 | AC008708.2 | 1 | negative |
| ENSG00000254202 | AC015522.1 | 1 | negative |
| ENSG00000254251 | AC103770.1 | 1 | negative |
| ENSG00000254275 | LINC00824 | 1 | negative |
| ENSG00000254287 | AC007991.4 | 1 | negative |
| ENSG00000254337 | AC083967.1 | 1 | negative |
| ENSG00000254488 | AC007876.1 | 1 | negative |
| ENSG00000254495 | AP000487.2 | 1 | negative |
| ENSG00000254587 | AP003066.1 | 1 | negative |
| ENSG00000254635 | WAC-AS1 | 1 | negative |
| ENSG00000254790 | AP000842.1 | 1 | negative |
| ENSG00000254802 | AC022182.2 | 1 | negative |
| ENSG00000254973 | AC105219.4 | 1 | negative |
| ENSG00000254990 | AP001781.1 | 1 | negative |
| ENSG00000255165 | AC134775.1 | 1 | negative |
| ENSG00000255289 | AC068389.3 | 1 | negative |
| ENSG00000255468 | AP001107.9 | 1 | negative |
| ENSG00000256571 | AC079866.2 | 1 | negative |
| ENSG00000257120 | AL356756.1 | 1 | negative |
| ENSG00000257337 | AC068888.1 | 1 | negative |
| ENSG00000257398 | AC126177.4 | 1 | negative |
| ENSG00000257475 | AC068888.2 | 1 | negative |
| ENSG00000257636 | G2E3-AS1 | 1 | negative |
| ENSG00000257640 | AC096558.2 | 1 | negative |
| ENSG00000257870 | AC027287.2 | 1 | negative |
| ENSG00000257894 | AC027288.3 | 1 | negative |
| ENSG00000258038 | LINC02327 | 1 | negative |
| ENSG00000258178 | AC016993.1 | 1 | negative |
| ENSG00000258711 | AL358334.2 | 1 | negative |
| ENSG00000258779 | LINC01568 | 1 | negative |
| ENSG00000258792 | AL137230.1 | 1 | negative |
| ENSG00000258807 | AL359237.1 | 1 | negative |
| ENSG00000258844 | AL162511.1 | 1 | negative |
| ENSG00000258851 | AL139300.2 | 1 | negative |
| ENSG00000258955 | LINC00519 | 1 | negative |
| ENSG00000258985 | AL352979.2 | 1 | negative |
| ENSG00000259219 | AC084855.2 | 1 | negative |
| ENSG00000259334 | LINC00596 | 1 | negative |
| ENSG00000259345 | AC013652.1 | 1 | negative |
| ENSG00000259361 | LINC00927 | 1 | negative |
| ENSG00000259380 | AC087473.1 | 1 | negative |
| ENSG00000259402 | AC090515.5 | 1 | negative |
| ENSG00000259495 | AC016705.2 | 1 | negative |
| ENSG00000259654 | AC115102.1 | 1 | negative |
| ENSG00000259690 | AC107980.1 | 1 | negative |
| ENSG00000259736 | CRTC3-AS1 | 1 | negative |
| ENSG00000259744 | AC009269.4 | 1 | negative |
| ENSG00000259776 | AC093426.1 | 1 | negative |
| ENSG00000259786 | LINC02109 | 1 | negative |
| ENSG00000259793 | AC013726.1 | 1 | negative |
| ENSG00000259810 | AC002519.1 | 1 | negative |
| ENSG00000259891 | AC107375.1 | 1 | negative |
| ENSG00000259895 | AC106820.2 | 1 | negative |
| ENSG00000259921 | AC022819.1 | 1 | negative |
| ENSG00000259994 | AL353796.1 | 1 | negative |
| ENSG00000260072 | AC008938.1 | 1 | negative |
| ENSG00000260084 | AC126773.1 | 1 | negative |
| ENSG00000260233 | SSSCA1-AS1 | 1 | negative |
| ENSG00000260388 | LINC00562 | 1 | negative |
| ENSG00000260409 | AC012414.5 | 1 | negative |
| ENSG00000260507 | AC133919.1 | 1 | negative |
| ENSG00000260778 | AC009065.4 | 1 | negative |
| ENSG00000260814 | AC073657.1 | 1 | negative |
| ENSG00000260852 | FBXL19-AS1 | 1 | negative |
| ENSG00000260913 | LINC01254 | 1 | negative |
| ENSG00000260948 | AL390195.2 | 1 | negative |
| ENSG00000260975 | AC007333.2 | 1 | negative |
| ENSG00000261068 | AL512274.1 | 1 | negative |
| ENSG00000261327 | AC134312.5 | 1 | negative |
| ENSG00000261392 | AC087190.2 | 1 | negative |
| ENSG00000261404 | AC138627.1 | 1 | negative |
| ENSG00000261408 | TEN1-CDK3 | 1 | negative |
| ENSG00000261420 | AL022069.1 | 1 | negative |
| ENSG00000261501 | AC079341.2 | 1 | negative |
| ENSG00000261654 | AL360270.2 | 1 | negative |
| ENSG00000261663 | AC009065.8 | 1 | negative |
| ENSG00000261736 | AC002551.1 | 1 | negative |
| ENSG00000261801 | LOXL1-AS1 | 1 | negative |
| ENSG00000262039 | AC091180.5 | 1 | negative |
| ENSG00000262888 | AC005736.2 | 1 | negative |
| ENSG00000263072 | ZNF213-AS1 | 1 | negative |
| ENSG00000263234 | AC010401.2 | 1 | negative |
| ENSG00000263494 | AC004702.1 | 1 | negative |
| ENSG00000263731 | AC145207.5 | 1 | negative |
| ENSG00000263924 | AC022960.1 | 1 | negative |
| ENSG00000264019 | AC018521.2 | 1 | negative |
| ENSG00000264067 | AC005291.1 | 1 | negative |
| ENSG00000264083 | AC005899.1 | 1 | negative |
| ENSG00000264112 | AC015813.1 | 1 | negative |
| ENSG00000264546 | AC008026.3 | 1 | negative |
| ENSG00000264587 | AC117569.1 | 1 | negative |
| ENSG00000264707 | L3MBTL4-AS1 | 1 | negative |
| ENSG00000265992 | ESRG | 1 | negative |
| ENSG00000266171 | AP001020.3 | 1 | negative |
| ENSG00000266290 | AC015813.3 | 1 | negative |
| ENSG00000266340 | AC138207.7 | 1 | negative |
| ENSG00000266896 | AL354892.3 | 1 | negative |
| ENSG00000266990 | AC004528.1 | 1 | negative |
| ENSG00000267044 | AC005757.1 | 1 | negative |
| ENSG00000267065 | LINC02080 | 1 | negative |
| ENSG00000267080 | ASB16-AS1 | 1 | negative |
| ENSG00000267100 | ILF3-AS1 | 1 | negative |
| ENSG00000267239 | AP001198.1 | 1 | negative |
| ENSG00000267254 | AC020928.1 | 1 | negative |
| ENSG00000267423 | AC005616.1 | 1 | negative |
| ENSG00000267452 | LINC02073 | 1 | negative |
| ENSG00000267462 | AC090377.1 | 1 | negative |
| ENSG00000267470 | ZNF571-AS1 | 1 | negative |
| ENSG00000267629 | AC138430.1 | 1 | negative |
| ENSG00000267659 | LINC01482 | 1 | negative |
| ENSG00000267672 | AC010632.2 | 1 | negative |
| ENSG00000267749 | AC092068.3 | 1 | negative |
| ENSG00000267750 | RUNDC3A-AS1 | 1 | negative |
| ENSG00000267872 | AC073539.1 | 1 | negative |
| ENSG00000267934 | AC010300.1 | 1 | negative |
| ENSG00000267939 | AC008946.1 | 1 | negative |
| ENSG00000268119 | AC010615.2 | 1 | negative |
| ENSG00000268205 | AC005261.1 | 1 | negative |
| ENSG00000268635 | AP003680.1 | 1 | negative |
| ENSG00000268658 | LINC00664 | 1 | negative |
| ENSG00000268746 | AC010519.1 | 1 | negative |
| ENSG00000268996 | MAN1B1-AS1 | 1 | negative |
| ENSG00000269303 | AC020907.3 | 1 | negative |
| ENSG00000269486 | ERVK9-11 | 1 | negative |
| ENSG00000269514 | AC024257.3 | 1 | negative |
| ENSG00000269694 | AC005197.1 | 1 | negative |
| ENSG00000269752 | AC008761.2 | 1 | negative |
| ENSG00000269793 | ZIM2-AS1 | 1 | negative |
| ENSG00000269821 | KCNQ1OT1 | 1 | negative |
| ENSG00000269825 | AC022150.4 | 1 | negative |
| ENSG00000269895 | AP000654.1 | 1 | negative |
| ENSG00000270641 | TSIX | 1 | negative |
| ENSG00000270871 | AC015849.3 | 1 | negative |
| ENSG00000270959 | LPP-AS2 | 1 | negative |
| ENSG00000270996 | AC005034.4 | 1 | negative |
| ENSG00000271122 | AC018647.2 | 1 | negative |
| ENSG00000271420 | AL109936.2 | 1 | negative |
| ENSG00000271851 | AC087501.4 | 1 | negative |
| ENSG00000271913 | AL035530.2 | 1 | negative |
| ENSG00000272027 | AC073218.2 | 1 | negative |
| ENSG00000272054 | AC007390.2 | 1 | negative |
| ENSG00000272455 | AL391244.3 | 1 | negative |
| ENSG00000272574 | AL596325.2 | 1 | negative |
| ENSG00000272754 | AL133245.1 | 1 | negative |
| ENSG00000272909 | AL122035.2 | 1 | negative |
| ENSG00000272989 | LINC02012 | 1 | negative |
| ENSG00000273018 | AC107983.2 | 1 | negative |
| ENSG00000273218 | AC005776.2 | 1 | negative |
| ENSG00000273247 | AC097376.2 | 1 | negative |
| ENSG00000273270 | AC090114.2 | 1 | negative |
| ENSG00000274023 | AL360169.2 | 1 | negative |
| ENSG00000274220 | AC009163.7 | 1 | negative |
| ENSG00000274281 | AC022929.2 | 1 | negative |
| ENSG00000275512 | AC007998.4 | 1 | negative |
| ENSG00000275678 | AL133320.1 | 1 | negative |
| ENSG00000275897 | AC021491.4 | 1 | negative |
| ENSG00000276007 | AC079414.3 | 1 | negative |
| ENSG00000276250 | AC127024.6 | 1 | negative |
| ENSG00000276337 | AC105429.1 | 1 | negative |
| ENSG00000277128 | AL589743.5 | 1 | negative |
| ENSG00000277200 | AC005696.4 | 1 | negative |
| ENSG00000277287 | AL109976.1 | 1 | negative |
| ENSG00000277350 | AL353770.4 | 1 | negative |
| ENSG00000277449 | CEBPB-AS1 | 1 | negative |
| ENSG00000277501 | AC243571.2 | 1 | negative |
| ENSG00000277715 | AC079174.2 | 1 | negative |
| ENSG00000277938 | AL035252.3 | 1 | negative |
| ENSG00000278291 | AL161772.1 | 1 | negative |
| ENSG00000278514 | AC068831.6 | 1 | negative |
| ENSG00000278703 | AC100847.1 | 1 | negative |
| ENSG00000278949 | AC127070.4 | 1 | negative |
| ENSG00000278985 | AC092718.7 | 1 | negative |
| ENSG00000278986 | AC091060.1 | 1 | negative |
| ENSG00000279061 | Z98043.1 | 1 | negative |
| ENSG00000279066 | HEXDC-IT1 | 1 | negative |
| ENSG00000279080 | AL022322.2 | 1 | negative |
| ENSG00000279085 | AL022323.3 | 1 | negative |
| ENSG00000279110 | AL022323.4 | 1 | negative |
| ENSG00000279134 | AC090643.1 | 1 | negative |
| ENSG00000279154 | AL353726.2 | 1 | negative |
| ENSG00000279159 | AC003681.1 | 1 | negative |
| ENSG00000279200 | AC020558.3 | 1 | negative |
| ENSG00000279217 | Z95114.1 | 1 | negative |
| ENSG00000279240 | AC063980.1 | 1 | negative |
| ENSG00000279259 | AC087741.3 | 1 | negative |
| ENSG00000279281 | AC015883.1 | 1 | negative |
| ENSG00000279337 | AC127024.7 | 1 | negative |
| ENSG00000279394 | AC015871.4 | 1 | negative |
| ENSG00000279406 | AL359183.1 | 1 | negative |
| ENSG00000279417 | AC019322.4 | 1 | negative |
| ENSG00000279443 | AL513497.1 | 1 | negative |
| ENSG00000279462 | AC093028.1 | 1 | negative |
| ENSG00000279481 | AC104791.2 | 1 | negative |
| ENSG00000279520 | AC093525.8 | 1 | negative |
| ENSG00000279525 | AC002451.2 | 1 | negative |
| ENSG00000279548 | AL022323.5 | 1 | negative |
| ENSG00000279549 | AP000437.1 | 1 | negative |
| ENSG00000279561 | AL845472.2 | 1 | negative |
| ENSG00000279567 | AC005703.5 | 1 | negative |
| ENSG00000279573 | AC134407.2 | 1 | negative |
| ENSG00000279598 | AC009948.5 | 1 | negative |
| ENSG00000279648 | AP000432.2 | 1 | negative |
| ENSG00000279662 | AC131649.2 | 1 | negative |
| ENSG00000279663 | AC104623.2 | 1 | negative |
| ENSG00000279705 | AC092701.1 | 1 | negative |
| ENSG00000279706 | AL353608.4 | 1 | negative |
| ENSG00000279721 | AC018737.2 | 1 | negative |
| ENSG00000279759 | AC118344.2 | 1 | negative |
| ENSG00000279821 | AC145098.2 | 1 | negative |
| ENSG00000279840 | AC004801.7 | 1 | negative |
| ENSG00000279879 | AC091152.4 | 1 | negative |
| ENSG00000279912 | AC068448.1 | 1 | negative |
| ENSG00000279960 | AL137783.1 | 1 | negative |
| ENSG00000280025 | AL008638.6 | 1 | negative |
| ENSG00000280058 | AL117378.2 | 1 | negative |
| ENSG00000280063 | AC012676.5 | 1 | negative |
| ENSG00000280064 | AC130304.1 | 1 | negative |
| ENSG00000280069 | AC127024.8 | 1 | negative |
| ENSG00000280087 | AC011481.3 | 1 | negative |
| ENSG00000280088 | AC126474.2 | 1 | negative |
| ENSG00000280105 | AC009487.4 | 1 | negative |
| ENSG00000280131 | AC027348.2 | 1 | negative |
| ENSG00000280176 | AC012486.1 | 1 | negative |
| ENSG00000280187 | AC022107.1 | 1 | negative |
| ENSG00000280213 | UCKL1-AS1 | 1 | negative |
| ENSG00000280214 | AC027682.7 | 1 | negative |
| ENSG00000280242 | AL450226.2 | 1 | negative |
| ENSG00000280248 | AC124319.4 | 1 | negative |
| ENSG00000280291 | AC016251.2 | 1 | negative |
| ENSG00000280340 | AC080080.2 | 1 | negative |
| ENSG00000280345 | AC092184.1 | 1 | negative |
| ENSG00000280377 | AC027176.3 | 1 | negative |
| ENSG00000280384 | FP325332.1 | 1 | negative |
| ENSG00000280395 | AL034546.1 | 1 | negative |
| ENSG00000280396 | AC114546.4 | 1 | negative |
| ENSG00000280474 | AL356481.2 | 1 | negative |
| ENSG00000280623 | PCAT14 | 1 | negative |
| ENSG00000280665 | AL513210.1 | 1 | negative |
| ENSG00000280711 | JADRR | 1 | negative |
| ENSG00000280798 | LINC00294 | 1 | negative |
| ENSG00000281189 | GHET1 | 1 | negative |
| ENSG00000281344 | HELLPAR | 1 | negative |
| ENSG00000281392 | LINC00506 | 1 | negative |
| ENSG00000281501 | SEPSECS-AS1 | 1 | negative |
| ENSG00000281706 | LINC01012 | 1 | negative |
| ENSG00000281731 | AC110079.2 | 1 | negative |
| ENSG00000281852 | LINC00891 | 1 | negative |
| ENSG00000282602 | Z82244.2 | 1 | negative |
| ENSG00000282772 | AL358790.1 | 1 | negative |
| ENSG00000282851 | BISPR | 1 | negative |
| ENSG00000283103 | AC010642.2 | 1 | negative |
| ENSG00000283422 | LINC02452 | 1 | negative |
| ENSG00000283608 | AL031056.2 | 1 | negative |
| ENSG00000283662 | AC138904.3 | 1 | negative |
| ENSG00000283674 | AC068587.4 | 1 | negative |
| ENSG00000283897 | AC011416.3 | 1 | negative |
| ENSG00000284428 | AC092329.4 | 1 | negative |
| ENSG00000284523 | AC004834.1 | 1 | negative |
| ENSG00000284543 | LINC01226 | 1 | negative |
| ENSG00000284612 | AL591543.1 | 1 | negative |
| ENSG00000284624 | AC092902.5 | 1 | negative |
| ENSG00000284726 | AL109936.6 | 1 | negative |
| ENSG00000284735 | AL139424.3 | 1 | negative |
| ENSG00000284968 | AC093827.4 | 1 | negative |
| ENSG00000285367 | AC087564.1 | 1 | negative |
| ENSG00000285486 | AC003043.2 | 1 | negative |
