# Supplementary material for: Development of an Inflammation-Related lncRNA-miRNA-mRNA Network Based on Competing Endogenous RNA in Breast Cancer at Single-Cell Resolution
Source: Front Cell Dev Biol. 2022 Jan 25;10:839876. doi: 10.3389/fcell.2022.839876 (PMC8821924; doi:10.3389/fcell.2022.839876)
Supplement: Supplementary file 2 [file Table2.DOCX]

**Table S2**. The genes associated with inflammation in breast cancer obtained from cancerSEA database.

| Ensemble ID | Symbol | No. dataset | Direction |
| --- | --- | --- | --- |
| ENSG00000034152 | MAP2K3 | 3 | positive |
| ENSG00000137801 | THBS1 | 3 | positive |
| ENSG00000169429 | CXCL8 | 3 | positive |
| ENSG00000204525 | HLA-C | 3 | positive |
| ENSG00000206503 | HLA-A | 3 | positive |
| ENSG00000234745 | HLA-B | 3 | positive |
| ENSG00000240065 | PSMB9 | 3 | positive |
| ENSG00000136888 | ATP6V1G1 | 3 | negative |
| ENSG00000137154 | RPS6 | 3 | negative |
| ENSG00000008517 | IL32 | 2 | positive |
| ENSG00000015475 | BID | 2 | positive |
| ENSG00000019582 | CD74 | 2 | positive |
| ENSG00000035403 | VCL | 2 | positive |
| ENSG00000058085 | LAMC2 | 2 | positive |
| ENSG00000070190 | DAPP1 | 2 | positive |
| ENSG00000072110 | ACTN1 | 2 | positive |
| ENSG00000081041 | CXCL2 | 2 | positive |
| ENSG00000087074 | PPP1R15A | 2 | positive |
| ENSG00000100906 | NFKBIA | 2 | positive |
| ENSG00000103187 | COTL1 | 2 | positive |
| ENSG00000106366 | SERPINE1 | 2 | positive |
| ENSG00000107438 | PDLIM1 | 2 | positive |
| ENSG00000108622 | ICAM2 | 2 | positive |
| ENSG00000110047 | EHD1 | 2 | positive |
| ENSG00000115009 | CCL20 | 2 | positive |
| ENSG00000118508 | RAB32 | 2 | positive |
| ENSG00000122786 | CALD1 | 2 | positive |
| ENSG00000123240 | OPTN | 2 | positive |
| ENSG00000125347 | IRF1 | 2 | positive |
| ENSG00000127314 | RAP1B | 2 | positive |
| ENSG00000133639 | BTG1 | 2 | positive |
| ENSG00000137331 | IER3 | 2 | positive |
| ENSG00000139289 | PHLDA1 | 2 | positive |
| ENSG00000149357 | LAMTOR1 | 2 | positive |
| ENSG00000159840 | ZYX | 2 | positive |
| ENSG00000161011 | SQSTM1 | 2 | positive |
| ENSG00000163739 | CXCL1 | 2 | positive |
| ENSG00000165949 | IFI27 | 2 | positive |
| ENSG00000166710 | B2M | 2 | positive |
| ENSG00000168394 | TAP1 | 2 | positive |
| ENSG00000172164 | SNTB1 | 2 | positive |
| ENSG00000175602 | CCDC85B | 2 | positive |
| ENSG00000176170 | SPHK1 | 2 | positive |
| ENSG00000181467 | RAP2B | 2 | positive |
| ENSG00000185022 | MAFF | 2 | positive |
| ENSG00000196735 | HLA-DQA1 | 2 | positive |
| ENSG00000196776 | CD47 | 2 | positive |
| ENSG00000204592 | HLA-E | 2 | positive |
| ENSG00000204642 | HLA-F | 2 | positive |
| ENSG00000232810 | TNF | 2 | positive |
| ENSG00000271503 | CCL5 | 2 | positive |
| ENSG00000008394 | MGST1 | 2 | negative |
| ENSG00000034510 | TMSB10 | 2 | negative |
| ENSG00000068697 | LAPTM4A | 2 | negative |
| ENSG00000074696 | HACD3 | 2 | negative |
| ENSG00000084234 | APLP2 | 2 | negative |
| ENSG00000089220 | PEBP1 | 2 | negative |
| ENSG00000090263 | MRPS33 | 2 | negative |
| ENSG00000090581 | GNPTG | 2 | negative |
| ENSG00000091436 | MAP3K20 | 2 | negative |
| ENSG00000100519 | PSMC6 | 2 | negative |
| ENSG00000101849 | TBL1X | 2 | negative |
| ENSG00000103363 | ELOB | 2 | negative |
| ENSG00000104870 | FCGRT | 2 | negative |
| ENSG00000105514 | RAB3D | 2 | negative |
| ENSG00000105640 | RPL18A | 2 | negative |
| ENSG00000108423 | TUBD1 | 2 | negative |
| ENSG00000108848 | LUC7L3 | 2 | negative |
| ENSG00000108953 | YWHAE | 2 | negative |
| ENSG00000109971 | HSPA8 | 2 | negative |
| ENSG00000111696 | NT5DC3 | 2 | negative |
| ENSG00000111785 | RIC8B | 2 | negative |
| ENSG00000115677 | HDLBP | 2 | negative |
| ENSG00000116005 | PCYOX1 | 2 | negative |
| ENSG00000116586 | LAMTOR2 | 2 | negative |
| ENSG00000125743 | SNRPD2 | 2 | negative |
| ENSG00000126261 | UBA2 | 2 | negative |
| ENSG00000127616 | SMARCA4 | 2 | negative |
| ENSG00000128050 | PAICS | 2 | negative |
| ENSG00000131051 | RBM39 | 2 | negative |
| ENSG00000131503 | ANKHD1 | 2 | negative |
| ENSG00000134375 | TIMM17A | 2 | negative |
| ENSG00000136810 | TXN | 2 | negative |
| ENSG00000136942 | RPL35 | 2 | negative |
| ENSG00000138674 | SEC31A | 2 | negative |
| ENSG00000140575 | IQGAP1 | 2 | negative |
| ENSG00000140990 | NDUFB10 | 2 | negative |
| ENSG00000142937 | RPS8 | 2 | negative |
| ENSG00000143127 | ITGA10 | 2 | negative |
| ENSG00000146223 | RPL7L1 | 2 | negative |
| ENSG00000146733 | PSPH | 2 | negative |
| ENSG00000146842 | TMEM209 | 2 | negative |
| ENSG00000147274 | RBMX | 2 | negative |
| ENSG00000147604 | RPL7 | 2 | negative |
| ENSG00000148303 | RPL7A | 2 | negative |
| ENSG00000149182 | ARFGAP2 | 2 | negative |
| ENSG00000154723 | ATP5PF | 2 | negative |
| ENSG00000155876 | RRAGA | 2 | negative |
| ENSG00000159140 | SON | 2 | negative |
| ENSG00000162244 | RPL29 | 2 | negative |
| ENSG00000163683 | SMIM14 | 2 | negative |
| ENSG00000168092 | PAFAH1B2 | 2 | negative |
| ENSG00000168101 | NUDT16L1 | 2 | negative |
| ENSG00000168214 | RBPJ | 2 | negative |
| ENSG00000170412 | GPRC5C | 2 | negative |
| ENSG00000170421 | KRT8 | 2 | negative |
| ENSG00000171105 | INSR | 2 | negative |
| ENSG00000173436 | MINOS1 | 2 | negative |
| ENSG00000173905 | GOLIM4 | 2 | negative |
| ENSG00000175756 | AURKAIP1 | 2 | negative |
| ENSG00000176834 | VSIG10 | 2 | negative |
| ENSG00000178814 | OPLAH | 2 | negative |
| ENSG00000182670 | TTC3 | 2 | negative |
| ENSG00000183287 | CCBE1 | 2 | negative |
| ENSG00000183691 | NOG | 2 | negative |
| ENSG00000196712 | NF1 | 2 | negative |
| ENSG00000196873 | CBWD3 | 2 | negative |
| ENSG00000198015 | MRPL42 | 2 | negative |
| ENSG00000213965 | NUDT19 | 2 | negative |
| ENSG00000135390 | ATP5MC2 | 2 | negative |
| ENSG00000169230 | PRELID1 | 2 | negative |
| ENSG00000205542 | TMSB4X | 2 | positive |
| ENSG00000223865 | HLA-DPB1 | 2 | positive |
| ENSG00000231925 | TAPBP | 2 | positive |
| ENSG00000002549 | LAP3 | 1 | positive |
| ENSG00000005249 | PRKAR2B | 1 | positive |
| ENSG00000006118 | TMEM132A | 1 | positive |
| ENSG00000006210 | CX3CL1 | 1 | positive |
| ENSG00000006638 | TBXA2R | 1 | positive |
| ENSG00000006747 | SCIN | 1 | positive |
| ENSG00000010810 | FYN | 1 | positive |
| ENSG00000011132 | APBA3 | 1 | positive |
| ENSG00000011422 | PLAUR | 1 | positive |
| ENSG00000013016 | EHD3 | 1 | positive |
| ENSG00000013583 | HEBP1 | 1 | positive |
| ENSG00000015153 | YAF2 | 1 | positive |
| ENSG00000021355 | SERPINB1 | 1 | positive |
| ENSG00000022267 | FHL1 | 1 | positive |
| ENSG00000023191 | RNH1 | 1 | positive |
| ENSG00000023445 | BIRC3 | 1 | positive |
| ENSG00000023697 | DERA | 1 | positive |
| ENSG00000023902 | PLEKHO1 | 1 | positive |
| ENSG00000023909 | GCLM | 1 | positive |
| ENSG00000028277 | POU2F2 | 1 | positive |
| ENSG00000028839 | TBPL1 | 1 | positive |
| ENSG00000029534 | ANK1 | 1 | positive |
| ENSG00000034713 | GABARAPL2 | 1 | positive |
| ENSG00000048740 | CELF2 | 1 | positive |
| ENSG00000049249 | TNFRSF9 | 1 | positive |
| ENSG00000049449 | RCN1 | 1 | positive |
| ENSG00000050393 | MCUR1 | 1 | positive |
| ENSG00000050730 | TNIP3 | 1 | positive |
| ENSG00000051523 | CYBA | 1 | positive |
| ENSG00000054356 | PTPRN | 1 | positive |
| ENSG00000054793 | ATP9A | 1 | positive |
| ENSG00000056558 | TRAF1 | 1 | positive |
| ENSG00000057757 | PITHD1 | 1 | positive |
| ENSG00000059377 | TBXAS1 | 1 | positive |
| ENSG00000059728 | MXD1 | 1 | positive |
| ENSG00000059804 | SLC2A3 | 1 | positive |
| ENSG00000060138 | YBX3 | 1 | positive |
| ENSG00000060558 | GNA15 | 1 | positive |
| ENSG00000061918 | GUCY1B1 | 1 | positive |
| ENSG00000062282 | DGAT2 | 1 | positive |
| ENSG00000062582 | MRPS24 | 1 | positive |
| ENSG00000064225 | ST3GAL6 | 1 | positive |
| ENSG00000064652 | SNX24 | 1 | positive |
| ENSG00000064655 | EYA2 | 1 | positive |
| ENSG00000064666 | CNN2 | 1 | positive |
| ENSG00000065534 | MYLK | 1 | positive |
| ENSG00000066294 | CD84 | 1 | positive |
| ENSG00000066336 | SPI1 | 1 | positive |
| ENSG00000066697 | MSANTD3 | 1 | positive |
| ENSG00000067082 | KLF6 | 1 | positive |
| ENSG00000067225 | PKM | 1 | positive |
| ENSG00000068079 | IFI35 | 1 | positive |
| ENSG00000068354 | TBC1D25 | 1 | positive |
| ENSG00000068796 | KIF2A | 1 | positive |
| ENSG00000069399 | BCL3 | 1 | positive |
| ENSG00000070423 | RNF126 | 1 | positive |
| ENSG00000071051 | NCK2 | 1 | positive |
| ENSG00000071127 | WDR1 | 1 | positive |
| ENSG00000072042 | RDH11 | 1 | positive |
| ENSG00000072135 | PTPN18 | 1 | positive |
| ENSG00000073792 | IGF2BP2 | 1 | positive |
| ENSG00000074416 | MGLL | 1 | positive |
| ENSG00000075415 | SLC25A3 | 1 | positive |
| ENSG00000075624 | ACTB | 1 | positive |
| ENSG00000076944 | STXBP2 | 1 | positive |
| ENSG00000077150 | NFKB2 | 1 | positive |
| ENSG00000077463 | SIRT6 | 1 | positive |
| ENSG00000078668 | VDAC3 | 1 | positive |
| ENSG00000078902 | TOLLIP | 1 | positive |
| ENSG00000080298 | RFX3 | 1 | positive |
| ENSG00000081189 | MEF2C | 1 | positive |
| ENSG00000081377 | CDC14B | 1 | positive |
| ENSG00000082074 | FYB1 | 1 | positive |
| ENSG00000083799 | CYLD | 1 | positive |
| ENSG00000084733 | RAB10 | 1 | positive |
| ENSG00000085117 | CD82 | 1 | positive |
| ENSG00000085733 | CTTN | 1 | positive |
| ENSG00000086189 | DIMT1 | 1 | positive |
| ENSG00000086544 | ITPKC | 1 | positive |
| ENSG00000087269 | NOP14 | 1 | positive |
| ENSG00000087495 | PHACTR3 | 1 | positive |
| ENSG00000088053 | GP6 | 1 | positive |
| ENSG00000088726 | TMEM40 | 1 | positive |
| ENSG00000088826 | SMOX | 1 | positive |
| ENSG00000089327 | FXYD5 | 1 | positive |
| ENSG00000089486 | CDIP1 | 1 | positive |
| ENSG00000090104 | RGS1 | 1 | positive |
| ENSG00000090339 | ICAM1 | 1 | positive |
| ENSG00000090382 | LYZ | 1 | positive |
| ENSG00000092010 | PSME1 | 1 | positive |
| ENSG00000092531 | SNAP23 | 1 | positive |
| ENSG00000095303 | PTGS1 | 1 | positive |
| ENSG00000099194 | SCD | 1 | positive |
| ENSG00000099624 | ATP5F1D | 1 | positive |
| ENSG00000099785 | 2-Mar | 1 | positive |
| ENSG00000099814 | CEP170B | 1 | positive |
| ENSG00000099942 | CRKL | 1 | positive |
| ENSG00000100033 | PRODH | 1 | positive |
| ENSG00000100207 | TCF20 | 1 | positive |
| ENSG00000100226 | GTPBP1 | 1 | positive |
| ENSG00000100243 | CYB5R3 | 1 | positive |
| ENSG00000100297 | MCM5 | 1 | positive |
| ENSG00000100345 | MYH9 | 1 | positive |
| ENSG00000100351 | GRAP2 | 1 | positive |
| ENSG00000100387 | RBX1 | 1 | positive |
| ENSG00000100401 | RANGAP1 | 1 | positive |
| ENSG00000100504 | PYGL | 1 | positive |
| ENSG00000100532 | CGRRF1 | 1 | positive |
| ENSG00000100600 | LGMN | 1 | positive |
| ENSG00000100614 | PPM1A | 1 | positive |
| ENSG00000100902 | PSMA6 | 1 | positive |
| ENSG00000100911 | PSME2 | 1 | positive |
| ENSG00000100985 | MMP9 | 1 | positive |
| ENSG00000100994 | PYGB | 1 | positive |
| ENSG00000101082 | SLA2 | 1 | positive |
| ENSG00000101160 | CTSZ | 1 | positive |
| ENSG00000101162 | TUBB1 | 1 | positive |
| ENSG00000101236 | RNF24 | 1 | positive |
| ENSG00000101335 | MYL9 | 1 | positive |
| ENSG00000101441 | CST4 | 1 | positive |
| ENSG00000101745 | ANKRD12 | 1 | positive |
| ENSG00000101856 | PGRMC1 | 1 | positive |
| ENSG00000102145 | GATA1 | 1 | positive |
| ENSG00000102178 | UBL4A | 1 | positive |
| ENSG00000102245 | CD40LG | 1 | positive |
| ENSG00000102265 | TIMP1 | 1 | positive |
| ENSG00000102393 | GLA | 1 | positive |
| ENSG00000102468 | HTR2A | 1 | positive |
| ENSG00000102554 | KLF5 | 1 | positive |
| ENSG00000102572 | STK24 | 1 | positive |
| ENSG00000102575 | ACP5 | 1 | positive |
| ENSG00000102760 | RGCC | 1 | positive |
| ENSG00000102804 | TSC22D1 | 1 | positive |
| ENSG00000102879 | CORO1A | 1 | positive |
| ENSG00000102901 | CENPT | 1 | positive |
| ENSG00000103184 | SEC14L5 | 1 | positive |
| ENSG00000103512 | NOMO1 | 1 | positive |
| ENSG00000103740 | ACSBG1 | 1 | positive |
| ENSG00000103769 | RAB11A | 1 | positive |
| ENSG00000104267 | CA2 | 1 | positive |
| ENSG00000104312 | RIPK2 | 1 | positive |
| ENSG00000104432 | IL7 | 1 | positive |
| ENSG00000104763 | ASAH1 | 1 | positive |
| ENSG00000104856 | RELB | 1 | positive |
| ENSG00000104903 | LYL1 | 1 | positive |
| ENSG00000104904 | OAZ1 | 1 | positive |
| ENSG00000104951 | IL4I1 | 1 | positive |
| ENSG00000104998 | IL27RA | 1 | positive |
| ENSG00000105193 | RPS16 | 1 | positive |
| ENSG00000105202 | FBL | 1 | positive |
| ENSG00000105246 | EBI3 | 1 | positive |
| ENSG00000105325 | FZR1 | 1 | positive |
| ENSG00000105327 | BBC3 | 1 | positive |
| ENSG00000105329 | TGFB1 | 1 | positive |
| ENSG00000105339 | DENND3 | 1 | positive |
| ENSG00000105388 | CEACAM5 | 1 | positive |
| ENSG00000105438 | KDELR1 | 1 | positive |
| ENSG00000105483 | CARD8 | 1 | positive |
| ENSG00000105499 | PLA2G4C | 1 | positive |
| ENSG00000105698 | USF2 | 1 | positive |
| ENSG00000105701 | FKBP8 | 1 | positive |
| ENSG00000105711 | SCN1B | 1 | positive |
| ENSG00000105810 | CDK6 | 1 | positive |
| ENSG00000105825 | TFPI2 | 1 | positive |
| ENSG00000105835 | NAMPT | 1 | positive |
| ENSG00000105887 | MTPN | 1 | positive |
| ENSG00000105894 | PTN | 1 | positive |
| ENSG00000105974 | CAV1 | 1 | positive |
| ENSG00000106546 | AHR | 1 | positive |
| ENSG00000106733 | NMRK1 | 1 | positive |
| ENSG00000106868 | SUSD1 | 1 | positive |
| ENSG00000107738 | VSIR | 1 | positive |
| ENSG00000107863 | ARHGAP21 | 1 | positive |
| ENSG00000108039 | XPNPEP1 | 1 | positive |
| ENSG00000108100 | CCNY | 1 | positive |
| ENSG00000108179 | PPIF | 1 | positive |
| ENSG00000108518 | PFN1 | 1 | positive |
| ENSG00000108551 | RASD1 | 1 | positive |
| ENSG00000108576 | SLC6A4 | 1 | positive |
| ENSG00000108821 | COL1A1 | 1 | positive |
| ENSG00000108960 | MMD | 1 | positive |
| ENSG00000109066 | TMEM104 | 1 | positive |
| ENSG00000109220 | CHIC2 | 1 | positive |
| ENSG00000109272 | PF4V1 | 1 | positive |
| ENSG00000109320 | NFKB1 | 1 | positive |
| ENSG00000109321 | AREG | 1 | positive |
| ENSG00000109610 | SOD3 | 1 | positive |
| ENSG00000110013 | SIAE | 1 | positive |
| ENSG00000110057 | UNC93B1 | 1 | positive |
| ENSG00000110218 | PANX1 | 1 | positive |
| ENSG00000110436 | SLC1A2 | 1 | positive |
| ENSG00000110446 | SLC15A3 | 1 | positive |
| ENSG00000110536 | PTPMT1 | 1 | positive |
| ENSG00000110700 | RPS13 | 1 | positive |
| ENSG00000110719 | TCIRG1 | 1 | positive |
| ENSG00000110799 | VWF | 1 | positive |
| ENSG00000110880 | CORO1C | 1 | positive |
| ENSG00000110934 | BIN2 | 1 | positive |
| ENSG00000110955 | ATP5F1B | 1 | positive |
| ENSG00000111252 | SH2B3 | 1 | positive |
| ENSG00000111331 | OAS3 | 1 | positive |
| ENSG00000111348 | ARHGDIB | 1 | positive |
| ENSG00000111424 | VDR | 1 | positive |
| ENSG00000111640 | GAPDH | 1 | positive |
| ENSG00000111644 | ACRBP | 1 | positive |
| ENSG00000111678 | C12orf57 | 1 | positive |
| ENSG00000111817 | DSE | 1 | positive |
| ENSG00000111912 | NCOA7 | 1 | positive |
| ENSG00000111913 | RIPOR2 | 1 | positive |
| ENSG00000112149 | CD83 | 1 | positive |
| ENSG00000112245 | PTP4A1 | 1 | positive |
| ENSG00000112531 | QKI | 1 | positive |
| ENSG00000112576 | CCND3 | 1 | positive |
| ENSG00000112699 | GMDS | 1 | positive |
| ENSG00000113070 | HBEGF | 1 | positive |
| ENSG00000113140 | SPARC | 1 | positive |
| ENSG00000113645 | WWC1 | 1 | positive |
| ENSG00000113851 | CRBN | 1 | positive |
| ENSG00000113924 | HGD | 1 | positive |
| ENSG00000114850 | SSR3 | 1 | positive |
| ENSG00000114978 | MOB1A | 1 | positive |
| ENSG00000115112 | TFCP2L1 | 1 | positive |
| ENSG00000115415 | STAT1 | 1 | positive |
| ENSG00000115474 | KCNJ13 | 1 | positive |
| ENSG00000115758 | ODC1 | 1 | positive |
| ENSG00000115935 | WIPF1 | 1 | positive |
| ENSG00000115956 | PLEK | 1 | positive |
| ENSG00000116473 | RAP1A | 1 | positive |
| ENSG00000116717 | GADD45A | 1 | positive |
| ENSG00000116815 | CD58 | 1 | positive |
| ENSG00000117155 | SSX2IP | 1 | positive |
| ENSG00000117228 | GBP1 | 1 | positive |
| ENSG00000117525 | F3 | 1 | positive |
| ENSG00000117592 | PRDX6 | 1 | positive |
| ENSG00000118407 | FILIP1 | 1 | positive |
| ENSG00000118503 | TNFAIP3 | 1 | positive |
| ENSG00000118515 | SGK1 | 1 | positive |
| ENSG00000118785 | SPP1 | 1 | positive |
| ENSG00000118855 | MFSD1 | 1 | positive |
| ENSG00000118985 | ELL2 | 1 | positive |
| ENSG00000119138 | KLF9 | 1 | positive |
| ENSG00000119242 | CCDC92 | 1 | positive |
| ENSG00000119632 | IFI27L2 | 1 | positive |
| ENSG00000119684 | MLH3 | 1 | positive |
| ENSG00000119714 | GPR68 | 1 | positive |
| ENSG00000119862 | LGALSL | 1 | positive |
| ENSG00000119917 | IFIT3 | 1 | positive |
| ENSG00000120279 | MYCT1 | 1 | positive |
| ENSG00000120306 | CYSTM1 | 1 | positive |
| ENSG00000120438 | TCP1 | 1 | positive |
| ENSG00000120594 | PLXDC2 | 1 | positive |
| ENSG00000120738 | EGR1 | 1 | positive |
| ENSG00000120885 | CLU | 1 | positive |
| ENSG00000120889 | TNFRSF10B | 1 | positive |
| ENSG00000121766 | ZCCHC17 | 1 | positive |
| ENSG00000122643 | NT5C3A | 1 | positive |
| ENSG00000122861 | PLAU | 1 | positive |
| ENSG00000122862 | SRGN | 1 | positive |
| ENSG00000123091 | RNF11 | 1 | positive |
| ENSG00000123095 | BHLHE41 | 1 | positive |
| ENSG00000123405 | NFE2 | 1 | positive |
| ENSG00000123505 | AMD1 | 1 | positive |
| ENSG00000123610 | TNFAIP6 | 1 | positive |
| ENSG00000123689 | G0S2 | 1 | positive |
| ENSG00000123739 | PLA2G12A | 1 | positive |
| ENSG00000124145 | SDC4 | 1 | positive |
| ENSG00000124491 | F13A1 | 1 | positive |
| ENSG00000124688 | MAD2L1BP | 1 | positive |
| ENSG00000124795 | DEK | 1 | positive |
| ENSG00000124813 | RUNX2 | 1 | positive |
| ENSG00000124882 | EREG | 1 | positive |
| ENSG00000125037 | EMC3 | 1 | positive |
| ENSG00000125148 | MT2A | 1 | positive |
| ENSG00000125266 | EFNB2 | 1 | positive |
| ENSG00000125354 | 6-Sep | 1 | positive |
| ENSG00000125457 | MIF4GD | 1 | positive |
| ENSG00000125657 | TNFSF9 | 1 | positive |
| ENSG00000125744 | RTN2 | 1 | positive |
| ENSG00000125753 | VASP | 1 | positive |
| ENSG00000125826 | RBCK1 | 1 | positive |
| ENSG00000125844 | RRBP1 | 1 | positive |
| ENSG00000125898 | FAM110A | 1 | positive |
| ENSG00000125952 | MAX | 1 | positive |
| ENSG00000125998 | FAM83C | 1 | positive |
| ENSG00000126709 | IFI6 | 1 | positive |
| ENSG00000126749 | EMG1 | 1 | positive |
| ENSG00000127533 | F2RL3 | 1 | positive |
| ENSG00000127666 | TICAM1 | 1 | positive |
| ENSG00000127824 | TUBA4A | 1 | positive |
| ENSG00000127920 | GNG11 | 1 | positive |
| ENSG00000127947 | PTPN12 | 1 | positive |
| ENSG00000128228 | SDF2L1 | 1 | positive |
| ENSG00000128245 | YWHAH | 1 | positive |
| ENSG00000128266 | GNAZ | 1 | positive |
| ENSG00000128272 | ATF4 | 1 | positive |
| ENSG00000128294 | TPST2 | 1 | positive |
| ENSG00000128309 | MPST | 1 | positive |
| ENSG00000128311 | TST | 1 | positive |
| ENSG00000128340 | RAC2 | 1 | positive |
| ENSG00000128342 | LIF | 1 | positive |
| ENSG00000128739 | SNRPN | 1 | positive |
| ENSG00000129226 | CD68 | 1 | positive |
| ENSG00000129355 | CDKN2D | 1 | positive |
| ENSG00000129667 | RHBDF2 | 1 | positive |
| ENSG00000130255 | RPL36 | 1 | positive |
| ENSG00000130429 | ARPC1B | 1 | positive |
| ENSG00000130830 | MPP1 | 1 | positive |
| ENSG00000130958 | SLC35D2 | 1 | positive |
| ENSG00000131323 | TRAF3 | 1 | positive |
| ENSG00000131459 | GFPT2 | 1 | positive |
| ENSG00000131669 | NINJ1 | 1 | positive |
| ENSG00000131725 | WDR44 | 1 | positive |
| ENSG00000132383 | RPA1 | 1 | positive |
| ENSG00000132471 | WBP2 | 1 | positive |
| ENSG00000132530 | XAF1 | 1 | positive |
| ENSG00000132970 | WASF3 | 1 | positive |
| ENSG00000133069 | TMCC2 | 1 | positive |
| ENSG00000133317 | LGALS12 | 1 | positive |
| ENSG00000134030 | CTIF | 1 | positive |
| ENSG00000134070 | IRAK2 | 1 | positive |
| ENSG00000134317 | GRHL1 | 1 | positive |
| ENSG00000134318 | ROCK2 | 1 | positive |
| ENSG00000134339 | SAA2 | 1 | positive |
| ENSG00000134352 | IL6ST | 1 | positive |
| ENSG00000134802 | SLC43A3 | 1 | positive |
| ENSG00000134824 | FADS2 | 1 | positive |
| ENSG00000135070 | ISCA1 | 1 | positive |
| ENSG00000135185 | TMEM243 | 1 | positive |
| ENSG00000135218 | CD36 | 1 | positive |
| ENSG00000135334 | AKIRIN2 | 1 | positive |
| ENSG00000135373 | EHF | 1 | positive |
| ENSG00000135549 | PKIB | 1 | positive |
| ENSG00000135604 | STX11 | 1 | positive |
| ENSG00000135678 | CPM | 1 | positive |
| ENSG00000135905 | DOCK10 | 1 | positive |
| ENSG00000135926 | TMBIM1 | 1 | positive |
| ENSG00000136048 | DRAM1 | 1 | positive |
| ENSG00000136052 | SLC41A2 | 1 | positive |
| ENSG00000136153 | LMO7 | 1 | positive |
| ENSG00000136156 | ITM2B | 1 | positive |
| ENSG00000136231 | IGF2BP3 | 1 | positive |
| ENSG00000136238 | RAC1 | 1 | positive |
| ENSG00000136279 | DBNL | 1 | positive |
| ENSG00000136404 | TM6SF1 | 1 | positive |
| ENSG00000136490 | LIMD2 | 1 | positive |
| ENSG00000136754 | ABI1 | 1 | positive |
| ENSG00000136826 | KLF4 | 1 | positive |
| ENSG00000136929 | HEMGN | 1 | positive |
| ENSG00000137198 | GMPR | 1 | positive |
| ENSG00000137947 | GTF2B | 1 | positive |
| ENSG00000137965 | IFI44 | 1 | positive |
| ENSG00000138135 | CH25H | 1 | positive |
| ENSG00000138166 | DUSP5 | 1 | positive |
| ENSG00000138378 | STAT4 | 1 | positive |
| ENSG00000138449 | SLC40A1 | 1 | positive |
| ENSG00000138670 | RASGEF1B | 1 | positive |
| ENSG00000138722 | MMRN1 | 1 | positive |
| ENSG00000139117 | CPNE8 | 1 | positive |
| ENSG00000139318 | DUSP6 | 1 | positive |
| ENSG00000139725 | RHOF | 1 | positive |
| ENSG00000140299 | BNIP2 | 1 | positive |
| ENSG00000140379 | BCL2A1 | 1 | positive |
| ENSG00000140416 | TPM1 | 1 | positive |
| ENSG00000140479 | PCSK6 | 1 | positive |
| ENSG00000140548 | ZNF710 | 1 | positive |
| ENSG00000140682 | TGFB1I1 | 1 | positive |
| ENSG00000140853 | NLRC5 | 1 | positive |
| ENSG00000141873 | SLC39A3 | 1 | positive |
| ENSG00000142046 | TMEM91 | 1 | positive |
| ENSG00000142089 | IFITM3 | 1 | positive |
| ENSG00000142227 | EMP3 | 1 | positive |
| ENSG00000142634 | EFHD2 | 1 | positive |
| ENSG00000142961 | MOB3C | 1 | positive |
| ENSG00000143324 | XPR1 | 1 | positive |
| ENSG00000143353 | LYPLAL1 | 1 | positive |
| ENSG00000143363 | PRUNE1 | 1 | positive |
| ENSG00000143384 | MCL1 | 1 | positive |
| ENSG00000143409 | MINDY1 | 1 | positive |
| ENSG00000143622 | RIT1 | 1 | positive |
| ENSG00000143858 | SYT2 | 1 | positive |
| ENSG00000144677 | CTDSPL | 1 | positive |
| ENSG00000144746 | ARL6IP5 | 1 | positive |
| ENSG00000144893 | MED12L | 1 | positive |
| ENSG00000145331 | TRMT10A | 1 | positive |
| ENSG00000145335 | SNCA | 1 | positive |
| ENSG00000145592 | RPL37 | 1 | positive |
| ENSG00000145685 | LHFPL2 | 1 | positive |
| ENSG00000145741 | BTF3 | 1 | positive |
| ENSG00000145779 | TNFAIP8 | 1 | positive |
| ENSG00000145901 | TNIP1 | 1 | positive |
| ENSG00000146232 | NFKBIE | 1 | positive |
| ENSG00000146278 | PNRC1 | 1 | positive |
| ENSG00000146834 | MEPCE | 1 | positive |
| ENSG00000146859 | TMEM140 | 1 | positive |
| ENSG00000147394 | ZNF185 | 1 | positive |
| ENSG00000147526 | TACC1 | 1 | positive |
| ENSG00000147650 | LRP12 | 1 | positive |
| ENSG00000147883 | CDKN2B | 1 | positive |
| ENSG00000148154 | UGCG | 1 | positive |
| ENSG00000148175 | STOM | 1 | positive |
| ENSG00000148346 | LCN2 | 1 | positive |
| ENSG00000148484 | RSU1 | 1 | positive |
| ENSG00000148677 | ANKRD1 | 1 | positive |
| ENSG00000148834 | GSTO1 | 1 | positive |
| ENSG00000148908 | RGS10 | 1 | positive |
| ENSG00000149177 | PTPRJ | 1 | positive |
| ENSG00000149218 | ENDOD1 | 1 | positive |
| ENSG00000149564 | ESAM | 1 | positive |
| ENSG00000149781 | FERMT3 | 1 | positive |
| ENSG00000149798 | CDC42EP2 | 1 | positive |
| ENSG00000149806 | FAU | 1 | positive |
| ENSG00000149948 | HMGA2 | 1 | positive |
| ENSG00000150637 | CD226 | 1 | positive |
| ENSG00000150681 | RGS18 | 1 | positive |
| ENSG00000150867 | PIP4K2A | 1 | positive |
| ENSG00000150991 | UBC | 1 | positive |
| ENSG00000151136 | BTBD11 | 1 | positive |
| ENSG00000151702 | FLI1 | 1 | positive |
| ENSG00000151748 | SAV1 | 1 | positive |
| ENSG00000152484 | USP12 | 1 | positive |
| ENSG00000152601 | MBNL1 | 1 | positive |
| ENSG00000152684 | PELO | 1 | positive |
| ENSG00000152952 | PLOD2 | 1 | positive |
| ENSG00000153046 | CDYL | 1 | positive |
| ENSG00000153815 | CMIP | 1 | positive |
| ENSG00000154127 | UBASH3B | 1 | positive |
| ENSG00000154146 | NRGN | 1 | positive |
| ENSG00000154305 | MIA3 | 1 | positive |
| ENSG00000154451 | GBP5 | 1 | positive |
| ENSG00000154639 | CXADR | 1 | positive |
| ENSG00000155324 | GRAMD2B | 1 | positive |
| ENSG00000155657 | TTN | 1 | positive |
| ENSG00000156265 | MAP3K7CL | 1 | positive |
| ENSG00000156273 | BACH1 | 1 | positive |
| ENSG00000156482 | RPL30 | 1 | positive |
| ENSG00000156587 | UBE2L6 | 1 | positive |
| ENSG00000156642 | NPTN | 1 | positive |
| ENSG00000156738 | MS4A1 | 1 | positive |
| ENSG00000157601 | MX1 | 1 | positive |
| ENSG00000157873 | TNFRSF14 | 1 | positive |
| ENSG00000157978 | LDLRAP1 | 1 | positive |
| ENSG00000158062 | UBXN11 | 1 | positive |
| ENSG00000158457 | TSPAN33 | 1 | positive |
| ENSG00000158552 | ZFAND2B | 1 | positive |
| ENSG00000158710 | TAGLN2 | 1 | positive |
| ENSG00000158856 | DMTN | 1 | positive |
| ENSG00000158869 | FCER1G | 1 | positive |
| ENSG00000159128 | IFNGR2 | 1 | positive |
| ENSG00000159202 | UBE2Z | 1 | positive |
| ENSG00000159210 | SNF8 | 1 | positive |
| ENSG00000159231 | CBR3 | 1 | positive |
| ENSG00000159339 | PADI4 | 1 | positive |
| ENSG00000160013 | PTGIR | 1 | positive |
| ENSG00000160014 | CALM3 | 1 | positive |
| ENSG00000160050 | CCDC28B | 1 | positive |
| ENSG00000160145 | KALRN | 1 | positive |
| ENSG00000160223 | ICOSLG | 1 | positive |
| ENSG00000160593 | JAML | 1 | positive |
| ENSG00000160932 | LY6E | 1 | positive |
| ENSG00000161791 | FMNL3 | 1 | positive |
| ENSG00000161911 | TREML1 | 1 | positive |
| ENSG00000162366 | PDZK1IP1 | 1 | positive |
| ENSG00000162367 | TAL1 | 1 | positive |
| ENSG00000162368 | CMPK1 | 1 | positive |
| ENSG00000162511 | LAPTM5 | 1 | positive |
| ENSG00000162614 | NEXN | 1 | positive |
| ENSG00000162704 | ARPC5 | 1 | positive |
| ENSG00000162722 | TRIM58 | 1 | positive |
| ENSG00000162889 | MAPKAPK2 | 1 | positive |
| ENSG00000163017 | ACTG2 | 1 | positive |
| ENSG00000163082 | SGPP2 | 1 | positive |
| ENSG00000163110 | PDLIM5 | 1 | positive |
| ENSG00000163171 | CDC42EP3 | 1 | positive |
| ENSG00000163346 | PBXIP1 | 1 | positive |
| ENSG00000163347 | CLDN1 | 1 | positive |
| ENSG00000163435 | ELF3 | 1 | positive |
| ENSG00000163545 | NUAK2 | 1 | positive |
| ENSG00000163660 | CCNL1 | 1 | positive |
| ENSG00000163661 | PTX3 | 1 | positive |
| ENSG00000163734 | CXCL3 | 1 | positive |
| ENSG00000163735 | CXCL5 | 1 | positive |
| ENSG00000163736 | PPBP | 1 | positive |
| ENSG00000163737 | PF4 | 1 | positive |
| ENSG00000163743 | RCHY1 | 1 | positive |
| ENSG00000163812 | ZDHHC3 | 1 | positive |
| ENSG00000163950 | SLBP | 1 | positive |
| ENSG00000163993 | S100P | 1 | positive |
| ENSG00000164181 | ELOVL7 | 1 | positive |
| ENSG00000164236 | ANKRD33B | 1 | positive |
| ENSG00000164400 | CSF2 | 1 | positive |
| ENSG00000164434 | FABP7 | 1 | positive |
| ENSG00000165309 | ARMC3 | 1 | positive |
| ENSG00000165475 | CRYL1 | 1 | positive |
| ENSG00000165496 | RPL10L | 1 | positive |
| ENSG00000165516 | KLHDC2 | 1 | positive |
| ENSG00000165609 | NUDT5 | 1 | positive |
| ENSG00000165682 | CLEC1B | 1 | positive |
| ENSG00000165702 | GFI1B | 1 | positive |
| ENSG00000165801 | ARHGEF40 | 1 | positive |
| ENSG00000166091 | CMTM5 | 1 | positive |
| ENSG00000166148 | AVPR1A | 1 | positive |
| ENSG00000166333 | ILK | 1 | positive |
| ENSG00000166337 | TAF10 | 1 | positive |
| ENSG00000166441 | RPL27A | 1 | positive |
| ENSG00000166501 | PRKCB | 1 | positive |
| ENSG00000166508 | MCM7 | 1 | positive |
| ENSG00000166741 | NNMT | 1 | positive |
| ENSG00000166920 | C15orf48 | 1 | positive |
| ENSG00000166949 | SMAD3 | 1 | positive |
| ENSG00000166963 | MAP1A | 1 | positive |
| ENSG00000166974 | MAPRE2 | 1 | positive |
| ENSG00000167034 | NKX3-1 | 1 | positive |
| ENSG00000167100 | SAMD14 | 1 | positive |
| ENSG00000167460 | TPM4 | 1 | positive |
| ENSG00000167461 | RAB8A | 1 | positive |
| ENSG00000167553 | TUBA1C | 1 | positive |
| ENSG00000167657 | DAPK3 | 1 | positive |
| ENSG00000167779 | IGFBP6 | 1 | positive |
| ENSG00000167978 | SRRM2 | 1 | positive |
| ENSG00000167996 | FTH1 | 1 | positive |
| ENSG00000168067 | MAP4K2 | 1 | positive |
| ENSG00000168461 | RAB31 | 1 | positive |
| ENSG00000168497 | CAVIN2 | 1 | positive |
| ENSG00000168685 | IL7R | 1 | positive |
| ENSG00000168734 | PKIG | 1 | positive |
| ENSG00000168802 | CHTF8 | 1 | positive |
| ENSG00000168884 | TNIP2 | 1 | positive |
| ENSG00000169026 | SLC49A3 | 1 | positive |
| ENSG00000169067 | ACTBL2 | 1 | positive |
| ENSG00000169193 | CCDC126 | 1 | positive |
| ENSG00000169242 | EFNA1 | 1 | positive |
| ENSG00000169245 | CXCL10 | 1 | positive |
| ENSG00000169313 | P2RY12 | 1 | positive |
| ENSG00000169490 | TM2D2 | 1 | positive |
| ENSG00000169554 | ZEB2 | 1 | positive |
| ENSG00000169704 | GP9 | 1 | positive |
| ENSG00000169715 | MT1E | 1 | positive |
| ENSG00000169756 | LIMS1 | 1 | positive |
| ENSG00000169860 | P2RY1 | 1 | positive |
| ENSG00000169891 | REPS2 | 1 | positive |
| ENSG00000169908 | TM4SF1 | 1 | positive |
| ENSG00000170035 | UBE2E3 | 1 | positive |
| ENSG00000170271 | FAXDC2 | 1 | positive |
| ENSG00000170485 | NPAS2 | 1 | positive |
| ENSG00000170498 | KISS1 | 1 | positive |
| ENSG00000171206 | TRIM8 | 1 | positive |
| ENSG00000171223 | JUNB | 1 | positive |
| ENSG00000171385 | KCND3 | 1 | positive |
| ENSG00000171492 | LRRC8D | 1 | positive |
| ENSG00000171552 | BCL2L1 | 1 | positive |
| ENSG00000171611 | PTCRA | 1 | positive |
| ENSG00000171766 | GATM | 1 | positive |
| ENSG00000171773 | NXNL1 | 1 | positive |
| ENSG00000172216 | CEBPB | 1 | positive |
| ENSG00000172349 | IL16 | 1 | positive |
| ENSG00000172432 | GTPBP2 | 1 | positive |
| ENSG00000172572 | PDE3A | 1 | positive |
| ENSG00000172590 | MRPL52 | 1 | positive |
| ENSG00000172794 | RAB37 | 1 | positive |
| ENSG00000172893 | DHCR7 | 1 | positive |
| ENSG00000172927 | MYEOV | 1 | positive |
| ENSG00000173193 | PARP14 | 1 | positive |
| ENSG00000173212 | MAB21L3 | 1 | positive |
| ENSG00000173218 | VANGL1 | 1 | positive |
| ENSG00000173432 | SAA1 | 1 | positive |
| ENSG00000173465 | SSSCA1 | 1 | positive |
| ENSG00000173540 | GMPPB | 1 | positive |
| ENSG00000173626 | TRAPPC3L | 1 | positive |
| ENSG00000173918 | C1QTNF1 | 1 | positive |
| ENSG00000174099 | MSRB3 | 1 | positive |
| ENSG00000174175 | SELP | 1 | positive |
| ENSG00000174574 | AKIRIN1 | 1 | positive |
| ENSG00000175390 | EIF3F | 1 | positive |
| ENSG00000175416 | CLTB | 1 | positive |
| ENSG00000175538 | KCNE3 | 1 | positive |
| ENSG00000175550 | DRAP1 | 1 | positive |
| ENSG00000175592 | FOSL1 | 1 | positive |
| ENSG00000175806 | MSRA | 1 | positive |
| ENSG00000175854 | SWI5 | 1 | positive |
| ENSG00000176597 | B3GNT5 | 1 | positive |
| ENSG00000176783 | RUFY1 | 1 | positive |
| ENSG00000177105 | RHOG | 1 | positive |
| ENSG00000177119 | ANO6 | 1 | positive |
| ENSG00000177156 | TALDO1 | 1 | positive |
| ENSG00000177324 | BEND2 | 1 | positive |
| ENSG00000177465 | ACOT4 | 1 | positive |
| ENSG00000177542 | SLC25A22 | 1 | positive |
| ENSG00000177666 | PNPLA2 | 1 | positive |
| ENSG00000177700 | POLR2L | 1 | positive |
| ENSG00000177989 | ODF3B | 1 | positive |
| ENSG00000178033 | CALHM5 | 1 | positive |
| ENSG00000178449 | COX14 | 1 | positive |
| ENSG00000178719 | GRINA | 1 | positive |
| ENSG00000178772 | CPN2 | 1 | positive |
| ENSG00000179010 | MRFAP1 | 1 | positive |
| ENSG00000179046 | TRIML2 | 1 | positive |
| ENSG00000179057 | IGSF22 | 1 | positive |
| ENSG00000179091 | CYC1 | 1 | positive |
| ENSG00000179388 | EGR3 | 1 | positive |
| ENSG00000179826 | MRGPRX3 | 1 | positive |
| ENSG00000180190 | TDRP | 1 | positive |
| ENSG00000180353 | HCLS1 | 1 | positive |
| ENSG00000180354 | MTURN | 1 | positive |
| ENSG00000180573 | HIST1H2AC | 1 | positive |
| ENSG00000180596 | HIST1H2BC | 1 | positive |
| ENSG00000180628 | PCGF5 | 1 | positive |
| ENSG00000180694 | TMEM64 | 1 | positive |
| ENSG00000180879 | SSR4 | 1 | positive |
| ENSG00000180914 | OXTR | 1 | positive |
| ENSG00000181029 | TRAPPC5 | 1 | positive |
| ENSG00000181104 | F2R | 1 | positive |
| ENSG00000181218 | HIST3H2A | 1 | positive |
| ENSG00000181634 | TNFSF15 | 1 | positive |
| ENSG00000181751 | C5orf30 | 1 | positive |
| ENSG00000181788 | SIAH2 | 1 | positive |
| ENSG00000181830 | SLC35C1 | 1 | positive |
| ENSG00000181924 | COA4 | 1 | positive |
| ENSG00000182208 | MOB2 | 1 | positive |
| ENSG00000182287 | AP1S2 | 1 | positive |
| ENSG00000182326 | C1S | 1 | positive |
| ENSG00000183508 | FAM46C | 1 | positive |
| ENSG00000183621 | ZNF438 | 1 | positive |
| ENSG00000183690 | EFHC2 | 1 | positive |
| ENSG00000183722 | LHFPL6 | 1 | positive |
| ENSG00000183785 | TUBA8 | 1 | positive |
| ENSG00000184007 | PTP4A2 | 1 | positive |
| ENSG00000184113 | CLDN5 | 1 | positive |
| ENSG00000184226 | PCDH9 | 1 | positive |
| ENSG00000184371 | CSF1 | 1 | positive |
| ENSG00000184489 | PTP4A3 | 1 | positive |
| ENSG00000184500 | PROS1 | 1 | positive |
| ENSG00000184602 | SNN | 1 | positive |
| ENSG00000184678 | HIST2H2BE | 1 | positive |
| ENSG00000184967 | NOC4L | 1 | positive |
| ENSG00000184990 | SIVA1 | 1 | positive |
| ENSG00000185033 | SEMA4B | 1 | positive |
| ENSG00000185043 | CIB1 | 1 | positive |
| ENSG00000185164 | NOMO2 | 1 | positive |
| ENSG00000185201 | IFITM2 | 1 | positive |
| ENSG00000185215 | TNFAIP2 | 1 | positive |
| ENSG00000185245 | GP1BA | 1 | positive |
| ENSG00000185340 | GAS2L1 | 1 | positive |
| ENSG00000185475 | TMEM179B | 1 | positive |
| ENSG00000185507 | IRF7 | 1 | positive |
| ENSG00000185614 | FAM212A | 1 | positive |
| ENSG00000185947 | ZNF267 | 1 | positive |
| ENSG00000185963 | BICD2 | 1 | positive |
| ENSG00000186468 | RPS23 | 1 | positive |
| ENSG00000186480 | INSIG1 | 1 | positive |
| ENSG00000186787 | SPIN2B | 1 | positive |
| ENSG00000187109 | NAP1L1 | 1 | positive |
| ENSG00000187266 | EPOR | 1 | positive |
| ENSG00000187699 | C2orf88 | 1 | positive |
| ENSG00000187764 | SEMA4D | 1 | positive |
| ENSG00000188060 | RAB42 | 1 | positive |
| ENSG00000188191 | PRKAR1B | 1 | positive |
| ENSG00000188313 | PLSCR1 | 1 | positive |
| ENSG00000188375 | H3F3C | 1 | positive |
| ENSG00000188641 | DPYD | 1 | positive |
| ENSG00000188677 | PARVB | 1 | positive |
| ENSG00000188921 | HACD4 | 1 | positive |
| ENSG00000196747 | HIST1H2AI | 1 | positive |
| ENSG00000196878 | LAMB3 | 1 | positive |
| ENSG00000196924 | FLNA | 1 | positive |
| ENSG00000196954 | CASP4 | 1 | positive |
| ENSG00000197046 | SIGLEC15 | 1 | positive |
| ENSG00000197122 | SRC | 1 | positive |
| ENSG00000197249 | SERPINA1 | 1 | positive |
| ENSG00000197343 | ZNF655 | 1 | positive |
| ENSG00000197446 | CYP2F1 | 1 | positive |
| ENSG00000197653 | DNAH10 | 1 | positive |
| ENSG00000197822 | OCLN | 1 | positive |
| ENSG00000197959 | DNM3 | 1 | positive |
| ENSG00000197965 | MPZL1 | 1 | positive |
| ENSG00000198242 | RPL23A | 1 | positive |
| ENSG00000198478 | SH3BGRL2 | 1 | positive |
| ENSG00000198513 | ATL1 | 1 | positive |
| ENSG00000198573 | SPANXC | 1 | positive |
| ENSG00000198586 | TLK1 | 1 | positive |
| ENSG00000198805 | PNP | 1 | positive |
| ENSG00000198832 | SELENOM | 1 | positive |
| ENSG00000198858 | R3HDM4 | 1 | positive |
| ENSG00000198873 | GRK5 | 1 | positive |
| ENSG00000198898 | CAPZA2 | 1 | positive |
| ENSG00000198911 | SREBF2 | 1 | positive |
| ENSG00000198924 | DCLRE1A | 1 | positive |
| ENSG00000198948 | MFAP3L | 1 | positive |
| ENSG00000198959 | TGM2 | 1 | positive |
| ENSG00000203485 | INF2 | 1 | positive |
| ENSG00000203618 | GP1BB | 1 | positive |
| ENSG00000204264 | PSMB8 | 1 | positive |
| ENSG00000204287 | HLA-DRA | 1 | positive |
| ENSG00000204310 | AGPAT1 | 1 | positive |
| ENSG00000204323 | SMIM5 | 1 | positive |
| ENSG00000204381 | LAYN | 1 | positive |
| ENSG00000204388 | HSPA1B | 1 | positive |
| ENSG00000204389 | HSPA1A | 1 | positive |
| ENSG00000204420 | MPIG6B | 1 | positive |
| ENSG00000204427 | ABHD16A | 1 | positive |
| ENSG00000204428 | LY6G5C | 1 | positive |
| ENSG00000204472 | AIF1 | 1 | positive |
| ENSG00000204574 | ABCF1 | 1 | positive |
| ENSG00000204632 | HLA-G | 1 | positive |
| ENSG00000204843 | DCTN1 | 1 | positive |
| ENSG00000205220 | PSMB10 | 1 | positive |
| ENSG00000205352 | PRR13 | 1 | positive |
| ENSG00000205426 | KRT81 | 1 | positive |
| ENSG00000205639 | MFSD2B | 1 | positive |
| ENSG00000211455 | STK38L | 1 | positive |
| ENSG00000212864 | RNF208 | 1 | positive |
| ENSG00000213741 | RPS29 | 1 | positive |
| ENSG00000214530 | STARD10 | 1 | positive |
| ENSG00000216490 | IFI30 | 1 | positive |
| ENSG00000219200 | RNASEK | 1 | positive |
| ENSG00000220201 | ZGLP1 | 1 | positive |
| ENSG00000221823 | PPP3R1 | 1 | positive |
| ENSG00000221963 | APOL6 | 1 | positive |
| ENSG00000222038 | POTEJ | 1 | positive |
| ENSG00000227234 | SPANXB1 | 1 | positive |
| ENSG00000227507 | LTB | 1 | positive |
| ENSG00000232629 | HLA-DQB2 | 1 | positive |
| ENSG00000241360 | PDXP | 1 | positive |
| ENSG00000243244 | STON1 | 1 | positive |
| ENSG00000243449 | C4orf48 | 1 | positive |
| ENSG00000243678 | NME2 | 1 | positive |
| ENSG00000244122 | UGT1A7 | 1 | positive |
| ENSG00000244274 | DBNDD2 | 1 | positive |
| ENSG00000246705 | H2AFJ | 1 | positive |
| ENSG00000247596 | TWF2 | 1 | positive |
| ENSG00000249992 | TMEM158 | 1 | positive |
| ENSG00000254087 | LYN | 1 | positive |
| ENSG00000254726 | MEX3A | 1 | positive |
| ENSG00000256045 | MTRNR2L10 | 1 | positive |
| ENSG00000256235 | SMIM3 | 1 | positive |
| ENSG00000256618 | MTRNR2L1 | 1 | positive |
| ENSG00000258315 | C17orf49 | 1 | positive |
| ENSG00000258366 | RTEL1 | 1 | positive |
| ENSG00000259207 | ITGB3 | 1 | positive |
| ENSG00000259330 | INAFM2 | 1 | positive |
| ENSG00000261371 | PECAM1 | 1 | positive |
| ENSG00000261787 | TCF24 | 1 | positive |
| ENSG00000263465 | SRSF8 | 1 | positive |
| ENSG00000266412 | NCOA4 | 1 | positive |
| ENSG00000268104 | SLC6A14 | 1 | positive |
| ENSG00000276045 | ORAI1 | 1 | positive |
| ENSG00000281991 | TMEM265 | 1 | positive |
| ENSG00000000457 | SCYL3 | 1 | negative |
| ENSG00000000971 | CFH | 1 | negative |
| ENSG00000001630 | CYP51A1 | 1 | negative |
| ENSG00000002587 | HS3ST1 | 1 | negative |
| ENSG00000003393 | ALS2 | 1 | negative |
| ENSG00000004534 | RBM6 | 1 | negative |
| ENSG00000005075 | POLR2J | 1 | negative |
| ENSG00000005884 | ITGA3 | 1 | negative |
| ENSG00000005893 | LAMP2 | 1 | negative |
| ENSG00000005981 | ASB4 | 1 | negative |
| ENSG00000006015 | REX1BD | 1 | negative |
| ENSG00000006125 | AP2B1 | 1 | negative |
| ENSG00000006625 | GGCT | 1 | negative |
| ENSG00000006756 | ARSD | 1 | negative |
| ENSG00000006837 | CDKL3 | 1 | negative |
| ENSG00000007255 | TRAPPC6A | 1 | negative |
| ENSG00000007384 | RHBDF1 | 1 | negative |
| ENSG00000007392 | LUC7L | 1 | negative |
| ENSG00000008018 | PSMB1 | 1 | negative |
| ENSG00000008196 | TFAP2B | 1 | negative |
| ENSG00000008294 | SPAG9 | 1 | negative |
| ENSG00000009413 | REV3L | 1 | negative |
| ENSG00000009780 | FAM76A | 1 | negative |
| ENSG00000009830 | POMT2 | 1 | negative |
| ENSG00000010310 | GIPR | 1 | negative |
| ENSG00000010404 | IDS | 1 | negative |
| ENSG00000010539 | ZNF200 | 1 | negative |
| ENSG00000012660 | ELOVL5 | 1 | negative |
| ENSG00000012963 | UBR7 | 1 | negative |
| ENSG00000014138 | POLA2 | 1 | negative |
| ENSG00000014641 | MDH1 | 1 | negative |
| ENSG00000015568 | RGPD5 | 1 | negative |
| ENSG00000016864 | GLT8D1 | 1 | negative |
| ENSG00000018610 | CXorf56 | 1 | negative |
| ENSG00000021300 | PLEKHB1 | 1 | negative |
| ENSG00000021574 | SPAST | 1 | negative |
| ENSG00000022277 | RTF2 | 1 | negative |
| ENSG00000023228 | NDUFS1 | 1 | negative |
| ENSG00000027075 | PRKCH | 1 | negative |
| ENSG00000028203 | VEZT | 1 | negative |
| ENSG00000029363 | BCLAF1 | 1 | negative |
| ENSG00000029993 | HMGB3 | 1 | negative |
| ENSG00000030066 | NUP160 | 1 | negative |
| ENSG00000033030 | ZCCHC8 | 1 | negative |
| ENSG00000033122 | LRRC7 | 1 | negative |
| ENSG00000035862 | TIMP2 | 1 | negative |
| ENSG00000035928 | RFC1 | 1 | negative |
| ENSG00000036530 | CYP46A1 | 1 | negative |
| ENSG00000036549 | AC118549.1 | 1 | negative |
| ENSG00000038219 | BOD1L1 | 1 | negative |
| ENSG00000042429 | MED17 | 1 | negative |
| ENSG00000042493 | CAPG | 1 | negative |
| ENSG00000046604 | DSG2 | 1 | negative |
| ENSG00000047249 | ATP6V1H | 1 | negative |
| ENSG00000047849 | MAP4 | 1 | negative |
| ENSG00000048540 | LMO3 | 1 | negative |
| ENSG00000048544 | MRPS10 | 1 | negative |
| ENSG00000048991 | R3HDM1 | 1 | negative |
| ENSG00000049283 | EPN3 | 1 | negative |
| ENSG00000049883 | PTCD2 | 1 | negative |
| ENSG00000050426 | LETMD1 | 1 | negative |
| ENSG00000050438 | SLC4A8 | 1 | negative |
| ENSG00000051341 | POLQ | 1 | negative |
| ENSG00000052126 | PLEKHA5 | 1 | negative |
| ENSG00000053254 | FOXN3 | 1 | negative |
| ENSG00000053372 | MRTO4 | 1 | negative |
| ENSG00000054148 | PHPT1 | 1 | negative |
| ENSG00000054654 | SYNE2 | 1 | negative |
| ENSG00000055732 | MCOLN3 | 1 | negative |
| ENSG00000056050 | HPF1 | 1 | negative |
| ENSG00000057935 | MTA3 | 1 | negative |
| ENSG00000062485 | CS | 1 | negative |
| ENSG00000063180 | CA11 | 1 | negative |
| ENSG00000063978 | RNF4 | 1 | negative |
| ENSG00000064042 | LIMCH1 | 1 | negative |
| ENSG00000064651 | SLC12A2 | 1 | negative |
| ENSG00000064933 | PMS1 | 1 | negative |
| ENSG00000064989 | CALCRL | 1 | negative |
| ENSG00000065054 | SLC9A3R2 | 1 | negative |
| ENSG00000065150 | IPO5 | 1 | negative |
| ENSG00000065361 | ERBB3 | 1 | negative |
| ENSG00000065548 | ZC3H15 | 1 | negative |
| ENSG00000065621 | GSTO2 | 1 | negative |
| ENSG00000065883 | CDK13 | 1 | negative |
| ENSG00000066056 | TIE1 | 1 | negative |
| ENSG00000067704 | IARS2 | 1 | negative |
| ENSG00000067798 | NAV3 | 1 | negative |
| ENSG00000068024 | HDAC4 | 1 | negative |
| ENSG00000068137 | PLEKHH3 | 1 | negative |
| ENSG00000068745 | IP6K2 | 1 | negative |
| ENSG00000069275 | NUCKS1 | 1 | negative |
| ENSG00000069812 | HES2 | 1 | negative |
| ENSG00000069869 | NEDD4 | 1 | negative |
| ENSG00000070081 | NUCB2 | 1 | negative |
| ENSG00000070087 | PFN2 | 1 | negative |
| ENSG00000070501 | POLB | 1 | negative |
| ENSG00000070814 | TCOF1 | 1 | negative |
| ENSG00000071553 | ATP6AP1 | 1 | negative |
| ENSG00000071575 | TRIB2 | 1 | negative |
| ENSG00000072133 | RPS6KA6 | 1 | negative |
| ENSG00000072134 | EPN2 | 1 | negative |
| ENSG00000072501 | SMC1A | 1 | negative |
| ENSG00000072786 | STK10 | 1 | negative |
| ENSG00000073584 | SMARCE1 | 1 | negative |
| ENSG00000073605 | GSDMB | 1 | negative |
| ENSG00000074054 | CLASP1 | 1 | negative |
| ENSG00000074071 | MRPS34 | 1 | negative |
| ENSG00000074657 | ZNF532 | 1 | negative |
| ENSG00000075218 | GTSE1 | 1 | negative |
| ENSG00000075234 | TTC38 | 1 | negative |
| ENSG00000075239 | ACAT1 | 1 | negative |
| ENSG00000075336 | TIMM21 | 1 | negative |
| ENSG00000075391 | RASAL2 | 1 | negative |
| ENSG00000075407 | ZNF37A | 1 | negative |
| ENSG00000075914 | EXOSC7 | 1 | negative |
| ENSG00000076258 | FMO4 | 1 | negative |
| ENSG00000076554 | TPD52 | 1 | negative |
| ENSG00000076555 | ACACB | 1 | negative |
| ENSG00000077232 | DNAJC10 | 1 | negative |
| ENSG00000077380 | DYNC1I2 | 1 | negative |
| ENSG00000077782 | FGFR1 | 1 | negative |
| ENSG00000078043 | PIAS2 | 1 | negative |
| ENSG00000078699 | CBFA2T2 | 1 | negative |
| ENSG00000079112 | CDH17 | 1 | negative |
| ENSG00000079150 | FKBP7 | 1 | negative |
| ENSG00000079462 | PAFAH1B3 | 1 | negative |
| ENSG00000079931 | MOXD1 | 1 | negative |
| ENSG00000080371 | RAB21 | 1 | negative |
| ENSG00000080986 | NDC80 | 1 | negative |
| ENSG00000081665 | ZNF506 | 1 | negative |
| ENSG00000081760 | AACS | 1 | negative |
| ENSG00000081870 | HSPB11 | 1 | negative |
| ENSG00000081923 | ATP8B1 | 1 | negative |
| ENSG00000082153 | BZW1 | 1 | negative |
| ENSG00000082512 | TRAF5 | 1 | negative |
| ENSG00000082805 | ERC1 | 1 | negative |
| ENSG00000083099 | LYRM2 | 1 | negative |
| ENSG00000083290 | ULK2 | 1 | negative |
| ENSG00000083807 | SLC27A5 | 1 | negative |
| ENSG00000083838 | ZNF446 | 1 | negative |
| ENSG00000084073 | ZMPSTE24 | 1 | negative |
| ENSG00000084463 | WBP11 | 1 | negative |
| ENSG00000085063 | CD59 | 1 | negative |
| ENSG00000085276 | MECOM | 1 | negative |
| ENSG00000085415 | SEH1L | 1 | negative |
| ENSG00000086062 | B4GALT1 | 1 | negative |
| ENSG00000086504 | MRPL28 | 1 | negative |
| ENSG00000086848 | ALG9 | 1 | negative |
| ENSG00000087266 | SH3BP2 | 1 | negative |
| ENSG00000087299 | L2HGDH | 1 | negative |
| ENSG00000087365 | SF3B2 | 1 | negative |
| ENSG00000087884 | AAMDC | 1 | negative |
| ENSG00000087995 | METTL2A | 1 | negative |
| ENSG00000088179 | PTPN4 | 1 | negative |
| ENSG00000088205 | DDX18 | 1 | negative |
| ENSG00000088808 | PPP1R13B | 1 | negative |
| ENSG00000088930 | XRN2 | 1 | negative |
| ENSG00000089009 | RPL6 | 1 | negative |
| ENSG00000089022 | MAPKAPK5 | 1 | negative |
| ENSG00000089041 | P2RX7 | 1 | negative |
| ENSG00000089091 | DZANK1 | 1 | negative |
| ENSG00000089157 | RPLP0 | 1 | negative |
| ENSG00000089248 | ERP29 | 1 | negative |
| ENSG00000089693 | MLF2 | 1 | negative |
| ENSG00000090060 | PAPOLA | 1 | negative |
| ENSG00000090487 | SPG21 | 1 | negative |
| ENSG00000090861 | AARS | 1 | negative |
| ENSG00000091181 | IL5RA | 1 | negative |
| ENSG00000091409 | ITGA6 | 1 | negative |
| ENSG00000091536 | MYO15A | 1 | negative |
| ENSG00000091651 | ORC6 | 1 | negative |
| ENSG00000092201 | SUPT16H | 1 | negative |
| ENSG00000092621 | PHGDH | 1 | negative |
| ENSG00000092820 | EZR | 1 | negative |
| ENSG00000092964 | DPYSL2 | 1 | negative |
| ENSG00000093217 | XYLB | 1 | negative |
| ENSG00000094916 | CBX5 | 1 | negative |
| ENSG00000095002 | MSH2 | 1 | negative |
| ENSG00000095380 | NANS | 1 | negative |
| ENSG00000095906 | NUBP2 | 1 | negative |
| ENSG00000096384 | HSP90AB1 | 1 | negative |
| ENSG00000097021 | ACOT7 | 1 | negative |
| ENSG00000099385 | BCL7C | 1 | negative |
| ENSG00000099795 | NDUFB7 | 1 | negative |
| ENSG00000099797 | TECR | 1 | negative |
| ENSG00000099840 | IZUMO4 | 1 | negative |
| ENSG00000099864 | PALM | 1 | negative |
| ENSG00000099968 | BCL2L13 | 1 | negative |
| ENSG00000099994 | SUSD2 | 1 | negative |
| ENSG00000099998 | GGT5 | 1 | negative |
| ENSG00000100206 | DMC1 | 1 | negative |
| ENSG00000100219 | XBP1 | 1 | negative |
| ENSG00000100253 | MIOX | 1 | negative |
| ENSG00000100299 | ARSA | 1 | negative |
| ENSG00000100353 | EIF3D | 1 | negative |
| ENSG00000100461 | RBM23 | 1 | negative |
| ENSG00000100522 | GNPNAT1 | 1 | negative |
| ENSG00000100578 | KIAA0586 | 1 | negative |
| ENSG00000100632 | ERH | 1 | negative |
| ENSG00000100644 | HIF1A | 1 | negative |
| ENSG00000100714 | MTHFD1 | 1 | negative |
| ENSG00000100722 | ZC3H14 | 1 | negative |
| ENSG00000100731 | PCNX1 | 1 | negative |
| ENSG00000100814 | CCNB1IP1 | 1 | negative |
| ENSG00000100865 | CINP | 1 | negative |
| ENSG00000100867 | DHRS2 | 1 | negative |
| ENSG00000100888 | CHD8 | 1 | negative |
| ENSG00000100890 | KIAA0391 | 1 | negative |
| ENSG00000100934 | SEC23A | 1 | negative |
| ENSG00000100941 | PNN | 1 | negative |
| ENSG00000101003 | GINS1 | 1 | negative |
| ENSG00000101019 | UQCC1 | 1 | negative |
| ENSG00000101084 | C20orf24 | 1 | negative |
| ENSG00000101126 | ADNP | 1 | negative |
| ENSG00000101138 | CSTF1 | 1 | negative |
| ENSG00000101158 | NELFCD | 1 | negative |
| ENSG00000101193 | GID8 | 1 | negative |
| ENSG00000101224 | CDC25B | 1 | negative |
| ENSG00000101266 | CSNK2A1 | 1 | negative |
| ENSG00000101294 | HM13 | 1 | negative |
| ENSG00000101346 | POFUT1 | 1 | negative |
| ENSG00000101350 | KIF3B | 1 | negative |
| ENSG00000101361 | NOP56 | 1 | negative |
| ENSG00000101367 | MAPRE1 | 1 | negative |
| ENSG00000101417 | PXMP4 | 1 | negative |
| ENSG00000101474 | APMAP | 1 | negative |
| ENSG00000101624 | CEP76 | 1 | negative |
| ENSG00000101812 | H2BFM | 1 | negative |
| ENSG00000101882 | NKAP | 1 | negative |
| ENSG00000101966 | XIAP | 1 | negative |
| ENSG00000102024 | PLS3 | 1 | negative |
| ENSG00000102144 | PGK1 | 1 | negative |
| ENSG00000102158 | MAGT1 | 1 | negative |
| ENSG00000102271 | KLHL4 | 1 | negative |
| ENSG00000102309 | PIN4 | 1 | negative |
| ENSG00000102349 | KLF8 | 1 | negative |
| ENSG00000102595 | UGGT2 | 1 | negative |
| ENSG00000102786 | INTS6 | 1 | negative |
| ENSG00000102805 | CLN5 | 1 | negative |
| ENSG00000102882 | MAPK3 | 1 | negative |
| ENSG00000102934 | PLLP | 1 | negative |
| ENSG00000102974 | CTCF | 1 | negative |
| ENSG00000103021 | CCDC113 | 1 | negative |
| ENSG00000103145 | HCFC1R1 | 1 | negative |
| ENSG00000103152 | MPG | 1 | negative |
| ENSG00000103197 | TSC2 | 1 | negative |
| ENSG00000103199 | ZNF500 | 1 | negative |
| ENSG00000103275 | UBE2I | 1 | negative |
| ENSG00000103319 | EEF2K | 1 | negative |
| ENSG00000103351 | CLUAP1 | 1 | negative |
| ENSG00000103356 | EARS2 | 1 | negative |
| ENSG00000103415 | HMOX2 | 1 | negative |
| ENSG00000103423 | DNAJA3 | 1 | negative |
| ENSG00000103544 | VPS35L | 1 | negative |
| ENSG00000103707 | MTFMT | 1 | negative |
| ENSG00000103710 | RASL12 | 1 | negative |
| ENSG00000103995 | CEP152 | 1 | negative |
| ENSG00000104154 | SLC30A4 | 1 | negative |
| ENSG00000104290 | FZD3 | 1 | negative |
| ENSG00000104325 | DECR1 | 1 | negative |
| ENSG00000104368 | PLAT | 1 | negative |
| ENSG00000104412 | EMC2 | 1 | negative |
| ENSG00000104442 | ARMC1 | 1 | negative |
| ENSG00000104447 | TRPS1 | 1 | negative |
| ENSG00000104490 | NCALD | 1 | negative |
| ENSG00000104728 | ARHGEF10 | 1 | negative |
| ENSG00000104804 | TULP2 | 1 | negative |
| ENSG00000104814 | MAP4K1 | 1 | negative |
| ENSG00000104881 | PPP1R13L | 1 | negative |
| ENSG00000104885 | DOT1L | 1 | negative |
| ENSG00000104889 | RNASEH2A | 1 | negative |
| ENSG00000104979 | C19orf53 | 1 | negative |
| ENSG00000104980 | TIMM44 | 1 | negative |
| ENSG00000105143 | SLC1A6 | 1 | negative |
| ENSG00000105197 | TIMM50 | 1 | negative |
| ENSG00000105221 | AKT2 | 1 | negative |
| ENSG00000105479 | CCDC114 | 1 | negative |
| ENSG00000105519 | CAPS | 1 | negative |
| ENSG00000105552 | BCAT2 | 1 | negative |
| ENSG00000105642 | KCNN1 | 1 | negative |
| ENSG00000105647 | PIK3R2 | 1 | negative |
| ENSG00000105649 | RAB3A | 1 | negative |
| ENSG00000105717 | PBX4 | 1 | negative |
| ENSG00000105750 | ZNF85 | 1 | negative |
| ENSG00000105792 | CFAP69 | 1 | negative |
| ENSG00000105856 | HBP1 | 1 | negative |
| ENSG00000106003 | LFNG | 1 | negative |
| ENSG00000106052 | TAX1BP1 | 1 | negative |
| ENSG00000106066 | CPVL | 1 | negative |
| ENSG00000106125 | MINDY4 | 1 | negative |
| ENSG00000106261 | ZKSCAN1 | 1 | negative |
| ENSG00000106305 | AIMP2 | 1 | negative |
| ENSG00000106344 | RBM28 | 1 | negative |
| ENSG00000106348 | IMPDH1 | 1 | negative |
| ENSG00000106460 | TMEM106B | 1 | negative |
| ENSG00000106537 | TSPAN13 | 1 | negative |
| ENSG00000106554 | CHCHD3 | 1 | negative |
| ENSG00000106603 | COA1 | 1 | negative |
| ENSG00000106608 | URGCP | 1 | negative |
| ENSG00000106617 | PRKAG2 | 1 | negative |
| ENSG00000106638 | TBL2 | 1 | negative |
| ENSG00000106686 | SPATA6L | 1 | negative |
| ENSG00000106803 | SEC61B | 1 | negative |
| ENSG00000106853 | PTGR1 | 1 | negative |
| ENSG00000106992 | AK1 | 1 | negative |
| ENSG00000107159 | CA9 | 1 | negative |
| ENSG00000107223 | EDF1 | 1 | negative |
| ENSG00000107371 | EXOSC3 | 1 | negative |
| ENSG00000107485 | GATA3 | 1 | negative |
| ENSG00000107554 | DNMBP | 1 | negative |
| ENSG00000107643 | MAPK8 | 1 | negative |
| ENSG00000107779 | BMPR1A | 1 | negative |
| ENSG00000107819 | SFXN3 | 1 | negative |
| ENSG00000107897 | ACBD5 | 1 | negative |
| ENSG00000107951 | MTPAP | 1 | negative |
| ENSG00000108064 | TFAM | 1 | negative |
| ENSG00000108106 | UBE2S | 1 | negative |
| ENSG00000108384 | RAD51C | 1 | negative |
| ENSG00000108439 | PNPO | 1 | negative |
| ENSG00000108468 | CBX1 | 1 | negative |
| ENSG00000108559 | NUP88 | 1 | negative |
| ENSG00000108578 | BLMH | 1 | negative |
| ENSG00000108582 | CPD | 1 | negative |
| ENSG00000108587 | GOSR1 | 1 | negative |
| ENSG00000108651 | UTP6 | 1 | negative |
| ENSG00000108654 | DDX5 | 1 | negative |
| ENSG00000108829 | LRRC59 | 1 | negative |
| ENSG00000109063 | MYH3 | 1 | negative |
| ENSG00000109133 | TMEM33 | 1 | negative |
| ENSG00000109189 | USP46 | 1 | negative |
| ENSG00000109193 | SULT1E1 | 1 | negative |
| ENSG00000109339 | MAPK10 | 1 | negative |
| ENSG00000109390 | NDUFC1 | 1 | negative |
| ENSG00000109458 | GAB1 | 1 | negative |
| ENSG00000109472 | CPE | 1 | negative |
| ENSG00000109475 | RPL34 | 1 | negative |
| ENSG00000109606 | DHX15 | 1 | negative |
| ENSG00000109618 | SEPSECS | 1 | negative |
| ENSG00000109743 | BST1 | 1 | negative |
| ENSG00000110025 | SNX15 | 1 | negative |
| ENSG00000110435 | PDHX | 1 | negative |
| ENSG00000110492 | MDK | 1 | negative |
| ENSG00000110583 | NAA40 | 1 | negative |
| ENSG00000110660 | SLC35F2 | 1 | negative |
| ENSG00000110696 | C11orf58 | 1 | negative |
| ENSG00000110713 | NUP98 | 1 | negative |
| ENSG00000110841 | PPFIBP1 | 1 | negative |
| ENSG00000110871 | COQ5 | 1 | negative |
| ENSG00000110888 | CAPRIN2 | 1 | negative |
| ENSG00000110911 | SLC11A2 | 1 | negative |
| ENSG00000110931 | CAMKK2 | 1 | negative |
| ENSG00000111011 | RSRC2 | 1 | negative |
| ENSG00000111142 | METAP2 | 1 | negative |
| ENSG00000111206 | FOXM1 | 1 | negative |
| ENSG00000111261 | MANSC1 | 1 | negative |
| ENSG00000111275 | ALDH2 | 1 | negative |
| ENSG00000111300 | NAA25 | 1 | negative |
| ENSG00000111319 | SCNN1A | 1 | negative |
| ENSG00000111341 | MGP | 1 | negative |
| ENSG00000111364 | DDX55 | 1 | negative |
| ENSG00000111371 | SLC38A1 | 1 | negative |
| ENSG00000111490 | TBC1D30 | 1 | negative |
| ENSG00000111665 | CDCA3 | 1 | negative |
| ENSG00000111669 | TPI1 | 1 | negative |
| ENSG00000111707 | SUDS3 | 1 | negative |
| ENSG00000111775 | COX6A1 | 1 | negative |
| ENSG00000111816 | FRK | 1 | negative |
| ENSG00000111843 | TMEM14C | 1 | negative |
| ENSG00000111875 | ASF1A | 1 | negative |
| ENSG00000112033 | PPARD | 1 | negative |
| ENSG00000112139 | MDGA1 | 1 | negative |
| ENSG00000112276 | BVES | 1 | negative |
| ENSG00000112308 | C6orf62 | 1 | negative |
| ENSG00000112378 | PERP | 1 | negative |
| ENSG00000112425 | EPM2A | 1 | negative |
| ENSG00000112473 | SLC39A7 | 1 | negative |
| ENSG00000112514 | CUTA | 1 | negative |
| ENSG00000112983 | BRD8 | 1 | negative |
| ENSG00000113273 | ARSB | 1 | negative |
| ENSG00000113558 | SKP1 | 1 | negative |
| ENSG00000113719 | ERGIC1 | 1 | negative |
| ENSG00000113790 | EHHADH | 1 | negative |
| ENSG00000114021 | NIT2 | 1 | negative |
| ENSG00000114054 | PCCB | 1 | negative |
| ENSG00000114115 | RBP1 | 1 | negative |
| ENSG00000114125 | RNF7 | 1 | negative |
| ENSG00000114126 | TFDP2 | 1 | negative |
| ENSG00000114315 | HES1 | 1 | negative |
| ENSG00000114346 | ECT2 | 1 | negative |
| ENSG00000114503 | NCBP2 | 1 | negative |
| ENSG00000114656 | KIAA1257 | 1 | negative |
| ENSG00000114686 | MRPL3 | 1 | negative |
| ENSG00000114742 | WDR48 | 1 | negative |
| ENSG00000114796 | KLHL24 | 1 | negative |
| ENSG00000114861 | FOXP1 | 1 | negative |
| ENSG00000114999 | TTL | 1 | negative |
| ENSG00000115042 | FAHD2A | 1 | negative |
| ENSG00000115109 | EPB41L5 | 1 | negative |
| ENSG00000115128 | SF3B6 | 1 | negative |
| ENSG00000115221 | ITGB6 | 1 | negative |
| ENSG00000115239 | ASB3 | 1 | negative |
| ENSG00000115241 | PPM1G | 1 | negative |
| ENSG00000115307 | AUP1 | 1 | negative |
| ENSG00000115310 | RTN4 | 1 | negative |
| ENSG00000115464 | USP34 | 1 | negative |
| ENSG00000115507 | OTX1 | 1 | negative |
| ENSG00000115539 | PDCL3 | 1 | negative |
| ENSG00000115648 | MLPH | 1 | negative |
| ENSG00000115827 | DCAF17 | 1 | negative |
| ENSG00000115884 | SDC1 | 1 | negative |
| ENSG00000115902 | SLC1A4 | 1 | negative |
| ENSG00000115947 | ORC4 | 1 | negative |
| ENSG00000116062 | MSH6 | 1 | negative |
| ENSG00000116106 | EPHA4 | 1 | negative |
| ENSG00000116171 | SCP2 | 1 | negative |
| ENSG00000116191 | RALGPS2 | 1 | negative |
| ENSG00000116209 | TMEM59 | 1 | negative |
| ENSG00000116560 | SFPQ | 1 | negative |
| ENSG00000116641 | DOCK7 | 1 | negative |
| ENSG00000117122 | MFAP2 | 1 | negative |
| ENSG00000117335 | CD46 | 1 | negative |
| ENSG00000117360 | PRPF3 | 1 | negative |
| ENSG00000117410 | ATP6V0B | 1 | negative |
| ENSG00000117448 | AKR1A1 | 1 | negative |
| ENSG00000117472 | TSPAN1 | 1 | negative |
| ENSG00000117477 | CCDC181 | 1 | negative |
| ENSG00000117620 | SLC35A3 | 1 | negative |
| ENSG00000117632 | STMN1 | 1 | negative |
| ENSG00000117650 | NEK2 | 1 | negative |
| ENSG00000117724 | CENPF | 1 | negative |
| ENSG00000117791 | 2-Mar | 1 | negative |
| ENSG00000117877 | CD3EAP | 1 | negative |
| ENSG00000117906 | RCN2 | 1 | negative |
| ENSG00000117984 | CTSD | 1 | negative |
| ENSG00000118096 | IFT46 | 1 | negative |
| ENSG00000118137 | APOA1 | 1 | negative |
| ENSG00000118181 | RPS25 | 1 | negative |
| ENSG00000118193 | KIF14 | 1 | negative |
| ENSG00000118482 | PHF3 | 1 | negative |
| ENSG00000118600 | RXYLT1 | 1 | negative |
| ENSG00000118640 | VAMP8 | 1 | negative |
| ENSG00000118705 | RPN2 | 1 | negative |
| ENSG00000118873 | RAB3GAP2 | 1 | negative |
| ENSG00000119004 | CYP20A1 | 1 | negative |
| ENSG00000119335 | SET | 1 | negative |
| ENSG00000119508 | NR4A3 | 1 | negative |
| ENSG00000119509 | INVS | 1 | negative |
| ENSG00000119537 | KDSR | 1 | negative |
| ENSG00000119616 | FCF1 | 1 | negative |
| ENSG00000119655 | NPC2 | 1 | negative |
| ENSG00000119707 | RBM25 | 1 | negative |
| ENSG00000119711 | ALDH6A1 | 1 | negative |
| ENSG00000119723 | COQ6 | 1 | negative |
| ENSG00000119777 | TMEM214 | 1 | negative |
| ENSG00000119787 | ATL2 | 1 | negative |
| ENSG00000119820 | YIPF4 | 1 | negative |
| ENSG00000119878 | CRIPT | 1 | negative |
| ENSG00000119888 | EPCAM | 1 | negative |
| ENSG00000119906 | SLF2 | 1 | negative |
| ENSG00000119969 | HELLS | 1 | negative |
| ENSG00000119977 | TCTN3 | 1 | negative |
| ENSG00000120129 | DUSP1 | 1 | negative |
| ENSG00000120262 | CCDC170 | 1 | negative |
| ENSG00000120314 | WDR55 | 1 | negative |
| ENSG00000120708 | TGFBI | 1 | negative |
| ENSG00000120805 | ARL1 | 1 | negative |
| ENSG00000120875 | DUSP4 | 1 | negative |
| ENSG00000120907 | ADRA1A | 1 | negative |
| ENSG00000121064 | SCPEP1 | 1 | negative |
| ENSG00000121289 | CEP89 | 1 | negative |
| ENSG00000121316 | PLBD1 | 1 | negative |
| ENSG00000121361 | KCNJ8 | 1 | negative |
| ENSG00000121864 | ZNF639 | 1 | negative |
| ENSG00000121897 | LIAS | 1 | negative |
| ENSG00000121940 | CLCC1 | 1 | negative |
| ENSG00000121957 | GPSM2 | 1 | negative |
| ENSG00000121966 | CXCR4 | 1 | negative |
| ENSG00000122026 | RPL21 | 1 | negative |
| ENSG00000122203 | KIAA1191 | 1 | negative |
| ENSG00000122378 | FAM213A | 1 | negative |
| ENSG00000122406 | RPL5 | 1 | negative |
| ENSG00000122417 | ODF2L | 1 | negative |
| ENSG00000122484 | RPAP2 | 1 | negative |
| ENSG00000122545 | 7-Sep | 1 | negative |
| ENSG00000122566 | HNRNPA2B1 | 1 | negative |
| ENSG00000122642 | FKBP9 | 1 | negative |
| ENSG00000122674 | CCZ1 | 1 | negative |
| ENSG00000122692 | SMU1 | 1 | negative |
| ENSG00000122778 | KIAA1549 | 1 | negative |
| ENSG00000122966 | CIT | 1 | negative |
| ENSG00000123130 | ACOT9 | 1 | negative |
| ENSG00000123352 | SPATS2 | 1 | negative |
| ENSG00000123473 | STIL | 1 | negative |
| ENSG00000123562 | MORF4L2 | 1 | negative |
| ENSG00000123636 | BAZ2B | 1 | negative |
| ENSG00000124006 | OBSL1 | 1 | negative |
| ENSG00000124098 | FAM210B | 1 | negative |
| ENSG00000124155 | PIGT | 1 | negative |
| ENSG00000124171 | PARD6B | 1 | negative |
| ENSG00000124177 | CHD6 | 1 | negative |
| ENSG00000124193 | SRSF6 | 1 | negative |
| ENSG00000124214 | STAU1 | 1 | negative |
| ENSG00000124217 | MOCS3 | 1 | negative |
| ENSG00000124225 | PMEPA1 | 1 | negative |
| ENSG00000124260 | MAGEA10 | 1 | negative |
| ENSG00000124374 | PAIP2B | 1 | negative |
| ENSG00000124608 | AARS2 | 1 | negative |
| ENSG00000124614 | RPS10 | 1 | negative |
| ENSG00000124659 | TBCC | 1 | negative |
| ENSG00000124664 | SPDEF | 1 | negative |
| ENSG00000124702 | KLHDC3 | 1 | negative |
| ENSG00000124831 | LRRFIP1 | 1 | negative |
| ENSG00000125166 | GOT2 | 1 | negative |
| ENSG00000125356 | NDUFA1 | 1 | negative |
| ENSG00000125378 | BMP4 | 1 | negative |
| ENSG00000125534 | PPDPF | 1 | negative |
| ENSG00000125691 | RPL23 | 1 | negative |
| ENSG00000125731 | SH2D3A | 1 | negative |
| ENSG00000125746 | EML2 | 1 | negative |
| ENSG00000125835 | SNRPB | 1 | negative |
| ENSG00000125851 | PCSK2 | 1 | negative |
| ENSG00000125885 | MCM8 | 1 | negative |
| ENSG00000125968 | ID1 | 1 | negative |
| ENSG00000125991 | ERGIC3 | 1 | negative |
| ENSG00000125995 | ROMO1 | 1 | negative |
| ENSG00000125999 | BPIFB1 | 1 | negative |
| ENSG00000126001 | CEP250 | 1 | negative |
| ENSG00000126456 | IRF3 | 1 | negative |
| ENSG00000126653 | NSRP1 | 1 | negative |
| ENSG00000126698 | DNAJC8 | 1 | negative |
| ENSG00000126756 | UXT | 1 | negative |
| ENSG00000127022 | CANX | 1 | negative |
| ENSG00000127184 | COX7C | 1 | negative |
| ENSG00000127463 | EMC1 | 1 | negative |
| ENSG00000127540 | UQCR11 | 1 | negative |
| ENSG00000128059 | PPAT | 1 | negative |
| ENSG00000128463 | EMC4 | 1 | negative |
| ENSG00000128524 | ATP6V1F | 1 | negative |
| ENSG00000128581 | IFT22 | 1 | negative |
| ENSG00000128617 | OPN1SW | 1 | negative |
| ENSG00000128805 | ARHGAP22 | 1 | negative |
| ENSG00000128829 | EIF2AK4 | 1 | negative |
| ENSG00000128833 | MYO5C | 1 | negative |
| ENSG00000128928 | IVD | 1 | negative |
| ENSG00000128973 | CLN6 | 1 | negative |
| ENSG00000129009 | ISLR | 1 | negative |
| ENSG00000129235 | TXNDC17 | 1 | negative |
| ENSG00000129295 | LRRC6 | 1 | negative |
| ENSG00000129317 | PUS7L | 1 | negative |
| ENSG00000129351 | ILF3 | 1 | negative |
| ENSG00000129562 | DAD1 | 1 | negative |
| ENSG00000130150 | MOSPD2 | 1 | negative |
| ENSG00000130175 | PRKCSH | 1 | negative |
| ENSG00000130244 | FAM98C | 1 | negative |
| ENSG00000130304 | SLC27A1 | 1 | negative |
| ENSG00000130309 | COLGALT1 | 1 | negative |
| ENSG00000130332 | LSM7 | 1 | negative |
| ENSG00000130338 | TULP4 | 1 | negative |
| ENSG00000130348 | QRSL1 | 1 | negative |
| ENSG00000130349 | C6orf203 | 1 | negative |
| ENSG00000130363 | RSPH3 | 1 | negative |
| ENSG00000130402 | ACTN4 | 1 | negative |
| ENSG00000130413 | STK33 | 1 | negative |
| ENSG00000130414 | NDUFA10 | 1 | negative |
| ENSG00000130529 | TRPM4 | 1 | negative |
| ENSG00000130561 | SAG | 1 | negative |
| ENSG00000130695 | CEP85 | 1 | negative |
| ENSG00000130726 | TRIM28 | 1 | negative |
| ENSG00000130770 | ATP5IF1 | 1 | negative |
| ENSG00000130844 | ZNF331 | 1 | negative |
| ENSG00000130921 | C12orf65 | 1 | negative |
| ENSG00000131019 | ULBP3 | 1 | negative |
| ENSG00000131043 | AAR2 | 1 | negative |
| ENSG00000131044 | TTLL9 | 1 | negative |
| ENSG00000131089 | ARHGEF9 | 1 | negative |
| ENSG00000131127 | ZNF141 | 1 | negative |
| ENSG00000131143 | COX4I1 | 1 | negative |
| ENSG00000131368 | MRPS25 | 1 | negative |
| ENSG00000131374 | TBC1D5 | 1 | negative |
| ENSG00000131475 | VPS25 | 1 | negative |
| ENSG00000131508 | UBE2D2 | 1 | negative |
| ENSG00000131652 | THOC6 | 1 | negative |
| ENSG00000131711 | MAP1B | 1 | negative |
| ENSG00000131746 | TNS4 | 1 | negative |
| ENSG00000131747 | TOP2A | 1 | negative |
| ENSG00000131771 | PPP1R1B | 1 | negative |
| ENSG00000131844 | MCCC2 | 1 | negative |
| ENSG00000131848 | ZSCAN5A | 1 | negative |
| ENSG00000131871 | SELENOS | 1 | negative |
| ENSG00000131876 | SNRPA1 | 1 | negative |
| ENSG00000132002 | DNAJB1 | 1 | negative |
| ENSG00000132141 | CCT6B | 1 | negative |
| ENSG00000132300 | PTCD3 | 1 | negative |
| ENSG00000132386 | SERPINF1 | 1 | negative |
| ENSG00000132464 | ENAM | 1 | negative |
| ENSG00000132470 | ITGB4 | 1 | negative |
| ENSG00000132507 | EIF5A | 1 | negative |
| ENSG00000132549 | VPS13B | 1 | negative |
| ENSG00000132570 | PCBD2 | 1 | negative |
| ENSG00000132676 | DAP3 | 1 | negative |
| ENSG00000132680 | KHDC4 | 1 | negative |
| ENSG00000132780 | NASP | 1 | negative |
| ENSG00000132801 | ZSWIM3 | 1 | negative |
| ENSG00000132823 | OSER1 | 1 | negative |
| ENSG00000133028 | SCO1 | 1 | negative |
| ENSG00000133067 | LGR6 | 1 | negative |
| ENSG00000133101 | CCNA1 | 1 | negative |
| ENSG00000133114 | GPALPP1 | 1 | negative |
| ENSG00000133131 | MORC4 | 1 | negative |
| ENSG00000133138 | TBC1D8B | 1 | negative |
| ENSG00000133142 | TCEAL4 | 1 | negative |
| ENSG00000133226 | SRRM1 | 1 | negative |
| ENSG00000133706 | LARS | 1 | negative |
| ENSG00000133812 | SBF2 | 1 | negative |
| ENSG00000133884 | DPF2 | 1 | negative |
| ENSG00000134001 | EIF2S1 | 1 | negative |
| ENSG00000134153 | EMC7 | 1 | negative |
| ENSG00000134215 | VAV3 | 1 | negative |
| ENSG00000134243 | SORT1 | 1 | negative |
| ENSG00000134283 | PPHLN1 | 1 | negative |
| ENSG00000134308 | YWHAQ | 1 | negative |
| ENSG00000134330 | IAH1 | 1 | negative |
| ENSG00000134440 | NARS | 1 | negative |
| ENSG00000134490 | TMEM241 | 1 | negative |
| ENSG00000134531 | EMP1 | 1 | negative |
| ENSG00000134590 | RTL8C | 1 | negative |
| ENSG00000134716 | CYP2J2 | 1 | negative |
| ENSG00000134748 | PRPF38A | 1 | negative |
| ENSG00000134905 | CARS2 | 1 | negative |
| ENSG00000134910 | STT3A | 1 | negative |
| ENSG00000135074 | ADAM19 | 1 | negative |
| ENSG00000135119 | RNFT2 | 1 | negative |
| ENSG00000135249 | RINT1 | 1 | negative |
| ENSG00000135299 | ANKRD6 | 1 | negative |
| ENSG00000135316 | SYNCRIP | 1 | negative |
| ENSG00000135333 | EPHA7 | 1 | negative |
| ENSG00000135378 | PRRG4 | 1 | negative |
| ENSG00000135452 | TSPAN31 | 1 | negative |
| ENSG00000135476 | ESPL1 | 1 | negative |
| ENSG00000135480 | KRT7 | 1 | negative |
| ENSG00000135486 | HNRNPA1 | 1 | negative |
| ENSG00000135720 | DYNC1LI2 | 1 | negative |
| ENSG00000135740 | SLC9A5 | 1 | negative |
| ENSG00000135842 | FAM129A | 1 | negative |
| ENSG00000135912 | TTLL4 | 1 | negative |
| ENSG00000135913 | USP37 | 1 | negative |
| ENSG00000135917 | SLC19A3 | 1 | negative |
| ENSG00000135966 | TGFBRAP1 | 1 | negative |
| ENSG00000135968 | GCC2 | 1 | negative |
| ENSG00000136068 | FLNB | 1 | negative |
| ENSG00000136146 | MED4 | 1 | negative |
| ENSG00000136267 | DGKB | 1 | negative |
| ENSG00000136436 | CALCOCO2 | 1 | negative |
| ENSG00000136504 | KAT7 | 1 | negative |
| ENSG00000136518 | ACTL6A | 1 | negative |
| ENSG00000136802 | LRRC8A | 1 | negative |
| ENSG00000136828 | RALGPS1 | 1 | negative |
| ENSG00000136861 | CDK5RAP2 | 1 | negative |
| ENSG00000136868 | SLC31A1 | 1 | negative |
| ENSG00000137124 | ALDH1B1 | 1 | negative |
| ENSG00000137133 | HINT2 | 1 | negative |
| ENSG00000137210 | TMEM14B | 1 | negative |
| ENSG00000137274 | BPHL | 1 | negative |
| ENSG00000137343 | ATAT1 | 1 | negative |
| ENSG00000137409 | MTCH1 | 1 | negative |
| ENSG00000137413 | TAF8 | 1 | negative |
| ENSG00000137497 | NUMA1 | 1 | negative |
| ENSG00000137500 | CCDC90B | 1 | negative |
| ENSG00000137522 | RNF121 | 1 | negative |
| ENSG00000137720 | C11orf1 | 1 | negative |
| ENSG00000137807 | KIF23 | 1 | negative |
| ENSG00000137814 | HAUS2 | 1 | negative |
| ENSG00000137842 | TMEM62 | 1 | negative |
| ENSG00000137868 | STRA6 | 1 | negative |
| ENSG00000137968 | SLC44A5 | 1 | negative |
| ENSG00000138035 | PNPT1 | 1 | negative |
| ENSG00000138085 | ATRAID | 1 | negative |
| ENSG00000138111 | MFSD13A | 1 | negative |
| ENSG00000138162 | TACC2 | 1 | negative |
| ENSG00000138175 | ARL3 | 1 | negative |
| ENSG00000138303 | ASCC1 | 1 | negative |
| ENSG00000138316 | ADAMTS14 | 1 | negative |
| ENSG00000138375 | SMARCAL1 | 1 | negative |
| ENSG00000138398 | PPIG | 1 | negative |
| ENSG00000138442 | WDR12 | 1 | negative |
| ENSG00000138448 | ITGAV | 1 | negative |
| ENSG00000138468 | SENP7 | 1 | negative |
| ENSG00000138617 | PARP16 | 1 | negative |
| ENSG00000138658 | ZGRF1 | 1 | negative |
| ENSG00000138760 | SCARB2 | 1 | negative |
| ENSG00000138771 | SHROOM3 | 1 | negative |
| ENSG00000138772 | ANXA3 | 1 | negative |
| ENSG00000138777 | PPA2 | 1 | negative |
| ENSG00000138794 | CASP6 | 1 | negative |
| ENSG00000138814 | PPP3CA | 1 | negative |
| ENSG00000138835 | RGS3 | 1 | negative |
| ENSG00000139116 | KIF21A | 1 | negative |
| ENSG00000139144 | PIK3C2G | 1 | negative |
| ENSG00000139168 | ZCRB1 | 1 | negative |
| ENSG00000139190 | VAMP1 | 1 | negative |
| ENSG00000139233 | LLPH | 1 | negative |
| ENSG00000139291 | TMEM19 | 1 | negative |
| ENSG00000139324 | TMTC3 | 1 | negative |
| ENSG00000139613 | SMARCC2 | 1 | negative |
| ENSG00000139624 | CERS5 | 1 | negative |
| ENSG00000139629 | GALNT6 | 1 | negative |
| ENSG00000139631 | CSAD | 1 | negative |
| ENSG00000139719 | VPS33A | 1 | negative |
| ENSG00000139734 | DIAPH3 | 1 | negative |
| ENSG00000139921 | TMX1 | 1 | negative |
| ENSG00000139974 | SLC38A6 | 1 | negative |
| ENSG00000140025 | EFCAB11 | 1 | negative |
| ENSG00000140043 | PTGR2 | 1 | negative |
| ENSG00000140297 | GCNT3 | 1 | negative |
| ENSG00000140319 | SRP14 | 1 | negative |
| ENSG00000140365 | COMMD4 | 1 | negative |
| ENSG00000140450 | ARRDC4 | 1 | negative |
| ENSG00000140451 | PIF1 | 1 | negative |
| ENSG00000140505 | CYP1A2 | 1 | negative |
| ENSG00000140545 | MFGE8 | 1 | negative |
| ENSG00000140612 | SEC11A | 1 | negative |
| ENSG00000140718 | FTO | 1 | negative |
| ENSG00000140832 | MARVELD3 | 1 | negative |
| ENSG00000140836 | ZFHX3 | 1 | negative |
| ENSG00000140950 | TLDC1 | 1 | negative |
| ENSG00000140983 | RHOT2 | 1 | negative |
| ENSG00000140988 | RPS2 | 1 | negative |
| ENSG00000141027 | NCOR1 | 1 | negative |
| ENSG00000141232 | TOB1 | 1 | negative |
| ENSG00000141252 | VPS53 | 1 | negative |
| ENSG00000141294 | LRRC46 | 1 | negative |
| ENSG00000141337 | ARSG | 1 | negative |
| ENSG00000141510 | TP53 | 1 | negative |
| ENSG00000141551 | CSNK1D | 1 | negative |
| ENSG00000141665 | FBXO15 | 1 | negative |
| ENSG00000141682 | PMAIP1 | 1 | negative |
| ENSG00000141736 | ERBB2 | 1 | negative |
| ENSG00000141741 | MIEN1 | 1 | negative |
| ENSG00000141753 | IGFBP4 | 1 | negative |
| ENSG00000141837 | CACNA1A | 1 | negative |
| ENSG00000141934 | PLPP2 | 1 | negative |
| ENSG00000141965 | FEM1A | 1 | negative |
| ENSG00000142149 | HUNK | 1 | negative |
| ENSG00000142166 | IFNAR1 | 1 | negative |
| ENSG00000142197 | DOPEY2 | 1 | negative |
| ENSG00000142230 | SAE1 | 1 | negative |
| ENSG00000142507 | PSMB6 | 1 | negative |
| ENSG00000142534 | RPS11 | 1 | negative |
| ENSG00000142556 | ZNF614 | 1 | negative |
| ENSG00000142621 | FHAD1 | 1 | negative |
| ENSG00000142687 | KIAA0319L | 1 | negative |
| ENSG00000142765 | SYTL1 | 1 | negative |
| ENSG00000143153 | ATP1B1 | 1 | negative |
| ENSG00000143156 | NME7 | 1 | negative |
| ENSG00000143198 | MGST3 | 1 | negative |
| ENSG00000143222 | UFC1 | 1 | negative |
| ENSG00000143252 | SDHC | 1 | negative |
| ENSG00000143314 | MRPL24 | 1 | negative |
| ENSG00000143319 | ISG20L2 | 1 | negative |
| ENSG00000143320 | CRABP2 | 1 | negative |
| ENSG00000143322 | ABL2 | 1 | negative |
| ENSG00000143374 | TARS2 | 1 | negative |
| ENSG00000143376 | SNX27 | 1 | negative |
| ENSG00000143387 | CTSK | 1 | negative |
| ENSG00000143398 | PIP5K1A | 1 | negative |
| ENSG00000143442 | POGZ | 1 | negative |
| ENSG00000143458 | GABPB2 | 1 | negative |
| ENSG00000143486 | EIF2D | 1 | negative |
| ENSG00000143493 | INTS7 | 1 | negative |
| ENSG00000143575 | HAX1 | 1 | negative |
| ENSG00000143578 | CREB3L4 | 1 | negative |
| ENSG00000143624 | INTS3 | 1 | negative |
| ENSG00000143753 | DEGS1 | 1 | negative |
| ENSG00000143776 | CDC42BPA | 1 | negative |
| ENSG00000143816 | WNT9A | 1 | negative |
| ENSG00000143842 | SOX13 | 1 | negative |
| ENSG00000143933 | CALM2 | 1 | negative |
| ENSG00000143942 | CHAC2 | 1 | negative |
| ENSG00000143951 | WDPCP | 1 | negative |
| ENSG00000143977 | SNRPG | 1 | negative |
| ENSG00000144028 | SNRNP200 | 1 | negative |
| ENSG00000144029 | MRPS5 | 1 | negative |
| ENSG00000144048 | DUSP11 | 1 | negative |
| ENSG00000144218 | AFF3 | 1 | negative |
| ENSG00000144231 | POLR2D | 1 | negative |
| ENSG00000144339 | TMEFF2 | 1 | negative |
| ENSG00000144381 | HSPD1 | 1 | negative |
| ENSG00000144452 | ABCA12 | 1 | negative |
| ENSG00000144455 | SUMF1 | 1 | negative |
| ENSG00000144524 | COPS7B | 1 | negative |
| ENSG00000144554 | FANCD2 | 1 | negative |
| ENSG00000144580 | CNOT9 | 1 | negative |
| ENSG00000144642 | RBMS3 | 1 | negative |
| ENSG00000144736 | SHQ1 | 1 | negative |
| ENSG00000144744 | UBA3 | 1 | negative |
| ENSG00000144824 | PHLDB2 | 1 | negative |
| ENSG00000145247 | OCIAD2 | 1 | negative |
| ENSG00000145284 | SCD5 | 1 | negative |
| ENSG00000145337 | PYURF | 1 | negative |
| ENSG00000145349 | CAMK2D | 1 | negative |
| ENSG00000145354 | CISD2 | 1 | negative |
| ENSG00000145375 | SPATA5 | 1 | negative |
| ENSG00000145384 | FABP2 | 1 | negative |
| ENSG00000145388 | METTL14 | 1 | negative |
| ENSG00000145391 | SETD7 | 1 | negative |
| ENSG00000145425 | RPS3A | 1 | negative |
| ENSG00000145494 | NDUFS6 | 1 | negative |
| ENSG00000145545 | SRD5A1 | 1 | negative |
| ENSG00000145555 | MYO10 | 1 | negative |
| ENSG00000145757 | SPATA9 | 1 | negative |
| ENSG00000145819 | ARHGAP26 | 1 | negative |
| ENSG00000145907 | G3BP1 | 1 | negative |
| ENSG00000146063 | TRIM41 | 1 | negative |
| ENSG00000146066 | HIGD2A | 1 | negative |
| ENSG00000146085 | MUT | 1 | negative |
| ENSG00000146221 | TCTE1 | 1 | negative |
| ENSG00000146247 | PHIP | 1 | negative |
| ENSG00000146426 | TIAM2 | 1 | negative |
| ENSG00000146574 | CCZ1B | 1 | negative |
| ENSG00000146729 | NIPSNAP2 | 1 | negative |
| ENSG00000147010 | SH3KBP1 | 1 | negative |
| ENSG00000147100 | SLC16A2 | 1 | negative |
| ENSG00000147117 | ZNF157 | 1 | negative |
| ENSG00000147123 | NDUFB11 | 1 | negative |
| ENSG00000147127 | RAB41 | 1 | negative |
| ENSG00000147133 | TAF1 | 1 | negative |
| ENSG00000147234 | FRMPD3 | 1 | negative |
| ENSG00000147316 | MCPH1 | 1 | negative |
| ENSG00000147364 | FBXO25 | 1 | negative |
| ENSG00000147481 | SNTG1 | 1 | negative |
| ENSG00000147536 | GINS4 | 1 | negative |
| ENSG00000147596 | PRDM14 | 1 | negative |
| ENSG00000147614 | ATP6V0D2 | 1 | negative |
| ENSG00000147677 | EIF3H | 1 | negative |
| ENSG00000147804 | SLC39A4 | 1 | negative |
| ENSG00000147872 | PLIN2 | 1 | negative |
| ENSG00000148219 | ASTN2 | 1 | negative |
| ENSG00000148225 | WDR31 | 1 | negative |
| ENSG00000148444 | COMMD3 | 1 | negative |
| ENSG00000148604 | RGR | 1 | negative |
| ENSG00000148671 | ADIRF | 1 | negative |
| ENSG00000149179 | C11orf49 | 1 | negative |
| ENSG00000149554 | CHEK1 | 1 | negative |
| ENSG00000149823 | VPS51 | 1 | negative |
| ENSG00000150093 | ITGB1 | 1 | negative |
| ENSG00000150527 | CTAGE5 | 1 | negative |
| ENSG00000150540 | HNMT | 1 | negative |
| ENSG00000150593 | PDCD4 | 1 | negative |
| ENSG00000150625 | GPM6A | 1 | negative |
| ENSG00000150779 | TIMM8B | 1 | negative |
| ENSG00000150787 | PTS | 1 | negative |
| ENSG00000150967 | ABCB9 | 1 | negative |
| ENSG00000151116 | UEVLD | 1 | negative |
| ENSG00000151131 | C12orf45 | 1 | negative |
| ENSG00000151338 | MIPOL1 | 1 | negative |
| ENSG00000151474 | FRMD4A | 1 | negative |
| ENSG00000151491 | EPS8 | 1 | negative |
| ENSG00000151532 | VTI1A | 1 | negative |
| ENSG00000151657 | KIN | 1 | negative |
| ENSG00000151729 | SLC25A4 | 1 | negative |
| ENSG00000151914 | DST | 1 | negative |
| ENSG00000152147 | GEMIN6 | 1 | negative |
| ENSG00000152234 | ATP5F1A | 1 | negative |
| ENSG00000152253 | SPC25 | 1 | negative |
| ENSG00000152291 | TGOLN2 | 1 | negative |
| ENSG00000152292 | SH2D6 | 1 | negative |
| ENSG00000152348 | ATG10 | 1 | negative |
| ENSG00000152454 | ZNF256 | 1 | negative |
| ENSG00000152457 | DCLRE1C | 1 | negative |
| ENSG00000152492 | CCDC50 | 1 | negative |
| ENSG00000152620 | NADK2 | 1 | negative |
| ENSG00000152700 | SAR1B | 1 | negative |
| ENSG00000152767 | FARP1 | 1 | negative |
| ENSG00000152795 | HNRNPDL | 1 | negative |
| ENSG00000152936 | LMNTD1 | 1 | negative |
| ENSG00000152939 | MARVELD2 | 1 | negative |
| ENSG00000153037 | SRP19 | 1 | negative |
| ENSG00000153130 | SCOC | 1 | negative |
| ENSG00000153132 | CLGN | 1 | negative |
| ENSG00000153147 | SMARCA5 | 1 | negative |
| ENSG00000153179 | RASSF3 | 1 | negative |
| ENSG00000153292 | ADGRF1 | 1 | negative |
| ENSG00000153310 | FAM49B | 1 | negative |
| ENSG00000153391 | INO80C | 1 | negative |
| ENSG00000153721 | CNKSR3 | 1 | negative |
| ENSG00000153774 | CFDP1 | 1 | negative |
| ENSG00000153944 | MSI2 | 1 | negative |
| ENSG00000153975 | ZUFSP | 1 | negative |
| ENSG00000154237 | LRRK1 | 1 | negative |
| ENSG00000154240 | CEP112 | 1 | negative |
| ENSG00000154380 | ENAH | 1 | negative |
| ENSG00000154493 | C10orf90 | 1 | negative |
| ENSG00000154556 | SORBS2 | 1 | negative |
| ENSG00000154582 | ELOC | 1 | negative |
| ENSG00000154620 | TMSB4Y | 1 | negative |
| ENSG00000154654 | NCAM2 | 1 | negative |
| ENSG00000154803 | FLCN | 1 | negative |
| ENSG00000154839 | SKA1 | 1 | negative |
| ENSG00000154920 | EME1 | 1 | negative |
| ENSG00000154945 | ANKRD40 | 1 | negative |
| ENSG00000155008 | APOOL | 1 | negative |
| ENSG00000155066 | PROM2 | 1 | negative |
| ENSG00000155085 | AK9 | 1 | negative |
| ENSG00000155368 | DBI | 1 | negative |
| ENSG00000155463 | OXA1L | 1 | negative |
| ENSG00000155621 | C9orf85 | 1 | negative |
| ENSG00000155666 | KDM8 | 1 | negative |
| ENSG00000155792 | DEPTOR | 1 | negative |
| ENSG00000156170 | NDUFAF6 | 1 | negative |
| ENSG00000156218 | ADAMTSL3 | 1 | negative |
| ENSG00000156313 | RPGR | 1 | negative |
| ENSG00000156374 | PCGF6 | 1 | negative |
| ENSG00000156398 | SFXN2 | 1 | negative |
| ENSG00000156531 | PHF6 | 1 | negative |
| ENSG00000156675 | RAB11FIP1 | 1 | negative |
| ENSG00000156711 | MAPK13 | 1 | negative |
| ENSG00000156787 | TBC1D31 | 1 | negative |
| ENSG00000156802 | ATAD2 | 1 | negative |
| ENSG00000156869 | FRRS1 | 1 | negative |
| ENSG00000157014 | TATDN2 | 1 | negative |
| ENSG00000157036 | EXOG | 1 | negative |
| ENSG00000157060 | SHCBP1L | 1 | negative |
| ENSG00000157106 | SMG1 | 1 | negative |
| ENSG00000157212 | PAXIP1 | 1 | negative |
| ENSG00000157259 | GATAD1 | 1 | negative |
| ENSG00000157388 | CACNA1D | 1 | negative |
| ENSG00000157404 | KIT | 1 | negative |
| ENSG00000157800 | SLC37A3 | 1 | negative |
| ENSG00000157895 | C12orf43 | 1 | negative |
| ENSG00000158014 | SLC30A2 | 1 | negative |
| ENSG00000158161 | EYA3 | 1 | negative |
| ENSG00000158195 | WASF2 | 1 | negative |
| ENSG00000158321 | AUTS2 | 1 | negative |
| ENSG00000158417 | EIF5B | 1 | negative |
| ENSG00000158470 | B4GALT5 | 1 | negative |
| ENSG00000158528 | PPP1R9A | 1 | negative |
| ENSG00000158545 | ZC3H18 | 1 | negative |
| ENSG00000158691 | ZSCAN12 | 1 | negative |
| ENSG00000158864 | NDUFS2 | 1 | negative |
| ENSG00000158987 | RAPGEF6 | 1 | negative |
| ENSG00000159251 | ACTC1 | 1 | negative |
| ENSG00000159377 | PSMB4 | 1 | negative |
| ENSG00000159433 | STARD9 | 1 | negative |
| ENSG00000159579 | RSPRY1 | 1 | negative |
| ENSG00000159763 | PIP | 1 | negative |
| ENSG00000159788 | RGS12 | 1 | negative |
| ENSG00000159882 | ZNF230 | 1 | negative |
| ENSG00000160049 | DFFA | 1 | negative |
| ENSG00000160051 | IQCC | 1 | negative |
| ENSG00000160199 | PKNOX1 | 1 | negative |
| ENSG00000160214 | RRP1 | 1 | negative |
| ENSG00000160439 | RDH13 | 1 | negative |
| ENSG00000161010 | MRNIP | 1 | negative |
| ENSG00000161016 | RPL8 | 1 | negative |
| ENSG00000161057 | PSMC2 | 1 | negative |
| ENSG00000161243 | FBXO27 | 1 | negative |
| ENSG00000161267 | BDH1 | 1 | negative |
| ENSG00000161634 | DCD | 1 | negative |
| ENSG00000161640 | SIGLEC11 | 1 | negative |
| ENSG00000161847 | RAVER1 | 1 | negative |
| ENSG00000161929 | SCIMP | 1 | negative |
| ENSG00000161980 | POLR3K | 1 | negative |
| ENSG00000161996 | WDR90 | 1 | negative |
| ENSG00000162032 | SPSB3 | 1 | negative |
| ENSG00000162076 | FLYWCH2 | 1 | negative |
| ENSG00000162129 | CLPB | 1 | negative |
| ENSG00000162144 | CYB561A3 | 1 | negative |
| ENSG00000162377 | COA7 | 1 | negative |
| ENSG00000162408 | NOL9 | 1 | negative |
| ENSG00000162419 | GMEB1 | 1 | negative |
| ENSG00000162545 | CAMK2N1 | 1 | negative |
| ENSG00000162576 | MXRA8 | 1 | negative |
| ENSG00000162604 | TM2D1 | 1 | negative |
| ENSG00000162734 | PEA15 | 1 | negative |
| ENSG00000162757 | C1orf74 | 1 | negative |
| ENSG00000162769 | FLVCR1 | 1 | negative |
| ENSG00000162772 | ATF3 | 1 | negative |
| ENSG00000162813 | BPNT1 | 1 | negative |
| ENSG00000162819 | BROX | 1 | negative |
| ENSG00000162910 | MRPL55 | 1 | negative |
| ENSG00000162928 | PEX13 | 1 | negative |
| ENSG00000162929 | KIAA1841 | 1 | negative |
| ENSG00000163032 | VSNL1 | 1 | negative |
| ENSG00000163041 | H3F3A | 1 | negative |
| ENSG00000163075 | CFAP221 | 1 | negative |
| ENSG00000163170 | BOLA3 | 1 | negative |
| ENSG00000163322 | ABRAXAS1 | 1 | negative |
| ENSG00000163359 | COL6A3 | 1 | negative |
| ENSG00000163428 | LRRC58 | 1 | negative |
| ENSG00000163535 | SGO2 | 1 | negative |
| ENSG00000163536 | SERPINI1 | 1 | negative |
| ENSG00000163626 | COX18 | 1 | negative |
| ENSG00000163637 | PRICKLE2 | 1 | negative |
| ENSG00000163682 | RPL9 | 1 | negative |
| ENSG00000163689 | C3orf67 | 1 | negative |
| ENSG00000163694 | RBM47 | 1 | negative |
| ENSG00000163781 | TOPBP1 | 1 | negative |
| ENSG00000163794 | UCN | 1 | negative |
| ENSG00000163795 | ZNF513 | 1 | negative |
| ENSG00000163798 | SLC4A1AP | 1 | negative |
| ENSG00000163867 | ZMYM6 | 1 | negative |
| ENSG00000163879 | DNALI1 | 1 | negative |
| ENSG00000163959 | SLC51A | 1 | negative |
| ENSG00000163961 | RNF168 | 1 | negative |
| ENSG00000163964 | PIGX | 1 | negative |
| ENSG00000164039 | BDH2 | 1 | negative |
| ENSG00000164040 | PGRMC2 | 1 | negative |
| ENSG00000164048 | ZNF589 | 1 | negative |
| ENSG00000164066 | INTU | 1 | negative |
| ENSG00000164080 | RAD54L2 | 1 | negative |
| ENSG00000164082 | GRM2 | 1 | negative |
| ENSG00000164096 | C4orf3 | 1 | negative |
| ENSG00000164106 | SCRG1 | 1 | negative |
| ENSG00000164109 | MAD2L1 | 1 | negative |
| ENSG00000164111 | ANXA5 | 1 | negative |
| ENSG00000164134 | NAA15 | 1 | negative |
| ENSG00000164161 | HHIP | 1 | negative |
| ENSG00000164164 | OTUD4 | 1 | negative |
| ENSG00000164176 | EDIL3 | 1 | negative |
| ENSG00000164199 | ADGRV1 | 1 | negative |
| ENSG00000164237 | CMBL | 1 | negative |
| ENSG00000164403 | SHROOM1 | 1 | negative |
| ENSG00000164466 | SFXN1 | 1 | negative |
| ENSG00000164548 | TRA2A | 1 | negative |
| ENSG00000164627 | KIF6 | 1 | negative |
| ENSG00000164695 | CHMP4C | 1 | negative |
| ENSG00000164756 | SLC30A8 | 1 | negative |
| ENSG00000164796 | CSMD3 | 1 | negative |
| ENSG00000164902 | PHAX | 1 | negative |
| ENSG00000164904 | ALDH7A1 | 1 | negative |
| ENSG00000164920 | OSR2 | 1 | negative |
| ENSG00000165055 | METTL2B | 1 | negative |
| ENSG00000165084 | C8orf34 | 1 | negative |
| ENSG00000165119 | HNRNPK | 1 | negative |
| ENSG00000165140 | FBP1 | 1 | negative |
| ENSG00000165219 | GAPVD1 | 1 | negative |
| ENSG00000165264 | NDUFB6 | 1 | negative |
| ENSG00000165272 | AQP3 | 1 | negative |
| ENSG00000165280 | VCP | 1 | negative |
| ENSG00000165416 | SUGT1 | 1 | negative |
| ENSG00000165527 | ARF6 | 1 | negative |
| ENSG00000165629 | ATP5F1C | 1 | negative |
| ENSG00000165672 | PRDX3 | 1 | negative |
| ENSG00000165678 | GHITM | 1 | negative |
| ENSG00000165688 | PMPCA | 1 | negative |
| ENSG00000165795 | NDRG2 | 1 | negative |
| ENSG00000165810 | BTNL9 | 1 | negative |
| ENSG00000165868 | HSPA12A | 1 | negative |
| ENSG00000165916 | PSMC3 | 1 | negative |
| ENSG00000166033 | HTRA1 | 1 | negative |
| ENSG00000166106 | ADAMTS15 | 1 | negative |
| ENSG00000166136 | NDUFB8 | 1 | negative |
| ENSG00000166165 | CKB | 1 | negative |
| ENSG00000166377 | ATP9B | 1 | negative |
| ENSG00000166411 | IDH3A | 1 | negative |
| ENSG00000166477 | LEO1 | 1 | negative |
| ENSG00000166575 | TMEM135 | 1 | negative |
| ENSG00000166578 | IQCD | 1 | negative |
| ENSG00000166595 | FAM96B | 1 | negative |
| ENSG00000166681 | BEX3 | 1 | negative |
| ENSG00000166796 | LDHC | 1 | negative |
| ENSG00000166816 | LDHD | 1 | negative |
| ENSG00000166913 | YWHAB | 1 | negative |
| ENSG00000166938 | DIS3L | 1 | negative |
| ENSG00000166965 | RCCD1 | 1 | negative |
| ENSG00000167123 | CERCAM | 1 | negative |
| ENSG00000167191 | GPRC5B | 1 | negative |
| ENSG00000167196 | FBXO22 | 1 | negative |
| ENSG00000167207 | NOD2 | 1 | negative |
| ENSG00000167232 | ZNF91 | 1 | negative |
| ENSG00000167315 | ACAA2 | 1 | negative |
| ENSG00000167536 | DHRS13 | 1 | negative |
| ENSG00000167549 | CORO6 | 1 | negative |
| ENSG00000167552 | TUBA1A | 1 | negative |
| ENSG00000167637 | ZNF283 | 1 | negative |
| ENSG00000167642 | SPINT2 | 1 | negative |
| ENSG00000167658 | EEF2 | 1 | negative |
| ENSG00000167685 | ZNF444 | 1 | negative |
| ENSG00000167699 | GLOD4 | 1 | negative |
| ENSG00000167733 | HSD11B1L | 1 | negative |
| ENSG00000167741 | GGT6 | 1 | negative |
| ENSG00000167757 | KLK11 | 1 | negative |
| ENSG00000167769 | ACER1 | 1 | negative |
| ENSG00000167815 | PRDX2 | 1 | negative |
| ENSG00000167904 | TMEM68 | 1 | negative |
| ENSG00000167986 | DDB1 | 1 | negative |
| ENSG00000167992 | VWCE | 1 | negative |
| ENSG00000168028 | RPSA | 1 | negative |
| ENSG00000168036 | CTNNB1 | 1 | negative |
| ENSG00000168090 | COPS6 | 1 | negative |
| ENSG00000168216 | LMBRD1 | 1 | negative |
| ENSG00000168228 | ZCCHC4 | 1 | negative |
| ENSG00000168264 | IRF2BP2 | 1 | negative |
| ENSG00000168385 | 2-Sep | 1 | negative |
| ENSG00000168393 | DTYMK | 1 | negative |
| ENSG00000168538 | TRAPPC11 | 1 | negative |
| ENSG00000168612 | ZSWIM1 | 1 | negative |
| ENSG00000168653 | NDUFS5 | 1 | negative |
| ENSG00000168672 | FAM84B | 1 | negative |
| ENSG00000168676 | KCTD19 | 1 | negative |
| ENSG00000168806 | LCMT2 | 1 | negative |
| ENSG00000168807 | SNTB2 | 1 | negative |
| ENSG00000168827 | GFM1 | 1 | negative |
| ENSG00000168872 | DDX19A | 1 | negative |
| ENSG00000168899 | VAMP5 | 1 | negative |
| ENSG00000168906 | MAT2A | 1 | negative |
| ENSG00000168924 | LETM1 | 1 | negative |
| ENSG00000169020 | ATP5ME | 1 | negative |
| ENSG00000169021 | UQCRFS1 | 1 | negative |
| ENSG00000169045 | HNRNPH1 | 1 | negative |
| ENSG00000169084 | DHRSX | 1 | negative |
| ENSG00000169189 | NSMCE1 | 1 | negative |
| ENSG00000169223 | LMAN2 | 1 | negative |
| ENSG00000169228 | RAB24 | 1 | negative |
| ENSG00000169347 | GP2 | 1 | negative |
| ENSG00000169359 | SLC33A1 | 1 | negative |
| ENSG00000169504 | CLIC4 | 1 | negative |
| ENSG00000169564 | PCBP1 | 1 | negative |
| ENSG00000169583 | CLIC3 | 1 | negative |
| ENSG00000169609 | C15orf40 | 1 | negative |
| ENSG00000169689 | CENPX | 1 | negative |
| ENSG00000169714 | CNBP | 1 | negative |
| ENSG00000169738 | DCXR | 1 | negative |
| ENSG00000169762 | TAPT1 | 1 | negative |
| ENSG00000169895 | SYAP1 | 1 | negative |
| ENSG00000169905 | TOR1AIP2 | 1 | negative |
| ENSG00000169914 | OTUD3 | 1 | negative |
| ENSG00000170044 | ZPLD1 | 1 | negative |
| ENSG00000170088 | TMEM192 | 1 | negative |
| ENSG00000170270 | GON7 | 1 | negative |
| ENSG00000170310 | STX8 | 1 | negative |
| ENSG00000170345 | FOS | 1 | negative |
| ENSG00000170430 | MGMT | 1 | negative |
| ENSG00000170445 | HARS | 1 | negative |
| ENSG00000170464 | DNAJC18 | 1 | negative |
| ENSG00000170606 | HSPA4 | 1 | negative |
| ENSG00000170631 | ZNF16 | 1 | negative |
| ENSG00000170683 | OR10A3 | 1 | negative |
| ENSG00000170689 | HOXB9 | 1 | negative |
| ENSG00000170734 | POLH | 1 | negative |
| ENSG00000170906 | NDUFA3 | 1 | negative |
| ENSG00000171222 | SCAND1 | 1 | negative |
| ENSG00000171303 | KCNK3 | 1 | negative |
| ENSG00000171345 | KRT19 | 1 | negative |
| ENSG00000171453 | POLR1C | 1 | negative |
| ENSG00000171469 | ZNF561 | 1 | negative |
| ENSG00000171490 | RSL1D1 | 1 | negative |
| ENSG00000171503 | ETFDH | 1 | negative |
| ENSG00000171561 | OR2AT4 | 1 | negative |
| ENSG00000171714 | ANO5 | 1 | negative |
| ENSG00000171723 | GPHN | 1 | negative |
| ENSG00000171813 | PWWP2B | 1 | negative |
| ENSG00000171824 | EXOSC10 | 1 | negative |
| ENSG00000171931 | FBXW10 | 1 | negative |
| ENSG00000171962 | DRC3 | 1 | negative |
| ENSG00000172037 | LAMB2 | 1 | negative |
| ENSG00000172113 | NME6 | 1 | negative |
| ENSG00000172146 | OR1A1 | 1 | negative |
| ENSG00000172167 | MTBP | 1 | negative |
| ENSG00000172171 | TEFM | 1 | negative |
| ENSG00000172197 | MBOAT1 | 1 | negative |
| ENSG00000172239 | PAIP1 | 1 | negative |
| ENSG00000172260 | NEGR1 | 1 | negative |
| ENSG00000172264 | MACROD2 | 1 | negative |
| ENSG00000172273 | HINFP | 1 | negative |
| ENSG00000172339 | ALG14 | 1 | negative |
| ENSG00000172380 | GNG12 | 1 | negative |
| ENSG00000172476 | RAB40A | 1 | negative |
| ENSG00000172663 | TMEM134 | 1 | negative |
| ENSG00000172689 | MS4A10 | 1 | negative |
| ENSG00000172742 | OR4D9 | 1 | negative |
| ENSG00000172771 | EFCAB12 | 1 | negative |
| ENSG00000172888 | ZNF621 | 1 | negative |
| ENSG00000172954 | LCLAT1 | 1 | negative |
| ENSG00000172992 | DCAKD | 1 | negative |
| ENSG00000173163 | COMMD1 | 1 | negative |
| ENSG00000173198 | CYSLTR1 | 1 | negative |
| ENSG00000173227 | SYT12 | 1 | negative |
| ENSG00000173230 | GOLGB1 | 1 | negative |
| ENSG00000173267 | SNCG | 1 | negative |
| ENSG00000173269 | MMRN2 | 1 | negative |
| ENSG00000173273 | TNKS | 1 | negative |
| ENSG00000173275 | ZNF449 | 1 | negative |
| ENSG00000173402 | DAG1 | 1 | negative |
| ENSG00000173480 | ZNF417 | 1 | negative |
| ENSG00000173531 | MST1 | 1 | negative |
| ENSG00000173575 | CHD2 | 1 | negative |
| ENSG00000173611 | SCAI | 1 | negative |
| ENSG00000173681 | BCLAF3 | 1 | negative |
| ENSG00000173726 | TOMM20 | 1 | negative |
| ENSG00000173801 | JUP | 1 | negative |
| ENSG00000173821 | RNF213 | 1 | negative |
| ENSG00000173826 | KCNH6 | 1 | negative |
| ENSG00000173848 | NET1 | 1 | negative |
| ENSG00000173875 | ZNF791 | 1 | negative |
| ENSG00000173889 | PHC3 | 1 | negative |
| ENSG00000173890 | GPR160 | 1 | negative |
| ENSG00000173915 | ATP5MD | 1 | negative |
| ENSG00000174021 | GNG5 | 1 | negative |
| ENSG00000174109 | C16orf91 | 1 | negative |
| ENSG00000174165 | ZDHHC24 | 1 | negative |
| ENSG00000174231 | PRPF8 | 1 | negative |
| ENSG00000174428 | GTF2IRD2B | 1 | negative |
| ENSG00000174483 | BBS1 | 1 | negative |
| ENSG00000174796 | THAP6 | 1 | negative |
| ENSG00000174891 | RSRC1 | 1 | negative |
| ENSG00000174953 | DHX36 | 1 | negative |
| ENSG00000174992 | ZG16 | 1 | negative |
| ENSG00000175066 | GK5 | 1 | negative |
| ENSG00000175130 | MARCKSL1 | 1 | negative |
| ENSG00000175198 | PCCA | 1 | negative |
| ENSG00000175334 | BANF1 | 1 | negative |
| ENSG00000175575 | PAAF1 | 1 | negative |
| ENSG00000175581 | MRPL48 | 1 | negative |
| ENSG00000175595 | ERCC4 | 1 | negative |
| ENSG00000175600 | SUGCT | 1 | negative |
| ENSG00000175768 | TOMM5 | 1 | negative |
| ENSG00000175782 | SLC35E3 | 1 | negative |
| ENSG00000175787 | ZNF169 | 1 | negative |
| ENSG00000175874 | CREG2 | 1 | negative |
| ENSG00000176040 | TMPRSS7 | 1 | negative |
| ENSG00000176046 | NUPR1 | 1 | negative |
| ENSG00000176087 | SLC35A4 | 1 | negative |
| ENSG00000176101 | SSNA1 | 1 | negative |
| ENSG00000176155 | CCDC57 | 1 | negative |
| ENSG00000176225 | RTTN | 1 | negative |
| ENSG00000176244 | ACBD7 | 1 | negative |
| ENSG00000176261 | ZBTB8OS | 1 | negative |
| ENSG00000176406 | RIMS2 | 1 | negative |
| ENSG00000176422 | SPRYD4 | 1 | negative |
| ENSG00000176485 | PLA2G16 | 1 | negative |
| ENSG00000176623 | RMDN1 | 1 | negative |
| ENSG00000176695 | OR4F17 | 1 | negative |
| ENSG00000176845 | METRNL | 1 | negative |
| ENSG00000176871 | WSB2 | 1 | negative |
| ENSG00000176887 | SOX11 | 1 | negative |
| ENSG00000177082 | WDR73 | 1 | negative |
| ENSG00000177084 | POLE | 1 | negative |
| ENSG00000177225 | GATD1 | 1 | negative |
| ENSG00000177238 | TRIM72 | 1 | negative |
| ENSG00000177302 | TOP3A | 1 | negative |
| ENSG00000177352 | CCDC71 | 1 | negative |
| ENSG00000177599 | ZNF491 | 1 | negative |
| ENSG00000177606 | JUN | 1 | negative |
| ENSG00000177853 | ZNF518A | 1 | negative |
| ENSG00000177868 | SVBP | 1 | negative |
| ENSG00000177889 | UBE2N | 1 | negative |
| ENSG00000178053 | MLF1 | 1 | negative |
| ENSG00000178075 | GRAMD1C | 1 | negative |
| ENSG00000178149 | DALRD3 | 1 | negative |
| ENSG00000178171 | AMER3 | 1 | negative |
| ENSG00000178229 | ZNF543 | 1 | negative |
| ENSG00000178537 | SLC25A20 | 1 | negative |
| ENSG00000178665 | ZNF713 | 1 | negative |
| ENSG00000178741 | COX5A | 1 | negative |
| ENSG00000179163 | FUCA1 | 1 | negative |
| ENSG00000179168 | GGN | 1 | negative |
| ENSG00000179222 | MAGED1 | 1 | negative |
| ENSG00000179295 | PTPN11 | 1 | negative |
| ENSG00000179820 | MYADM | 1 | negative |
| ENSG00000179909 | ZNF154 | 1 | negative |
| ENSG00000179912 | R3HDM2 | 1 | negative |
| ENSG00000179918 | SEPHS2 | 1 | negative |
| ENSG00000180251 | SLC9A4 | 1 | negative |
| ENSG00000180346 | TIGD2 | 1 | negative |
| ENSG00000180357 | ZNF609 | 1 | negative |
| ENSG00000180638 | SLC47A2 | 1 | negative |
| ENSG00000180815 | MAP3K15 | 1 | negative |
| ENSG00000180855 | ZNF443 | 1 | negative |
| ENSG00000180881 | CAPS2 | 1 | negative |
| ENSG00000180974 | OR52E4 | 1 | negative |
| ENSG00000180998 | GPR137C | 1 | negative |
| ENSG00000181045 | SLC26A11 | 1 | negative |
| ENSG00000181090 | EHMT1 | 1 | negative |
| ENSG00000181274 | FRAT2 | 1 | negative |
| ENSG00000181392 | SYNE4 | 1 | negative |
| ENSG00000181396 | OGFOD3 | 1 | negative |
| ENSG00000181458 | TMEM45A | 1 | negative |
| ENSG00000181481 | RNF135 | 1 | negative |
| ENSG00000181513 | ACBD4 | 1 | negative |
| ENSG00000181610 | MRPS23 | 1 | negative |
| ENSG00000181626 | ANKRD62 | 1 | negative |
| ENSG00000181666 | HKR1 | 1 | negative |
| ENSG00000181704 | YIPF6 | 1 | negative |
| ENSG00000181785 | OR5AS1 | 1 | negative |
| ENSG00000181873 | IBA57 | 1 | negative |
| ENSG00000181991 | MRPS11 | 1 | negative |
| ENSG00000182173 | TSEN54 | 1 | negative |
| ENSG00000182405 | PGBD4 | 1 | negative |
| ENSG00000182446 | NPLOC4 | 1 | negative |
| ENSG00000182504 | CEP97 | 1 | negative |
| ENSG00000182612 | TSPAN10 | 1 | negative |
| ENSG00000182621 | PLCB1 | 1 | negative |
| ENSG00000182718 | ANXA2 | 1 | negative |
| ENSG00000182749 | PAQR7 | 1 | negative |
| ENSG00000182768 | NGRN | 1 | negative |
| ENSG00000182963 | GJC1 | 1 | negative |
| ENSG00000183145 | RIPPLY3 | 1 | negative |
| ENSG00000183160 | TMEM119 | 1 | negative |
| ENSG00000183206 | POTEC | 1 | negative |
| ENSG00000183207 | RUVBL2 | 1 | negative |
| ENSG00000183323 | CCDC125 | 1 | negative |
| ENSG00000183401 | CCDC159 | 1 | negative |
| ENSG00000183741 | CBX6 | 1 | negative |
| ENSG00000183742 | MACC1 | 1 | negative |
| ENSG00000183844 | FAM3B | 1 | negative |
| ENSG00000184058 | TBX1 | 1 | negative |
| ENSG00000184220 | CMSS1 | 1 | negative |
| ENSG00000184349 | EFNA5 | 1 | negative |
| ENSG00000184432 | COPB2 | 1 | negative |
| ENSG00000184445 | KNTC1 | 1 | negative |
| ENSG00000184574 | LPAR5 | 1 | negative |
| ENSG00000184619 | KRBA2 | 1 | negative |
| ENSG00000184635 | ZNF93 | 1 | negative |
| ENSG00000184698 | OR51M1 | 1 | negative |
| ENSG00000184743 | ATL3 | 1 | negative |
| ENSG00000184752 | NDUFA12 | 1 | negative |
| ENSG00000184788 | SATL1 | 1 | negative |
| ENSG00000184857 | TMEM186 | 1 | negative |
| ENSG00000184922 | FMNL1 | 1 | negative |
| ENSG00000184992 | BRI3BP | 1 | negative |
| ENSG00000185046 | ANKS1B | 1 | negative |
| ENSG00000185122 | HSF1 | 1 | negative |
| ENSG00000185219 | ZNF445 | 1 | negative |
| ENSG00000185344 | ATP6V0A2 | 1 | negative |
| ENSG00000185379 | RAD51D | 1 | negative |
| ENSG00000185567 | AHNAK2 | 1 | negative |
| ENSG00000185585 | OLFML2A | 1 | negative |
| ENSG00000185869 | ZNF829 | 1 | negative |
| ENSG00000185960 | SHOX | 1 | negative |
| ENSG00000186001 | LRCH3 | 1 | negative |
| ENSG00000186094 | AGBL4 | 1 | negative |
| ENSG00000186193 | SAPCD2 | 1 | negative |
| ENSG00000186260 | MKL2 | 1 | negative |
| ENSG00000186283 | TOR3A | 1 | negative |
| ENSG00000186298 | PPP1CC | 1 | negative |
| ENSG00000186329 | TMEM212 | 1 | negative |
| ENSG00000186350 | RXRA | 1 | negative |
| ENSG00000186487 | MYT1L | 1 | negative |
| ENSG00000186871 | ERCC6L | 1 | negative |
| ENSG00000186891 | TNFRSF18 | 1 | negative |
| ENSG00000186973 | FAM183A | 1 | negative |
| ENSG00000187147 | RNF220 | 1 | negative |
| ENSG00000187186 | AL162231.1 | 1 | negative |
| ENSG00000187240 | DYNC2H1 | 1 | negative |
| ENSG00000187244 | BCAM | 1 | negative |
| ENSG00000187514 | PTMA | 1 | negative |
| ENSG00000187555 | USP7 | 1 | negative |
| ENSG00000187642 | PERM1 | 1 | negative |
| ENSG00000187715 | KBTBD12 | 1 | negative |
| ENSG00000187905 | LRRC74B | 1 | negative |
| ENSG00000187997 | C17orf99 | 1 | negative |
| ENSG00000188042 | ARL4C | 1 | negative |
| ENSG00000188295 | ZNF669 | 1 | negative |
| ENSG00000188305 | PEAK3 | 1 | negative |
| ENSG00000188322 | SBK1 | 1 | negative |
| ENSG00000188338 | SLC38A3 | 1 | negative |
| ENSG00000188596 | CFAP54 | 1 | negative |
| ENSG00000188603 | CLN3 | 1 | negative |
| ENSG00000188613 | NANOS1 | 1 | negative |
| ENSG00000188643 | S100A16 | 1 | negative |
| ENSG00000188738 | FSIP2 | 1 | negative |
| ENSG00000188976 | NOC2L | 1 | negative |
| ENSG00000188981 | MSANTD1 | 1 | negative |
| ENSG00000189060 | H1F0 | 1 | negative |
| ENSG00000189091 | SF3B3 | 1 | negative |
| ENSG00000189114 | BLOC1S3 | 1 | negative |
| ENSG00000189157 | FAM47E | 1 | negative |
| ENSG00000189159 | JPT1 | 1 | negative |
| ENSG00000189241 | TSPYL1 | 1 | negative |
| ENSG00000189266 | PNRC2 | 1 | negative |
| ENSG00000189339 | SLC35E2B | 1 | negative |
| ENSG00000196074 | SYCP2 | 1 | negative |
| ENSG00000196141 | SPATS2L | 1 | negative |
| ENSG00000196150 | ZNF250 | 1 | negative |
| ENSG00000196152 | ZNF79 | 1 | negative |
| ENSG00000196214 | ZNF766 | 1 | negative |
| ENSG00000196220 | SRGAP3 | 1 | negative |
| ENSG00000196230 | TUBB | 1 | negative |
| ENSG00000196275 | GTF2IRD2 | 1 | negative |
| ENSG00000196305 | IARS | 1 | negative |
| ENSG00000196312 | MFSD14C | 1 | negative |
| ENSG00000196337 | CGB7 | 1 | negative |
| ENSG00000196378 | ZNF34 | 1 | negative |
| ENSG00000196417 | ZNF765 | 1 | negative |
| ENSG00000196418 | ZNF124 | 1 | negative |
| ENSG00000196419 | XRCC6 | 1 | negative |
| ENSG00000196458 | ZNF605 | 1 | negative |
| ENSG00000196466 | ZNF799 | 1 | negative |
| ENSG00000196498 | NCOR2 | 1 | negative |
| ENSG00000196505 | GDAP2 | 1 | negative |
| ENSG00000196586 | MYO6 | 1 | negative |
| ENSG00000196591 | HDAC2 | 1 | negative |
| ENSG00000196739 | COL27A1 | 1 | negative |
| ENSG00000196754 | S100A2 | 1 | negative |
| ENSG00000196793 | ZNF239 | 1 | negative |
| ENSG00000196917 | HCAR1 | 1 | negative |
| ENSG00000196935 | SRGAP1 | 1 | negative |
| ENSG00000197044 | ZNF441 | 1 | negative |
| ENSG00000197056 | ZMYM1 | 1 | negative |
| ENSG00000197061 | HIST1H4C | 1 | negative |
| ENSG00000197070 | ARRDC1 | 1 | negative |
| ENSG00000197102 | DYNC1H1 | 1 | negative |
| ENSG00000197111 | PCBP2 | 1 | negative |
| ENSG00000197140 | ADAM32 | 1 | negative |
| ENSG00000197153 | HIST1H3J | 1 | negative |
| ENSG00000197168 | NEK5 | 1 | negative |
| ENSG00000197256 | KANK2 | 1 | negative |
| ENSG00000197279 | ZNF165 | 1 | negative |
| ENSG00000197372 | ZNF675 | 1 | negative |
| ENSG00000197380 | DACT3 | 1 | negative |
| ENSG00000197405 | C5AR1 | 1 | negative |
| ENSG00000197483 | ZNF628 | 1 | negative |
| ENSG00000197497 | ZNF665 | 1 | negative |
| ENSG00000197532 | OR6Y1 | 1 | negative |
| ENSG00000197558 | SSPO | 1 | negative |
| ENSG00000197563 | PIGN | 1 | negative |
| ENSG00000197587 | DMBX1 | 1 | negative |
| ENSG00000197594 | ENPP1 | 1 | negative |
| ENSG00000197694 | SPTAN1 | 1 | negative |
| ENSG00000197712 | FAM114A1 | 1 | negative |
| ENSG00000197721 | CR1L | 1 | negative |
| ENSG00000197746 | PSAP | 1 | negative |
| ENSG00000197747 | S100A10 | 1 | negative |
| ENSG00000197756 | RPL37A | 1 | negative |
| ENSG00000197912 | SPG7 | 1 | negative |
| ENSG00000197937 | ZNF347 | 1 | negative |
| ENSG00000197958 | RPL12 | 1 | negative |
| ENSG00000197961 | ZNF121 | 1 | negative |
| ENSG00000198000 | NOL8 | 1 | negative |
| ENSG00000198131 | ZNF544 | 1 | negative |
| ENSG00000198189 | HSD17B11 | 1 | negative |
| ENSG00000198203 | SULT1C2 | 1 | negative |
| ENSG00000198270 | TMEM116 | 1 | negative |
| ENSG00000198420 | TCAF1 | 1 | negative |
| ENSG00000198429 | ZNF69 | 1 | negative |
| ENSG00000198492 | YTHDF2 | 1 | negative |
| ENSG00000198502 | HLA-DRB5 | 1 | negative |
| ENSG00000198538 | ZNF28 | 1 | negative |
| ENSG00000198563 | DDX39B | 1 | negative |
| ENSG00000198625 | MDM4 | 1 | negative |
| ENSG00000198692 | EIF1AY | 1 | negative |
| ENSG00000198700 | IPO9 | 1 | negative |
| ENSG00000198720 | ANKRD13B | 1 | negative |
| ENSG00000198729 | PPP1R14C | 1 | negative |
| ENSG00000198755 | RPL10A | 1 | negative |
| ENSG00000198791 | CNOT7 | 1 | negative |
| ENSG00000198815 | FOXJ3 | 1 | negative |
| ENSG00000198830 | HMGN2 | 1 | negative |
| ENSG00000198846 | TOX | 1 | negative |
| ENSG00000198860 | TSEN15 | 1 | negative |
| ENSG00000198865 | CCDC152 | 1 | negative |
| ENSG00000198919 | DZIP3 | 1 | negative |
| ENSG00000203705 | TATDN3 | 1 | negative |
| ENSG00000203772 | SPRN | 1 | negative |
| ENSG00000203805 | PLPP4 | 1 | negative |
| ENSG00000203880 | PCMTD2 | 1 | negative |
| ENSG00000203943 | SAMD13 | 1 | negative |
| ENSG00000204104 | TRAF3IP1 | 1 | negative |
| ENSG00000204120 | GIGYF2 | 1 | negative |
| ENSG00000204138 | PHACTR4 | 1 | negative |
| ENSG00000204152 | TIMM23B | 1 | negative |
| ENSG00000204176 | SYT15 | 1 | negative |
| ENSG00000204179 | PTPN20 | 1 | negative |
| ENSG00000204271 | SPIN3 | 1 | negative |
| ENSG00000204272 | NBDY | 1 | negative |
| ENSG00000204370 | SDHD | 1 | negative |
| ENSG00000204371 | EHMT2 | 1 | negative |
| ENSG00000204387 | C6orf48 | 1 | negative |
| ENSG00000204410 | MSH5 | 1 | negative |
| ENSG00000204514 | ZNF814 | 1 | negative |
| ENSG00000204576 | PRR3 | 1 | negative |
| ENSG00000204822 | MRPL53 | 1 | negative |
| ENSG00000204852 | TCTN1 | 1 | negative |
| ENSG00000204983 | PRSS1 | 1 | negative |
| ENSG00000204991 | SPIRE2 | 1 | negative |
| ENSG00000205133 | TRIQK | 1 | negative |
| ENSG00000205659 | LIN52 | 1 | negative |
| ENSG00000205730 | ITPRIPL2 | 1 | negative |
| ENSG00000205758 | CRYZL1 | 1 | negative |
| ENSG00000205937 | RNPS1 | 1 | negative |
| ENSG00000206053 | JPT2 | 1 | negative |
| ENSG00000206418 | RAB12 | 1 | negative |
| ENSG00000206559 | ZCWPW2 | 1 | negative |
| ENSG00000206562 | METTL6 | 1 | negative |
| ENSG00000211445 | GPX3 | 1 | negative |
| ENSG00000211584 | SLC48A1 | 1 | negative |
| ENSG00000212916 | MAP10 | 1 | negative |
| ENSG00000213020 | ZNF611 | 1 | negative |
| ENSG00000213064 | SFT2D2 | 1 | negative |
| ENSG00000213066 | FGFR1OP | 1 | negative |
| ENSG00000213079 | SCAF8 | 1 | negative |
| ENSG00000213145 | CRIP1 | 1 | negative |
| ENSG00000213339 | QTRT1 | 1 | negative |
| ENSG00000213420 | GPC2 | 1 | negative |
| ENSG00000213463 | SYNJ2BP | 1 | negative |
| ENSG00000213523 | SRA1 | 1 | negative |
| ENSG00000213585 | VDAC1 | 1 | negative |
| ENSG00000213626 | LBH | 1 | negative |
| ENSG00000213722 | DDAH2 | 1 | negative |
| ENSG00000213853 | EMP2 | 1 | negative |
| ENSG00000213967 | ZNF726 | 1 | negative |
| ENSG00000214046 | SMIM7 | 1 | negative |
| ENSG00000214078 | CPNE1 | 1 | negative |
| ENSG00000214087 | ARL16 | 1 | negative |
| ENSG00000214113 | LYRM4 | 1 | negative |
| ENSG00000214336 | FOXI3 | 1 | negative |
| ENSG00000214367 | HAUS3 | 1 | negative |
| ENSG00000214456 | PLIN5 | 1 | negative |
| ENSG00000214954 | LRRC69 | 1 | negative |
| ENSG00000215021 | PHB2 | 1 | negative |
| ENSG00000215845 | TSTD1 | 1 | negative |
| ENSG00000218739 | CEBPZOS | 1 | negative |
| ENSG00000219435 | CATSPERZ | 1 | negative |
| ENSG00000221838 | AP4M1 | 1 | negative |
| ENSG00000221882 | OR3A2 | 1 | negative |
| ENSG00000221923 | ZNF880 | 1 | negative |
| ENSG00000221926 | TRIM16 | 1 | negative |
| ENSG00000221955 | SLC12A8 | 1 | negative |
| ENSG00000223496 | EXOSC6 | 1 | negative |
| ENSG00000223547 | ZNF844 | 1 | negative |
| ENSG00000224420 | ADM5 | 1 | negative |
| ENSG00000227500 | SCAMP4 | 1 | negative |
| ENSG00000228198 | OR2M3 | 1 | negative |
| ENSG00000228716 | DHFR | 1 | negative |
| ENSG00000228727 | SAPCD1 | 1 | negative |
| ENSG00000229833 | PET100 | 1 | negative |
| ENSG00000232112 | TMA7 | 1 | negative |
| ENSG00000232838 | PET117 | 1 | negative |
| ENSG00000233927 | RPS28 | 1 | negative |
| ENSG00000234444 | ZNF736 | 1 | negative |
| ENSG00000234545 | FAM133B | 1 | negative |
| ENSG00000234828 | IQCM | 1 | negative |
| ENSG00000235098 | ANKRD65 | 1 | negative |
| ENSG00000237190 | CDKN2AIPNL | 1 | negative |
| ENSG00000239789 | MRPS17 | 1 | negative |
| ENSG00000239998 | LILRA2 | 1 | negative |
| ENSG00000240045 | DWORF | 1 | negative |
| ENSG00000240344 | PPIL3 | 1 | negative |
| ENSG00000240563 | L1TD1 | 1 | negative |
| ENSG00000240583 | AQP1 | 1 | negative |
| ENSG00000242220 | TCP10L | 1 | negative |
| ENSG00000242498 | ARPIN | 1 | negative |
| ENSG00000242715 | CCDC169 | 1 | negative |
| ENSG00000243509 | TNFRSF6B | 1 | negative |
| ENSG00000243708 | PLA2G4B | 1 | negative |
| ENSG00000244005 | NFS1 | 1 | negative |
| ENSG00000244687 | UBE2V1 | 1 | negative |
| ENSG00000244694 | PTCHD4 | 1 | negative |
| ENSG00000247077 | PGAM5 | 1 | negative |
| ENSG00000248405 | PRR5-ARHGAP8 | 1 | negative |
| ENSG00000249437 | NAIP | 1 | negative |
| ENSG00000249853 | HS3ST5 | 1 | negative |
| ENSG00000251192 | ZNF674 | 1 | negative |
| ENSG00000251247 | ZNF345 | 1 | negative |
| ENSG00000253719 | ATXN7L3B | 1 | negative |
| ENSG00000254440 | PBOV1 | 1 | negative |
| ENSG00000254996 | ANKHD1-EIF4EBP3 | 1 | negative |
| ENSG00000256053 | APOPT1 | 1 | negative |
| ENSG00000257017 | HP | 1 | negative |
| ENSG00000257727 | CNPY2 | 1 | negative |
| ENSG00000258947 | TUBB3 | 1 | negative |
| ENSG00000260230 | FRRS1L | 1 | negative |
| ENSG00000261652 | C15orf65 | 1 | negative |
| ENSG00000265808 | SEC22B | 1 | negative |
| ENSG00000265972 | TXNIP | 1 | negative |
| ENSG00000266028 | SRGAP2 | 1 | negative |
| ENSG00000266338 | NBPF15 | 1 | negative |
| ENSG00000267041 | ZNF850 | 1 | negative |
| ENSG00000267855 | NDUFA7 | 1 | negative |
| ENSG00000273590 | SMIM11B | 1 | negative |
| ENSG00000273611 | ZNHIT3 | 1 | negative |
| ENSG00000275023 | MLLT6 | 1 | negative |
| ENSG00000275835 | TUBGCP5 | 1 | negative |
| ENSG00000276293 | PIP4K2B | 1 | negative |
| ENSG00000277363 | SRCIN1 | 1 | negative |
| ENSG00000277791 | PSMB3 | 1 | negative |
| ENSG00000278129 | ZNF8 | 1 | negative |
| ENSG00000278259 | MYO19 | 1 | negative |
| ENSG00000278619 | MRM1 | 1 | negative |
| ENSG00000278845 | MRPL45 | 1 | negative |
| ENSG00000285437 | POLR2J3 | 1 | negative |
